# Ceramide synthase 4 overexpression exerts oncogenic properties in breast cancer

**DOI:** 10.1186/s12944-023-01930-z

**Published:** 2023-10-26

**Authors:** Su-Jeong Kim, Incheol Seo, Min Hee Kim, Joo-Won Park, Shin Kim, Woo-Jae Park

**Affiliations:** 1 Department of Biochemistry, Chung-Ang University College of Medicine, Heukseok-lo 84, DongJak-gu, Seoul, 06974 Republic of Korea; 2 Department of Immunology, Kyungpook National University School of Medicine, Daegu, 41944 Republic of Korea; 3 Department of Biochemistry, College of Medicine, Ewha Womans University, Seoul, 07804 Republic of Korea; 4 Department of Immunology, School of Medicine, Keimyung University, Dalgubeol-daero 1095, Dalseo-gu, Daegu, 42601 Republic of Korea

**Keywords:** Ceramide synthase 4, Ceramide acyl chain length, Oncogene, Epithelial mesenchymal transition, Chemoresistance, Luminal subtype a breast cancer

## Abstract

**Background:**

Ceramide, a bioactive signaling sphingolipid, has long been implicated in cancer. Members of the ceramide synthase (CerS) family determine the acyl chain lengths of ceramides, with ceramide synthase 4 (CerS4) primarily generating C18–C20-ceramide. Although CerS4 is known to be overexpressed in breast cancer, its role in breast cancer pathogenesis is not well established.

**Methods:**

To investigate the role of CerS4 in breast cancer, public datasets, including The Cancer Genome Atlas (TCGA) and two Gene Expression Omnibus (GEO) datasets (GSE115577 and GSE96058) were analyzed. Furthermore, MCF-7 cells stably overexpressing CerS4 (MCF-7/CerS4) as a model for luminal subtype A (LumA) breast cancer were produced, and doxorubicin (also known as Adriamycin [AD])-resistant MCF-7/ADR cells were generated after prolonged treatment of MCF-7 cells with doxorubicin. Kaplan–Meier survival analysis assessed the clinical significance of *CERS4* expression, while Student’s t-tests or Analysis of Variance (ANOVA) compared gene expression and cell viability in different MCF-7 cell lines.

**Results:**

Analysis of the public datasets revealed elevated *CERS4* expression in breast cancer, especially in the most common breast cancer subtype, LumA. Persistent CerS4 overexpression in MCF-7 cells activated multiple cancer-associated pathways, including pathways involving sterol regulatory element–binding protein, nuclear factor kappa B (NF-κB), Akt/mammalian target of rapamycin (mTOR), and β-catenin. Furthermore, MCF-7/CerS4 cells acquired doxorubicin, paclitaxel, and tamoxifen resistance, with concomitant upregulation of ATP-binding cassette (ABC) transporter genes, such as *ABCB1*, *ABCC1*, *ABCC2*, *ABCC4*, and *ABCG2*. MCF-7/CerS4 cells were characterized by increased cell migration and epithelial–mesenchymal transition (EMT). Finally, *CERS4* knockdown in doxorubicin-resistant MCF-7/ADR cells resulted in reduced activation of cancer-associated pathways (NF-κB, Akt/mTOR, β-catenin, and EMT) and diminished chemoresistance, accompanied by *ABCB1* and *ABCC1* downregulation.

**Conclusions:**

Chronic CerS4 overexpression may exert oncogenic effects in breast cancer via alterations in signaling, EMT, and chemoresistance. Therefore, CerS4 may represent an attractive target for anticancer therapy, especially in LumA breast cancer.

**Supplementary Information:**

The online version contains supplementary material available at 10.1186/s12944-023-01930-z.

## Background

Breast cancer is one of the most common malignancies worldwide, associated with high mortality among women [1]. Breast cancer can be classified based on the expression status of estrogen receptor (ER), progesterone receptor (PGR), human epidermal growth factor receptor 2 (HER2), and Ki67 [2]. Molecular breast cancer subtypes include normal-like (ER^+^, PGR^+^, HER2^−^, Ki67^−^), basal (ER^−^, PGR^−^, HER2^−^, basal marker^+^), luminal subtype A (LumA; ER^+^, PGR^+^, HER2^−^, Ki67^−^), luminal subtype B (LumB; ER^+^, PGR^+^, HER2^−^, Ki67^+^), HER2-positive (ER^−^, PGR^−^, HER2^+^), and triple-negative (ER^−^, PGR^−^, HER2^−^) [2]. Accounting for 50–60% of all breast cancer diagnoses [3], LumA is the most prevalent subtype and is associated with relatively good clinical outcomes and prognosis; however, a mechanistic understanding of LumA pathogenesis and the development of novel chemotherapies remain critical.

Various cancer pathways, such as nuclear factor kappa B (NF-κB), protein kinase B (Akt)/mammalian target of rapamycin (mTOR), and Wnt/β-catenin, play crucial roles in breast cancer pathogenesis [4–6]. Specifically, NF-κB activation, which is frequently observed in breast cancer, promotes the development of a hormone-independent and invasive breast cancer phenotype [7], whereas its inhibition reverses endocrine resistance [4]. Activation of the Akt/mTOR pathway, which regulates various cellular activities, including protein synthesis, metabolic regulation, cell survival, and differentiation [8], facilitates tumor growth and breast cancer cell survival [9]. Activation of the Wnt/β-catenin pathway affects breast cancer cell migration and metastasis [6]. Sterol regulatory element–binding proteins (SREBPs) are transcription factors that regulate fatty acid and cholesterol synthesis [10] and are known to regulate breast cancer cell invasion and migration [11, 12].

Metastasis and chemotherapy resistance are leading causes of death among breast cancer patients. The epithelial–mesenchymal transition (EMT), during which polarized epithelial cells transition into motile mesenchymal cells, allows breast tumors to become more invasive and malignant, with enhanced stem cell properties [13]. In addition, the overexpression of ATP-binding cassette (ABC) transporters confers chemotherapy resistance by expelling various drugs from tumor cells at the expense of ATP hydrolysis and contributes to breast cancer progression and metastasis through mechanisms independent of this efflux function [14]. All of these pathways and molecular mechanisms are potential therapeutic targets in breast cancer.

Sphingolipids are implicated in several pathophysiological processes associated with cancer development, progression, metastasis, and drug resistance [15]. Ceramide, located at the center of sphingolipid metabolism, acts as a biologically active lipid that determines cell fate decisions, including apoptosis, proliferation, and differentiation [16, 17]. Recent reports describe distinct roles played by ceramide species with different acyl chain lengths in various molecular pathways, including endoplasmic reticulum stress [18] and apoptosis [19]. In mammals, the acyl chain lengths of ceramides are determined by six ceramide synthase (CerS) family members [16, 17]. CerS1 and CerS2 generate C18-ceramide and C22–C24-ceramide, respectively. CerS4 primarily synthesizes C18–C20-ceramide, whereas CerS5 and CerS6 primarily produce C14–C16-ceramide [20]. In human breast cancer, the mRNA expression levels of *CERS2*, *CERS4*, and *CERS6* and their respective products, C16-, C24-, and C24:1-ceramides, are increased [21, 22]. In addition, alternative splicing of CerS2 in LumB breast cancer promotes cancer cell proliferation and migration and is associated with poor prognosis [23]. Thus, alterations in CerS activity or ceramide acyl chain lengths may play critical roles in breast cancer development and progression.

Unlike prior studies that have investigated the roles played by CerS family members in breast cancer using transient overexpression [24], in the present study, the molecular effects of long-term CerS4 overexpression on breast cancer progression and migration were explored using TCGA-BRCA data, MCF-7 cells stably overexpressing CerS4 (MCF-7/CerS4), and doxorubicin-resistant MCF-7 (MCF-7/ADR) cells.

## Methods

### Materials

The following substances were obtained: doxorubicin, paclitaxel, G418, anti-HA (H6908), anti-CerS2 (HPA027262), anti-CerS4 (SAB4301210), anti-active-β-catenin (05-665), and anti-α-tubulin antibodies (T9026) (Sigma-Aldrich, St. Louis, MO, USA); anti-phospho-NF-κB p65 (3033), anti-NF-κB p65 (8242), anti-Relb (4922), anti-phospho-p44/42 MAPK (ERK1/2) (4370), anti-phospho-p90RSK (11989), anti-phospho-Akt (9271), anti-phospho-mTOR (5536), anti-phospho-p70 S6 kinase (9205), anti-ERα (8644), anti-phospho-ERα (Ser118) (2511), anti-phospho-ERα (Ser167) (64508), anti-PGR (8757), anti-vimentin (5741), anti-Snail (3879), anti-lamin A/C (2032), β-catenin (9562), and anti-phospho-GSK3β (5558) antibodies (Cell Signaling Biotechnology, Inc, Beverly, MA, USA); anti-LASS6 (CerS6) (sc-100554), anti-E-cadherin (sc-71008), and anti-N-cadherin (sc-59987) antibodies (Santa Cruz Biotechnology, Santa Cruz, CA, USA); anti-CerS1 antibody (H00010715-M01) (Abnova, Taipei, Taiwan); anti-GAPDH (glyceraldehyde 3-phosphate dehydrogenase) antibody (MAB374) (EMD Millipore, Billerica, MA, USA).

### Cell culture

MCF-7 cells (RRID:CVCL_0031), a human breast cancer cell line, were cultured in RPMI-1640 medium (Hyclone Laboratories, Logan, UT, USA) containing 1% penicillin/streptomycin and 10% fetal bovine serum (FBS, Hyclone Laboratories). The cells were incubated at 37℃ in a humidified atmosphere with 5% CO_2_. MCF-7/CerS4 cells were cultured in RPMI-1640 medium, 1% penicillin/streptomycin, 10% FBS (Hyclone Laboratories), and 100 µg/ml G418 (Sigma-Aldrich).

### Transfection and stable cell line generation

The MCF-7 cells were transfected with 4 µg of the pcDNA3.1-CerS4-HA, using 150 mM NaCl and Lipidofect-P Transfection Reagent (Lipidomia, Seongnam, Korea). Thereafter, G418 was used to create permanently stable transfected cell lines. At first, 300 µg/ml of G418 was strongly treated for selection, and the surviving cells were maintained in fresh RPMI-1640 medium containing 100 µg/ml of G418. G418 was removed 48 h before experiments. The MCF-7/ADR cells were transfected with 3 µg of the pSUPER-shCerS4. Transfected vectors such as pcDNA3.1-CerS4-HA and pSUPER-shCerS4 were provided from Professor A.H. Futerman (Weizmann Institute of Science, Rehovot, Israel).

### Generation of doxorubicin-resistant cell lines

MCF-7/ADR cells were generated by prolonged treatment with doxorubicin at increasing concentrations (0.5 ~ 25 µM) for 6–8 months, as previously described [25].

### Western blot analysis

The MCF-7, MCF-7/CerS4, and MCF-7/ADR cells were lysed by RIPA buffer (50 mM Tris-Cl; pH 7.5, 150 mM NaCl, 0.1% sodium dodecyl sulfate [SDS], 0.5% sodium deoxycholate, 1% Triton X-100 or Nonidet P-40 (NP-40), protease inhibitors, and phosphatase inhibitors). Cell lysates were incubated on ice for 30 min, and then the supernatants were obtained by centrifugation at 12,000 ×g for 15 min at 4℃. Quantification of the lysed protein was measured with Bio-Rad Protein Assay Dye Reagent (Bio-Rad Laboratories, Hercules, CA, USA). Afterward, 30 ~ 50 µg of the heat-denatured protein was loaded and separated by 10% SDS-PAGE. Separated proteins were transferred to a nitrocellulose membrane (Bio-Rad Laboratories), which was further blocked by 5% bovine serum albumin (BSA, Sigma-Aldrich) in TBST (TBS with 0.1% Tween 20) for 1 h. The membrane was incubated on a shaking incubator at 4℃ overnight to attach the primary antibody. Then, after washing with TBST, the secondary antibody was attached at room temperature for 1 h. Both primary and secondary antibodies were dissolved in TBST. Blots on the membrane were detected using ECL western blotting detection solution (ATTO), and then a digitized image of the membrane was captured using the ChemiDoc MP imaging system (Bio-Rad Laboratories).

### RNA isolation and reverse transcription-real-time PCR

Total mRNA of MCF-7 cells and MCF-7/CerS4 cells was extracted using RNeasy Mini Kits (Qiagen, Valencia, CA, USA), and mRNA was quantified using the NanoDrop 1000 spectrophotometer (Thermo Scientific, Wilmington, Denmark). To synthesize cDNA from mRNA, the Verso cDNA Synthesis Kit (Thermo Scientific) was used. To perform real-time PCR, a total volume of 10 µl containing 0.1 µg cDNA, 10 pmol forward primer and reverse primer, and SYBR-Green Master Mix (Thunderbird) was used. The analysis was performed using the CFX Connect Real-time PCR Detection System (Bio-Rad Laboratories). Thermal cycling conditions consisted of 1 min at 95℃, followed by 40 cycles of 15 s at 95℃ and 45 s at 60℃. The primers used in this paper are summarized in Supplementary Table S1.

### MTT assay

Viabilities of MCF-7 cells and MCF-7/CerS4 cells were measured by 3-(4,5-dimethylthiazol-2-yl)-2,5-diphenyltetrazolium bromide (MTT) assay. The cells were seeded at 5 × 10^4^ in 96-well plates. After the cells had adhered, doxorubicin, paclitaxel, and tamoxifen were treated at different concentrations (0 ~ 50 µM) for 2 days. Then, MTT solution (5 mg/ml in phosphate-buffered saline [PBS]) was added to each well and incubated at 37℃ for 2–3 h until a purple precipitate was visible. Finally, 10% SDS was added to each well, and the absorbance was measured at 570 nm.

### Transwell invasion assay with Matrigel

A transparent PET membrane insert with an 8.0 μm hole was inserted into a 24-well plate. For coating of inserts, Matrigel and FBS-free medium were mixed in a ratio of 1:6. Then, 40–50 µl of the mixture was placed on the insert and incubated overnight in a 37℃ incubator for drying. After incubation, 750 µl of medium with 10% FBS was placed in the bottom well, and 5 × 10^4^ cells in 200 µl of medium containing 0.5% FBS were added to the insert. After incubation for 48 h to allow cells to penetrate the lower wells, the cells were fixed using 100% methanol and stained with 0.4% trypan blue solution (Sigma-Aldrich) for 10 min. After washing with PBS, cell invasion was captured under a microscope.

### LC-ESI-MS/MS analysis of ceramides

Ceramide analyses by LC-ESI-MS/MS were conducted as described previously [18].

### RNA-sequencing (RNA-Seq) analysis

Gene expression profiles of MCF-7 and MCF-7/CerS4 cells were compared using RNA-Seq. Total mRNA was extracted using RNeasy Mini Kits (Qiagen) according to the manufacturer’s protocol. The raw sequencing data were generated by the Illumina NovaSeq platform (pair-end, 2 × 101 bp). FastQC was used to assess the quality of the raw sequencing reads. Then, sequencing reads were aligned to the GRCh38 reference genome using the STAR aligner in two-pass mode with default parameters [26]. DESeq2 analysis was performed to identify differentially expressed genes (DEGs). Statistically significant DEGs were defined with adjusted *P* < 0.0001 and |log_2_FC| > 1.5.

### Analysis of TCGA-BRCA and GEO dataset

To compare *CERS4* expression between normal and cancer or LumA and non-luminal subtype A (non-LumA) breast cancer, TCGA-BRCA, GSE115577, and GSE96058 data were used. STAR raw count data for TCGA-BRCA were obtained in R using the TCGAbiolinks package. Then, the raw count was normalized using DESeq2 [27]. The RMA-normalized probe intensities were obtained for GSE115577 in R using the GEOquery package [28]. The FPKM expression matrix for GSE96058 was obtained from GEO. The molecular subtypes were obtained from metadata in each dataset [29]. Kaplan−Meier survival analysis was performed to investigate the relationship between *CERS4* expression and overall survival of the patient with LumA breast cancer. The optimal cut-off value for survival analysis was determined using the survminer package in R. GSE116436 was used to investigate changes in *CERS4* expression in MCF-7 cells after various treatments with a chemotherapeutic agent. The gene expression profiles and EMT score of doxorubicin-resistant MCF-7/ADR cells were investigated using MCF-7/ADR dataset (GSE24460). The RMA-normalized probe intensities were obtained, and the limma package was used in R for DEG analysis [30]. Statistically significant DEGs were defined with adjusted *P* < 0.01 and |log_2_FC| > 1.

### Bioinformatic and statistical analyses

All experiments were independently repeated three times, and the values were expressed as the mean ± standard error of the mean. Half maximal inhibitory concentration (IC50) for doxorubicin, paclitaxel, and tamoxifen was calculated using the drm package in R [31]. The two-tailed Student *t-*test was performed to compare individual gene expression or cell viability between different MCF-7 cell lines with P < 0.05. For comparisons involving more than three cell lines, multiple time points, or treatment dosages, one- or two-way Analysis of Variance (ANOVA), and a Tukey post hoc test were performed. The functional annotation of DEGs was performed using Enrichr [32]. The EMT score was calculated from RNA-Seq or microarray by the sum of the expression of nine well-known mesenchymal marker genes minus the total expression of five known epithelial genes, as previously described [33]. Bioinformatic and statistical analyses were performed using R version 4.2.1.

## Results

### Expression of ***CERS4*** and survival analysis across breast cancer cohorts

**Fig. 1:**
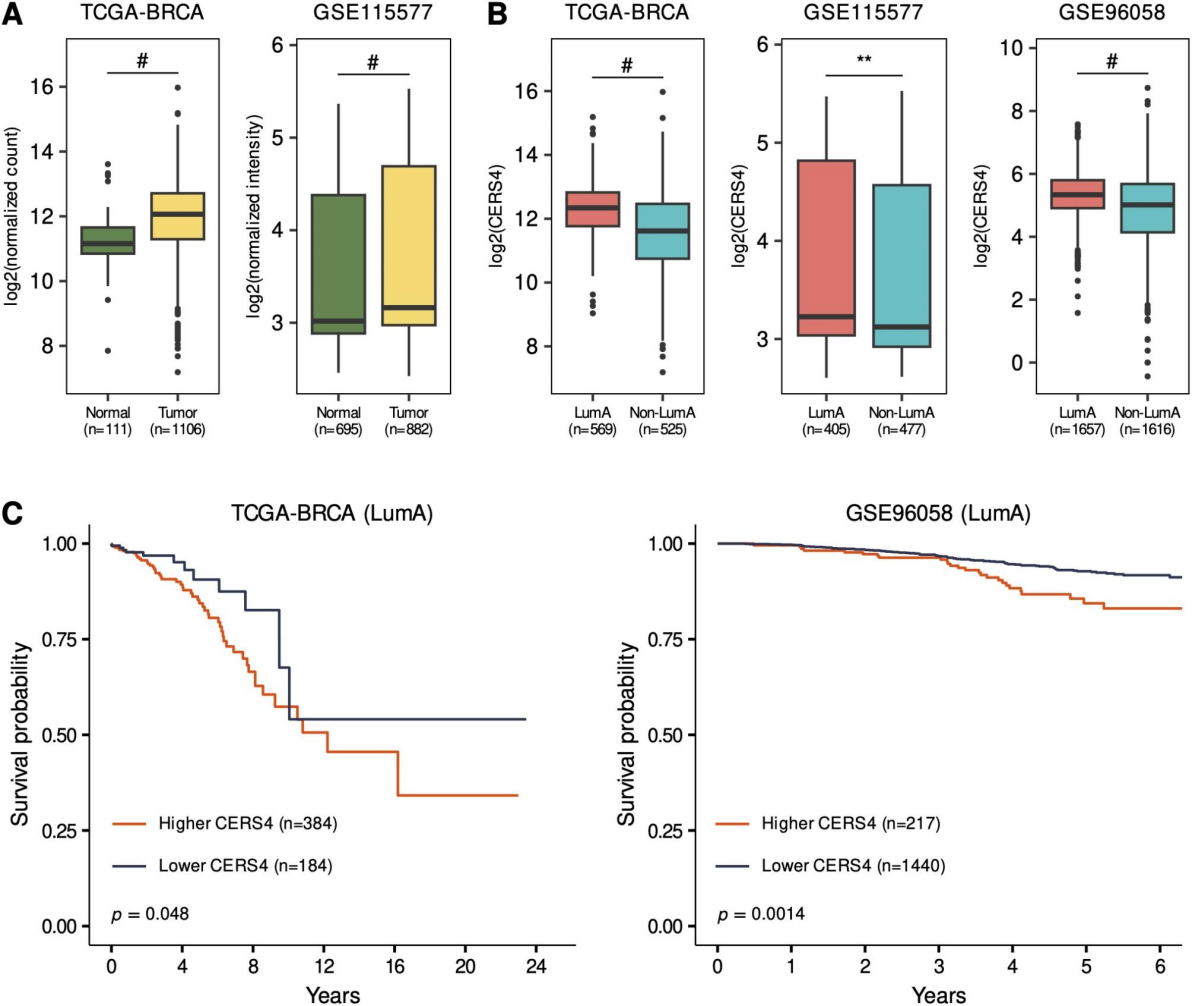
*CERS4* expression levels and prognostic significance in patients with breast cancer. **(A)***CERS4* expression in breast cancer tissue (tumor) and adjacent normal breast tissue. **(B)***CERS4* expression in LumA and PAM50 molecular breast cancer subtypes. **(C)** Kaplan−Meier plot showing survival among individuals with LumA breast cancer in the higher and lower *CERS4* expression groups. The number of subjects is shown in parentheses. Two-tailed Student’s *t*-test was performed for (A and B). The log-rank test was performed for **(C).** Significance is indicated as follows: ^**^P < 0.01 and ^#^*P* < 0.0001

Although elevated mRNA levels have been reported for *CERS2*, *CERS4*, and *CERS6* in human breast cancer [21, 22], the present study specifically focused on CerS4 because this protein was upregulated in not only TCGA-BRCA data but also MCF-7/ADR cells among all CerS proteins, indicating that CerS4 is expected to play an important role in breast cancer. To explore whether CerS4 expression is altered in breast cancer, The Cancer Genome Atlas Breast Invasive Carcinoma (TCGA-BRCA) and two Gene Expression Omnibus (GEO) datasets (GSE115577 and GSE96058) were analyzed. These analyses revealed significantly higher *CERS4* mRNA expression levels in tumor tissues than in normal tissues in two cohorts (TCGA-BRCA, *P* < 0.0001; GSE115577, *P* < 0.0001; Fig. 1A). To determine which breast cancer subtypes are characterized by elevated *CERS4* mRNA, the datasets were reanalyzed according to breast cancer subtype, revealing significantly higher *CERS4* mRNA expression in LumA than in non-LumA (LumB, HER2-positive, and basal) subtypes across all three cohorts (TCGA-BRCA, *P* < 0.0001; GSE115577, *P* = 0.007; GSE96058, *P* < 0.0001; Fig. 1B). To elucidate the relationship between *CERS4* expression and clinical prognosis in LumA breast cancer, Kaplan–Meier survival analyses were performed comparing LumA patients with higher *CERS4* expression and those with lower *CERS4* expression based on an optimal cutoff value. This analysis revealed that higher *CERS4* mRNA expression levels were associated with poor overall survival in LumA breast cancer (TCGA-BRCA, *P* = 0.048; GSE96058, *P* = 0.0014; Fig. 1C).

### Identification of the molecular factors involved in cell proliferation in CerS4-overexpressing MCF-7 cells

**Fig. 2:**
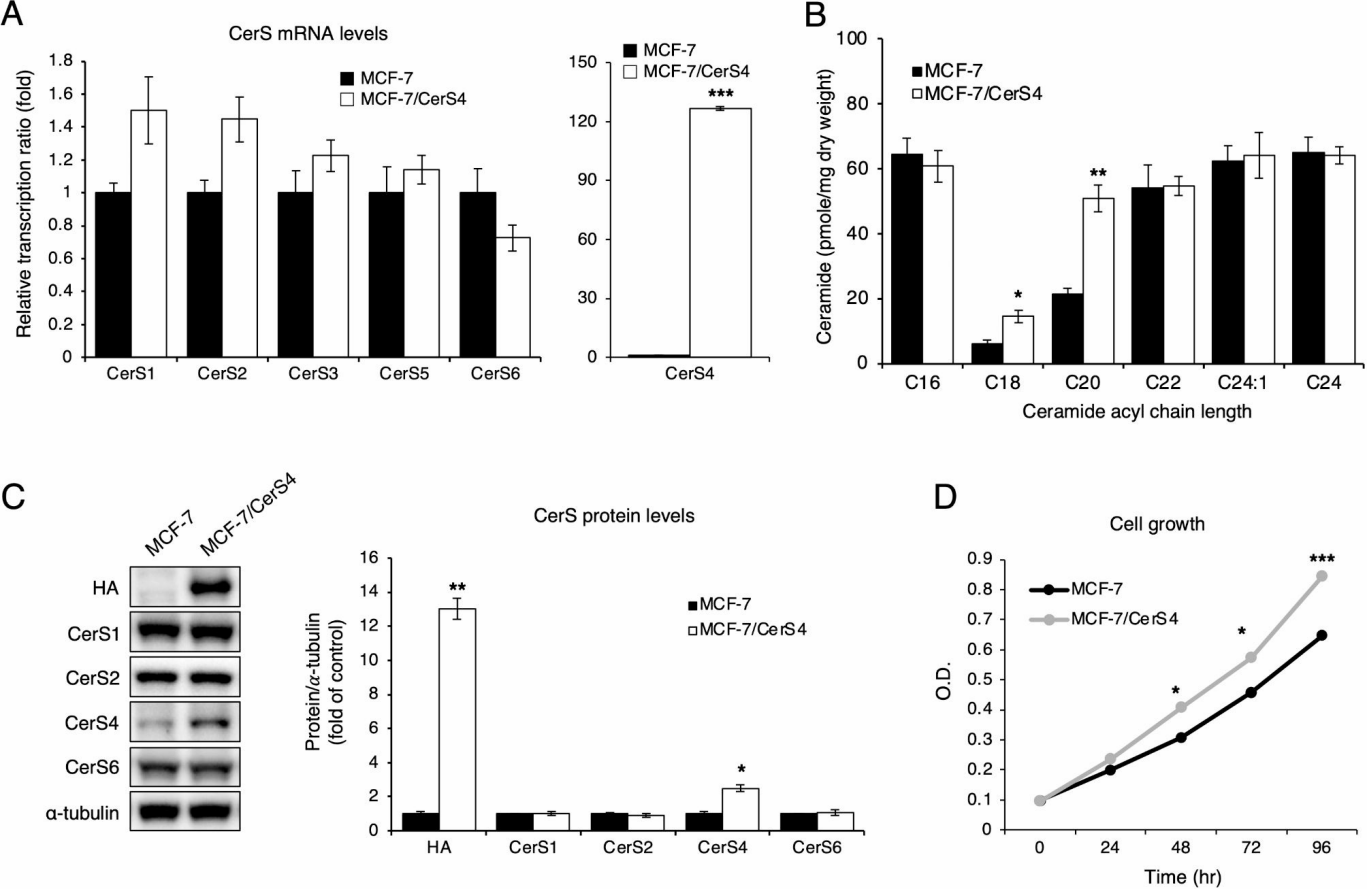
Establishment of a CerS4 overexpressing MCF-7 cell line. MCF-7 cells overexpressing CerS4 were selected by G418 treatment. **(A)** Relative mRNA expression levels of various CerS family members in MCF-7 and CerS4-overexpressing MCF-7 cells (MCF-7/CerS4) (n = 3). **(B)** Ceramide levels in MCF-7 and CerS4-overexpressing MCF-7 cells (MCF-7/CerS4) using liquid chromatography-electrospray ionization-tandem mass spectrometry (LC-ESI-MS/MS) (n = 3). **(C)** Representative western blots showing protein expression levels of various CerS family members (left) and densitometric analysis (right) of CerS protein levels in MCF-7 and MCF-7/CerS4 cells (n = 3). **(D)** Cell proliferation in MCF-7 and MCF-7/CerS4 cells (n = 3). A two-tailed Student’s *t*-test was performed for (A, B, and C). A two-way ANOVA and a Tukey post hoc test was performed for **(D)**. Significance is indicated as follows: ^*^P < 0.05, ^**^P < 0.01, and ^***^P < 0.001

To explore the effects of chronic CerS4 overexpression, CerS4 was stably overexpressed in MCF-7 cells (MCF-7/CerS4), which express ER and PGR and are considered representative of the LumA [34]. *CERS4* mRNA levels increased by approximately 120-fold, and CerS4 protein levels increased by approximately 2.3-fold in MCF-7/CerS4 cells compared with control MCF-7 cells, with no apparent alterations in the mRNA or protein levels of other CerS family members (Fig. 2A and C). CerS4 overexpression also increased the levels of C18- and C20-ceramides without changes in other ceramides (Fig. 2B). To examine the effect of persistent CerS4 overexpression on cell proliferation, cell growth was measured using the MTT cell viability assay. MCF-7/CerS4 cell proliferation was greater than that for control MCF-7 cells (Fig. 2D), and functional annotation of upregulated DEGs in MCF-7/CerS4 cells compared with control MCF-7 cells further showed activated cell cycle pathways (Table 1). These results suggest that CerS4 contributes to tumor cell proliferation by promoting cell cycle transitions.

**Table 1:** Functional annotation of upregulated genes in MCF-7/CerS4 cells using Reactome_2022

Reactome_2022 Term	Overlap	*P*-value	Adjusted *P*-value	Odds ratio	Combined score
Innate Immune System R-HSA-168,249	100 (1035)	1.80E − 12	2.36E-09	2.34569897	63.43460249
Immune System R-HSA-168,256	155 (1943)	7.02E − 12	4.60E-09	1.940958578	49.846957
Cell Cycle, Mitotic R-HSA-69,278	60 (523)	9.03E − 11	3.94E-08	2.781618301	64.33409133
Mitotic G1 Phase and G1/S Transition R-HSA-453,279	28 (147)	1.55E − 10	5.09E-08	4.960806124	112.0443945
Cell Cycle R-HSA-1,640,170	68 (654)	3.77E − 10	9.87E-08	2.497379682	54.19260145
Interleukin-4 and Interleukin-13 Signaling R-HSA-6,785,807	23 (107)	5.57E − 10	1.22E-07	5.751514433	122.5517755
G1/S Transition R-HSA-69,206	25 (129)	1.02E − 09	1.90E-07	5.055272471	104.6787947
G1/S-Specific Transcription R-HSA-69,205	12 (29)	2.29E − 09	3.75E-07	14.70086789	292.4689634
NGF-Stimulated Transcription R-HSA-9,031,628	13 (39)	1.11E − 08	1.35E-06	10.41958425	190.8686062

Functional annotation for the top 10 most significant of 927 differentially upregulated genes in MCF-7/CerS4, obtained using Enrichr against the Reactome_2022 library

**Fig. 3:**
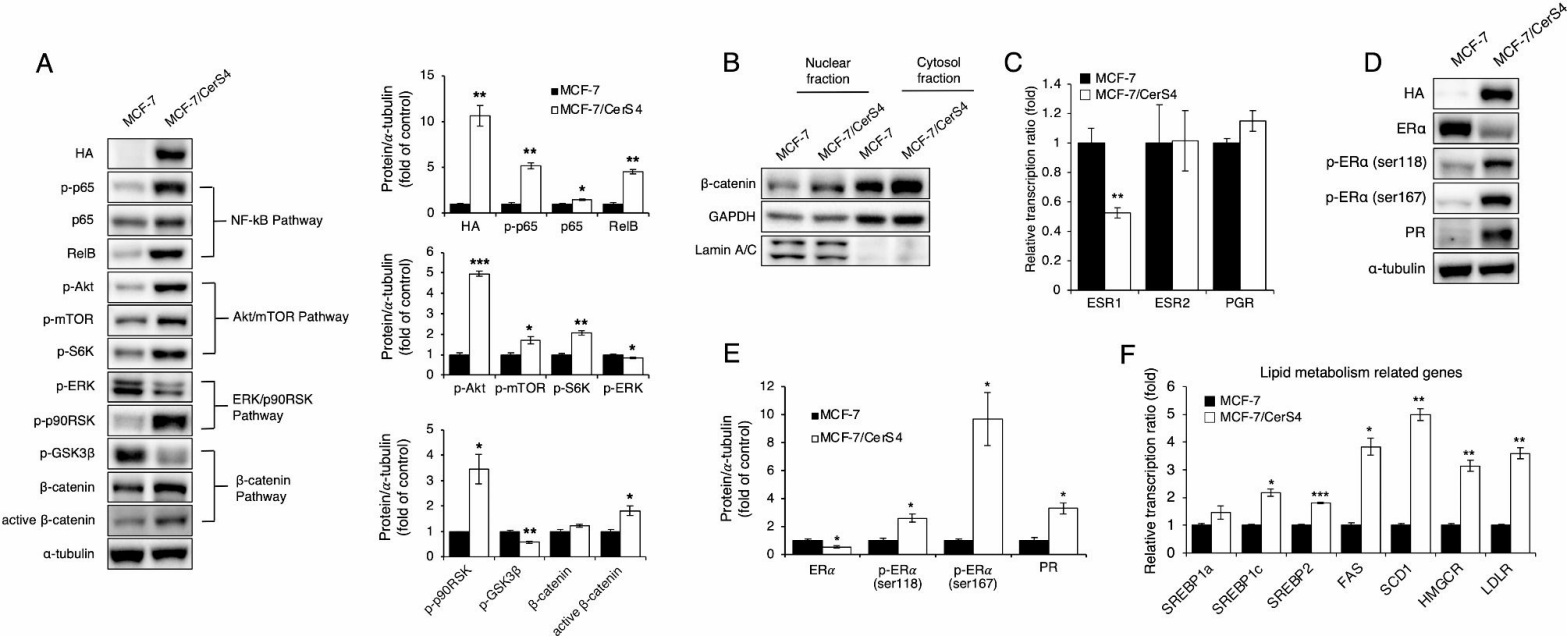
Multiple cancer-related pathways and receptors are activated in CerS4-overexpressing MCF-7 cells. **(A)** Well-known tumor-related pathway components (Akt/mTOR, NF-κB, and GSK3β/β-catenin) (left) and densitometric analysis (right) of the indicated protein in MCF-7 and MCF-7/CerS4 cells (n = 3) **(B)** β-catenin levels in nuclear and cytosol fractions were assessed in MCF-7 and CerS4-overexpressing MCF-7 cells (MCF-7/CerS4). **(C)** Relative *ESR1*, *ESR2*, and *PGR* mRNA expression levels in MCF-7 and MCF-7/CerS4 cells. **(D)** Representative western blots showing protein levels of ERα, PGR, and phosphorylated (Ser118, Ser167) ERα and (**E**) their densitometric analysis in MCF-7 and MCF-7/CerS4 cells (n = 3). **(F)** Increased expression of lipid-related genes in MCF-7/CerS4 cells compared with MCF-7 cells (n = 3). Two-tailed Student’s *t*-test was performed. Significance is indicated as follows: ^*^P < 0.05, ^**^P < 0.01, and ^***^P < 0.001. Akt, protein kinase B; ERα, estrogen receptor alpha; ERK, extracellular signal-related kinase; FAS, fatty acid synthase; GAPDH, glyceraldehyde 3-phosphate dehydrogenase; GSK3β, glycogen synthase kinase-3 betta; HA, hemi-agglutinin; HMGCR, 3-hydroxy-3-methylglutaryl-CoA reductase; LDLR, low-density lipoprotein receptor; mTOR, mammalian target of rapamycin; NF-κB, nuclear factor kappa B; PGR, progesterone receptor; S6K, ribosomal S6 kinase; SCD1, stearoyl-CoA desaturase 1; SREBP, sterol regulatory element–binding protein

To identify the molecular mechanisms involved in the increased proliferation observed for MCF-7/CerS4 cells compared with control MCF-7 cells, several proliferation-related signaling pathways were examined. NF-κB and Akt/mTOR activation and increased p90 ribosomal S6 kinase (p90RSK) phosphorylation were observed in MCF-7/CerS4 cells compared with control MCF-7 cells, whereas extracellular signal-regulated kinase (ERK) phosphorylation was reduced (Fig. 3A), implying that p90RSK activation occurred independent of ERK activation. In MCF-7/CerS4 cells, glycogen synthase kinase-3β (GSK3β) phosphorylation was accompanied by β-catenin activation (Fig. 3A). In MCF-7/CerS4 cells, β-catenin levels in both the nucleus and the cytosol increased compared with levels in control MCF-7 cells (Fig. 3B), revealing β-catenin pathway activation in the presence of persistent CerS4 overexpression.

Because MCF-7 cells are positive for both ER and PGR [35], the potential alterations in the expression of certain hormone receptors, including ERα (*ESR1*), ERβ (*ESR2*), and PGR (*PGR*), which are clinically important for predicting endocrine therapy efficacy, following persistent CerS4 overexpression were evaluated. *ESR1* mRNA levels were significantly reduced in MCF-7/CerS4 cells compared with control MCF-7 cells, whereas the expression levels of other genes, such as *ESR2* and *PGR*, were not altered (Fig. 3C). Consistent with lower *ESR1* mRNA levels, ERα protein levels were also reduced in MCF-7/CerS4 cells compared with control MCF-7 cells, and ERα phosphorylation increased at both Ser118 and Ser167 (Fig. 3D and E). Increased PGR protein expression was observed in MCF-7/CerS4 cells compared with control MCF-7 cells, despite unaltered *PGR* mRNA levels (Fig. 3C, D and E).

SREBPs regulate fatty acid and cholesterol metabolism [10], and their targets genes, such as fatty acid synthase (*FASN*) and stearoyl-CoA desaturase-1 (*SCD1*), are therapeutic targets in many cancers [36, 37]. Therefore, the potential effects of persistent CerS4 overexpression on the expression of SREBPs and their downstream targets, such as *FASN*, *SCD1*, 3-hydroxy-3-methylglutaryl coenzyme A (HMG-CoA) reductase (*HMGCR*), and low-density lipoprotein receptor (*LDLR*), were examined. The mRNA expression levels of SREBP-1c and SREBP-2 increased in MCF-7/CerS4 cells compared with control MCF-7 cells, accompanied by the upregulation of *FASN*, *SCD*, *HMGCR*, and *LDLR* (Fig. 3F). RNA-Seq analysis revealed that upregulated genes in MCF-7/CerS4 cells compared with control MCF-7 cells included well-known cancer-related transcription factors, such as *ERG*, *MYC*, *ESR1*, *TP53*, *SP1*, *EP300*, *RAD21*, *NFKB1*, *SMAD3*, and *HDAC2* (Table 2). These data suggest a potential role for CerS4 in breast cancer progression via transcriptional regulation.

**Table 2:** Functional annotation of upregulated genes in MCF-7/CerS4 cells using Transcription_Factor_PPIs.

Transcription_Factor_PPIs term	Overlap	*P*-value	Adjusted *P*-value	Odds ratio	Combined score
*ERG*	17 (41)	9.65E-13	2.39E − 10	14.82751832	410.2284253
*MYC*	93 (957)	8.40E-12	8.14E − 10	2.350115741	59.93357056
*ESR1*	87 (871)	9.85E-12	8.14E − 10	2.416094206	61.23323517
*TP53*	65 (628)	1.12E-09	6.97E − 08	2.47915748	51.08547827
*SP1*	37 (263)	1.68E-09	8.35E − 08	3.466933479	70.04192295
*EP300*	51 (473)	1.93E-08	7.97E − 07	2.573094527	45.70841512
*RAD21*	32 (237)	5.67E-08	2.01E − 06	3.29078076	54.90623716
*NFKB1*	33 (254)	8.98E-08	2.78E − 06	3.148774636	51.09018537
*SMAD3*	41 (375)	3.30E-07	9.09E − 06	2.59627134	38.74730225
*HDAC2*	40 (387)	1.92E-06	4.77E − 05	2.433615236	32.03028341

Functional annotation for the top 10 most significant of 927 differentially upregulated genes in MCF-7/CerS), obtained using Enrichr against the Transcription_Factor_PPIs library

### Multidrug-resistance (MDR) properties of CerS4-overexpressing MCF-7 cells

**Fig. 4:**
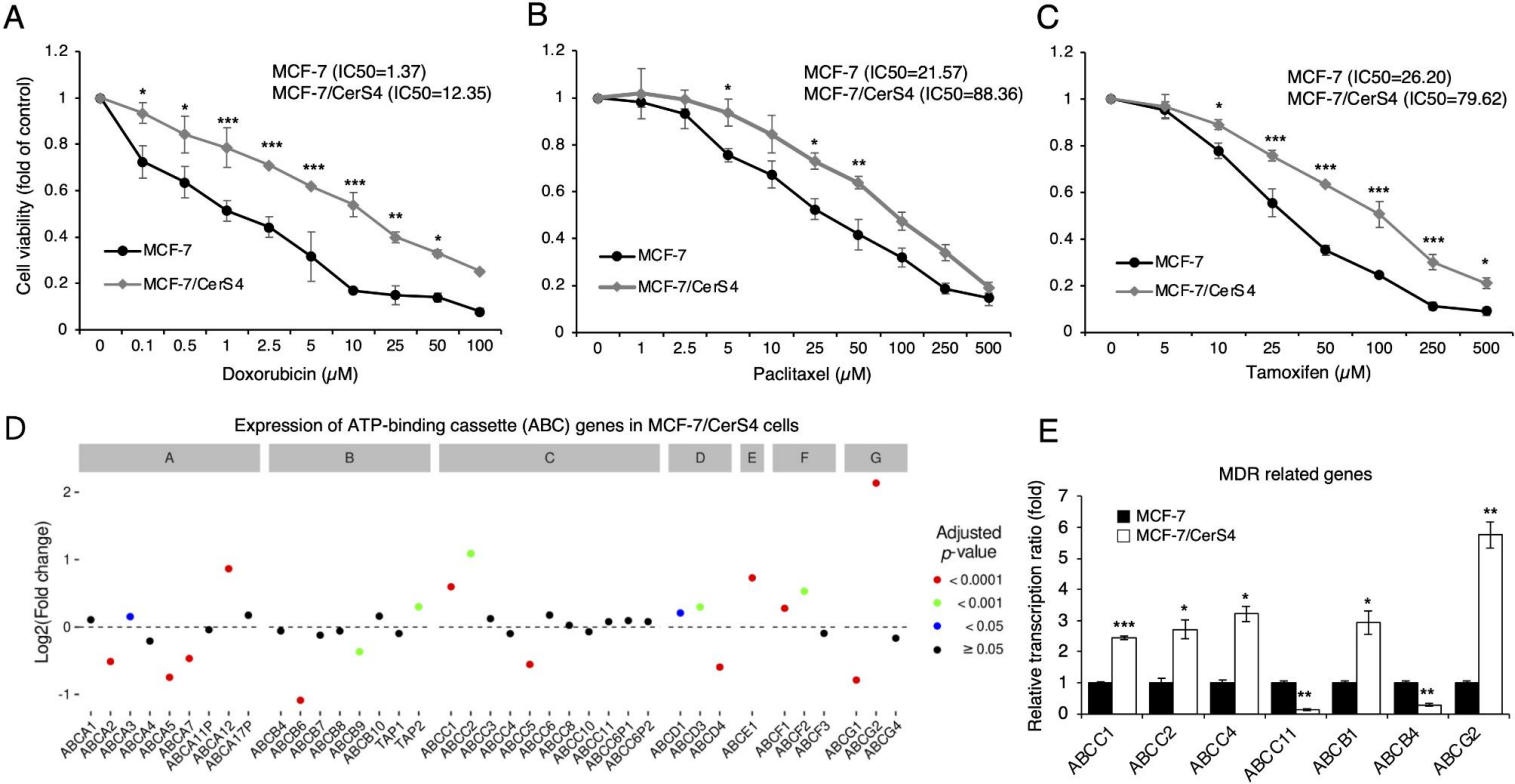
Increased resistance to chemotherapeutic agents in CerS4-overexpressing MCF-7 cells. **(A–C)** Cell death following treatment of MCF-7 and CerS4-overexpressing MCF-7 cells (MCF-7/CerS4) with **(A)** doxorubicin, **(B)** paclitaxel, or **(C)** tamoxifen (n = 3). IC50, half-maximal inhibitory concentration. **(D)** Expression of ATP-binding cassette (ABC) genes in MCF-7/CerS4 cells was assessed by RNA sequencing (n = 3). (**E**) Relative mRNA levels of ABC transporters (*ABCC1*, *ABCC2*, *ABCC4*, *ABCC11*, *ABCB1*, *ABCB4*, and *ABCG2*) was assessed by real-time PCR (n = 3). A two-way ANOVA and a Tukey post hoc test was performed for **(A, B, and C)**. The adjusted *P*-values obtained from DESeq2-analysis for **(D)**. A two-tailed Student’s *t*-test was performed for **(E)**. Significance is indicated as follows: ^*^P < 0.05, ^**^P < 0.01, and ^***^P < 0.001

Because chemotherapy failure can lead to cancer recurrence and death, the role of persistent CerS4 overexpression in the alteration of the chemoresistance properties of MCF-7 cells was examined by evaluating the impact of CerS4 overexpression on the responses of MCF-7 cells to the anticancer drugs doxorubicin, paclitaxel, and tamoxifen. Increased resistance to these three drugs in MCF-7/CerS4 cells compared with control MCF-7 cells was detected (Fig. 4A–C). To identify the molecular mechanisms involved in CerS4-induced chemoresistance, microarray data were analyzed to examine the expression profiles of various ABC transporter genes. As shown in Fig. 4D, the mRNA expression levels of *ABCA3*, *ABCA12*, *TAP2*, *ABCC1*, *ABCC2*, *ABCD1*, *ABCD3*, *ABCE1*, *ABCF1*, *ABCF2*, and *ABCG2* were significantly higher in MCF-7/CerS4 cells than in control MCF-7 cells, whereas the levels of *ABCA2*, *ABCA5*, *ABCA7*, *ABCB6*, *ABCB9*, *ABCC5*, *ABCD4*, and *ABCG1* were significantly reduced. Three groups of ABC transporters play major roles in chemoresistance: the classical P-glycoproteins (MDR1, ABCB1), the MDR-associated proteins (MRPs, in the ABCC subfamily), and ABCG2 (an ABC half-transporter) [38]. These gene expression results were then confirmed using real-time PCR. Consistent with increased chemoresistance following CerS4 overexpression (Fig. 4A–C), *ABCB1* (MDR1), *ABCC1* (MRP1), *ABCC2* (MRP2), *ABCC4* (MRP4), and *ABCG2* (breast cancer resistance protein) were upregulated in MCF-7/CerS4 cells compared with control MCF-7 cells, whereas *ABCB4* (MDR3) and *ABCC11* (MRP8) were downregulated (Fig. 4E).

### Cell migration and EMT properties in CerS4-overexpressing MCF-7 cells

**Fig. 5:**
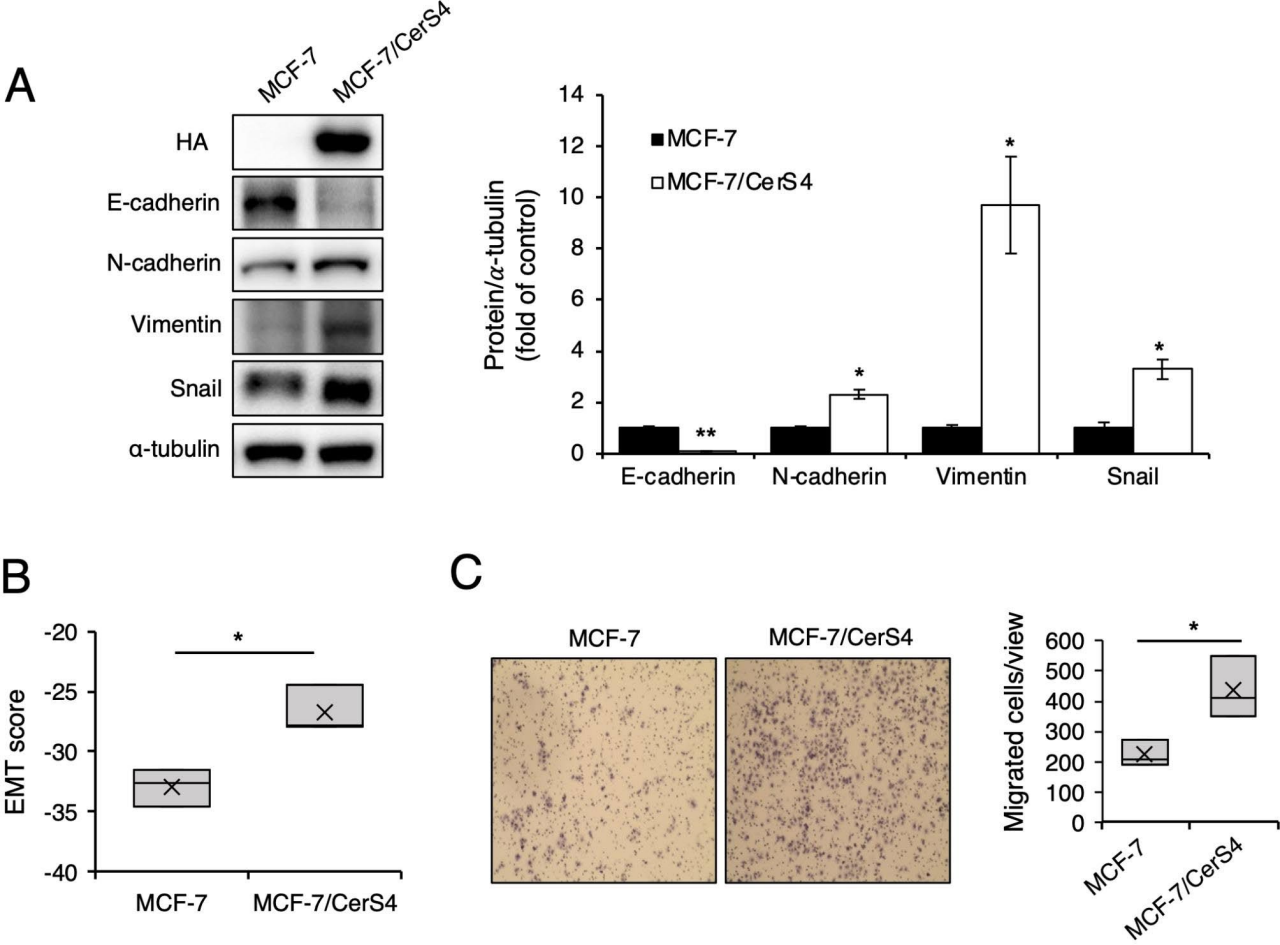
Expression of EMT markers is increased in CerS4-overexpressing MCF-7 cells. **(A)** Representative western blots of an epithelial marker (E-cadherin) and mesenchymal markers (N-cadherin, vimentin, and Snail) (left) and their densitometric analysis (right) in MCF-7 and MCF-7/CerS4 cells (n = 3). **(B)** EMT scores in MCF-7 and MCF-7/CerS4 cells (n = 3). **(C)** Cell migration in MCF-7 and MCF-7/CerS4 cells (n = 3). Two-tailed Student’s *t*-test was performed. Significance is indicated as follows :^*^P < 0.05, ^**^P < 0.01

The effect of CerS4 overexpression on the EMT process was analyzed in MCF-7 cells. The level of E-cadherin, an epithelial marker, were reduced in MCF-7/CerS4 cells compared with control MCF-7 cells, whereas the levels of mesenchymal cell markers, such as N-cadherin, vimentin, and Snail, increased (Fig. 5A). The EMT score was significantly higher in MCF-7/CerS4 cells than in control MCF-7 cells (Fig. 5B). In addition, cell migration increased in MCF-7/CerS4 cells compared with control MCF-7 cells (Fig. 5C). These data suggest that CerS4 overexpression causes MCF-7 cells to acquire the characteristics of migratory mesenchymal cells.

### Impacts of CerS4 in overcoming MDR in MCF-7 cells

**Fig. 6:**
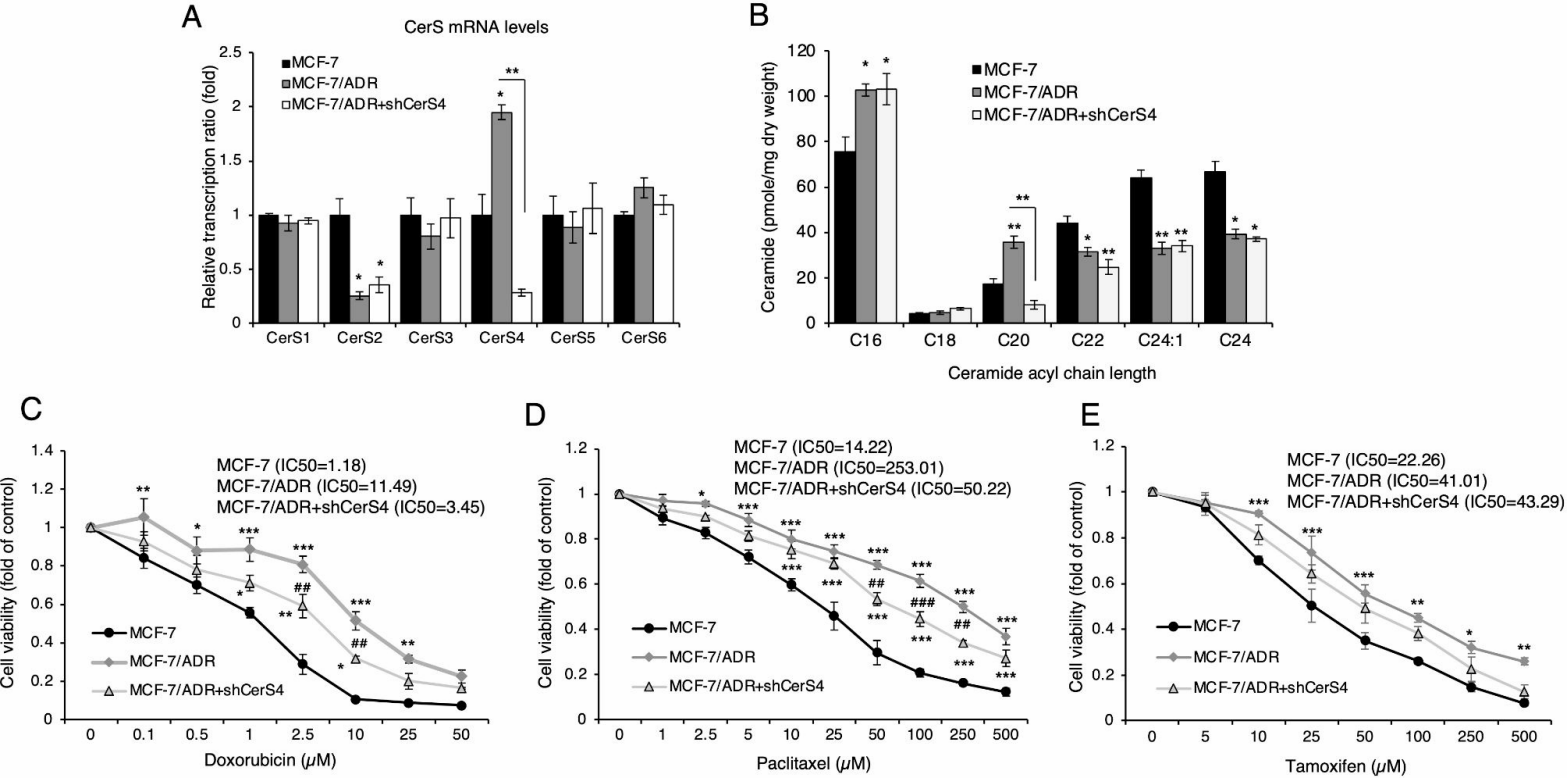
Downregulation of CerS4 in MCF-7/ADR cells partially reverses multiple drug resistance. **(A)** mRNA expression of various CerS family members in MCF-7 cells and MCF-7/ADR before and after (MCF-7/ADR + shCerS4) CerS4 downregulation (n = 3). **(B)** Ceramide levels in MCF-7 and MCF-7/ADR before and after (MCF-7/ADR + shCerS4) CerS4 downregulation using LC-ESI-MS/MS (n = 3). **(C–E)** Cell death was examined in MCF-7, MCF-7/ADR, and MCF-7/ADR + shCerS4 cells following treatment with (**C**) doxorubicin, (**D**) paclitaxel, or **(E)** tamoxifen (n = 3). IC50, half-maximal inhibitory concentration. A one-way ANOVA was performed for (A and B), and a two-way ANOVA was performed for **(C, D,** and **E)**, respectively. Additionally, a Tukey post hoc test was conducted for each analysis. Significance is indicated compared to control group; ^*^P < 0.05, ^**^P < 0.01, ^***^P < 0.001 and compared to MCF-7/ADR group; ^##^*P* < 0.01, ^###^*P* < 0.001

To test the potential that CerS4 may serve as a therapeutic target for overcoming MDR in breast cancer, CerS expression in Adriamycin (AD, also known as doxorubicin)-resistant MCF-7/ADR cells was analyzed. *CERS2* mRNA expression was reduced, whereas *CERS4* mRNA expression was significantly upregulated in MCF-7/ADR cells compared with control MCF-7 cells (Fig. 6A). Though C16- and C20-ceramide levels were elevated in MCF-7/ADR cells, C22-, C24-, and C24:1-ceramide levels were reduced (Fig. 6B). To elucidate whether CerS4 plays a role in the acquisition of chemoresistance, MCF-7/ADR cells were transfected with plasmids expressing CerS4-targeting shRNA (shCerS4). Reduced *CERS4* expression and C20-ceramide level following shCerS4 plasmid transfection were confirmed in MCF-7/ADR cells (Fig. 6A and B). CerS4 knockdown partially reversed drug resistance in MCF-7/ADR cells treated with doxorubicin, paclitaxel, or tamoxifen (Fig. 6C–E), implying a critical role for CerS4 in the acquisition of MDR in breast cancer.

**Fig. 7:**
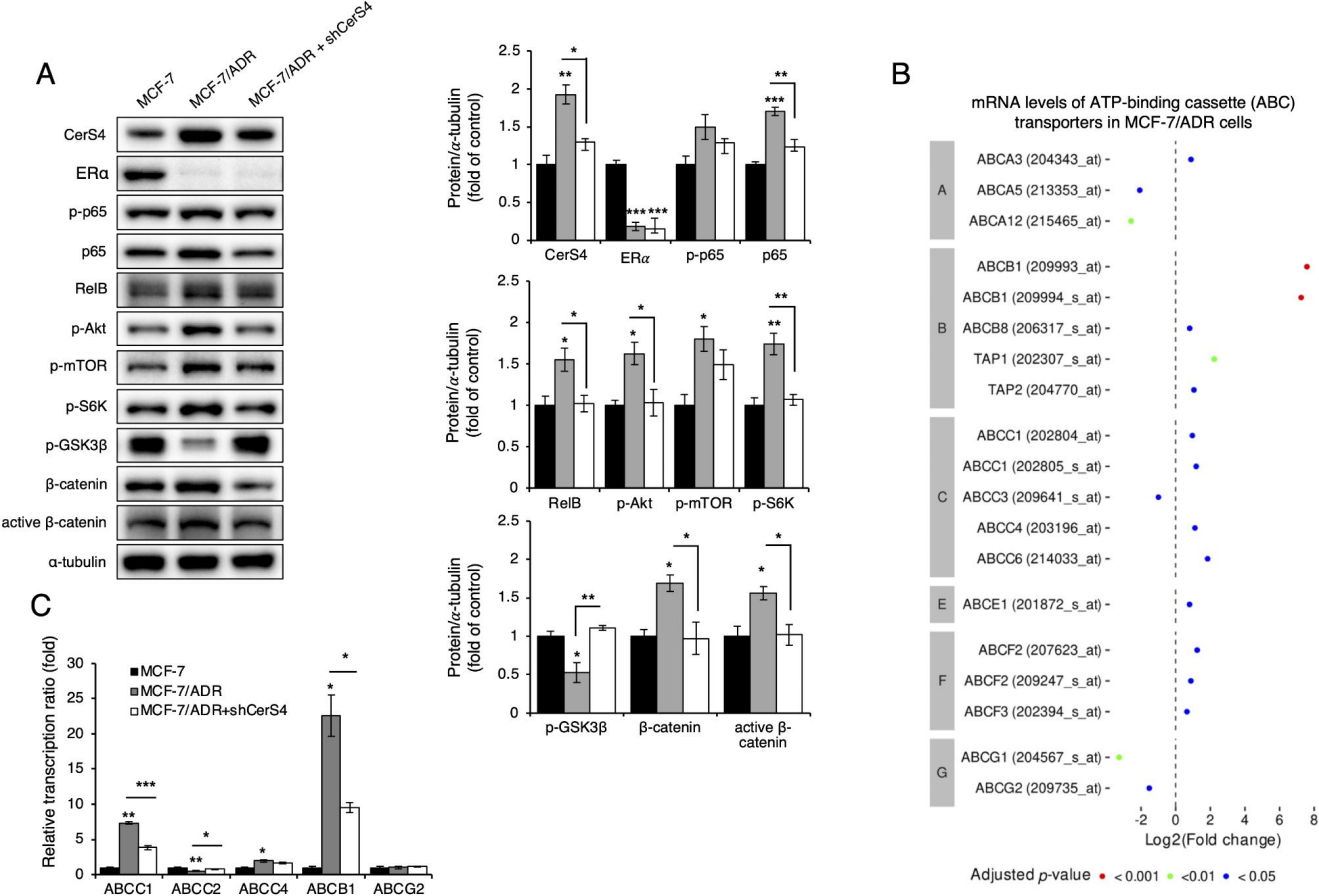
CerS4 downregulation in MCF-7/ADR cells partially reverses cancer-related pathway activation and increases ABC gene expression. **(A)** Representative western blot analysis of the indicated protein levels (left) and their densitometric analysis (right) in MCF-7 cells and MCF-7/ADR before and after (MCF-7/ADR + shCerS4) CerS4 downregulation (n = 3). **(B)** Relative mRNA levels of ATP-binding cassette (ABC) transporters in MCF-7/ADR cells, using data from GSE24460 (n = 2). **(C)** Relative mRNA levels of ABC transporters (*ABCC1*, *ABCC2*, *ABCC4*, *ABCB1*, and *ABCG2*) in MCF-7/ADR cells before and after (MCF-7/ADR + shCerS4) CerS4 downregulation (n = 3). A one-way ANOVA and a Tukey post hoc test was performed for (**A** and **C**). The adjusted *P*-values obtained from limma-analysis for **(B)**. Significance is indicated as follows: ^*^P < 0.05, ^**^P < 0.01, and ^***^P < 0.001. Akt, protein kinase B; GSK3β, glycogen synthase kinase-3 beta; mTOR, mammalian target of rapamycin; S6K, ribosomal S6 kinase

To further elucidate the molecular mechanisms through which CerS4 knockdown overcomes breast cancer chemoresistance, various signaling pathways associated with cell proliferation were examined. As shown in Fig. 7A, the NF-κB, Akt/mTOR, and β-catenin pathways were activated in MCF-7/ADR cells, and this activation was suppressed by CerS4 knockdown. However, downregulated ERα expression in MCF-7/ADR cells was not recovered by CerS4 knockdown. To explore whether CerS4 plays a role in the regulation of ABC transporter gene expression, microarray data were analyzed. *ABCA3*, *ABCB1* (209993_at and 209994_s_at), *ABCB8*, *TAP1* (202307_s_at), *TAP2* (204770_at), *ABCC1* (202804_at and 202805_s_at), *ABCC4*, *ABCC6* (214033_at), *ABCE1* (201872_s_at), *ABCF2* (209247_s_at), and *ABCF3* (202394_s_at) were significantly upregulated in MCF-7/ADR cells compared with control MCF-7 cells, whereas *ABCA5*, *ABCA12*, *ABCC3* (209641_s_at), *ABCG1* (204567_s_at), and *ABCG2* were downregulated (Fig. 7B). CerS4 knockdown partially attenuated the upregulation of *ABCB1* and *ABCC1* and restored *ABCC2* expression in MCF-7/ADR cells (Fig. 7C). These data suggest that CerS4 overexpression may overcome chemoresistance by modulating gene expression associated with proliferation-related signaling pathways and ABC transporters, such as MDR1 and MRP1.

**Fig. 8:**
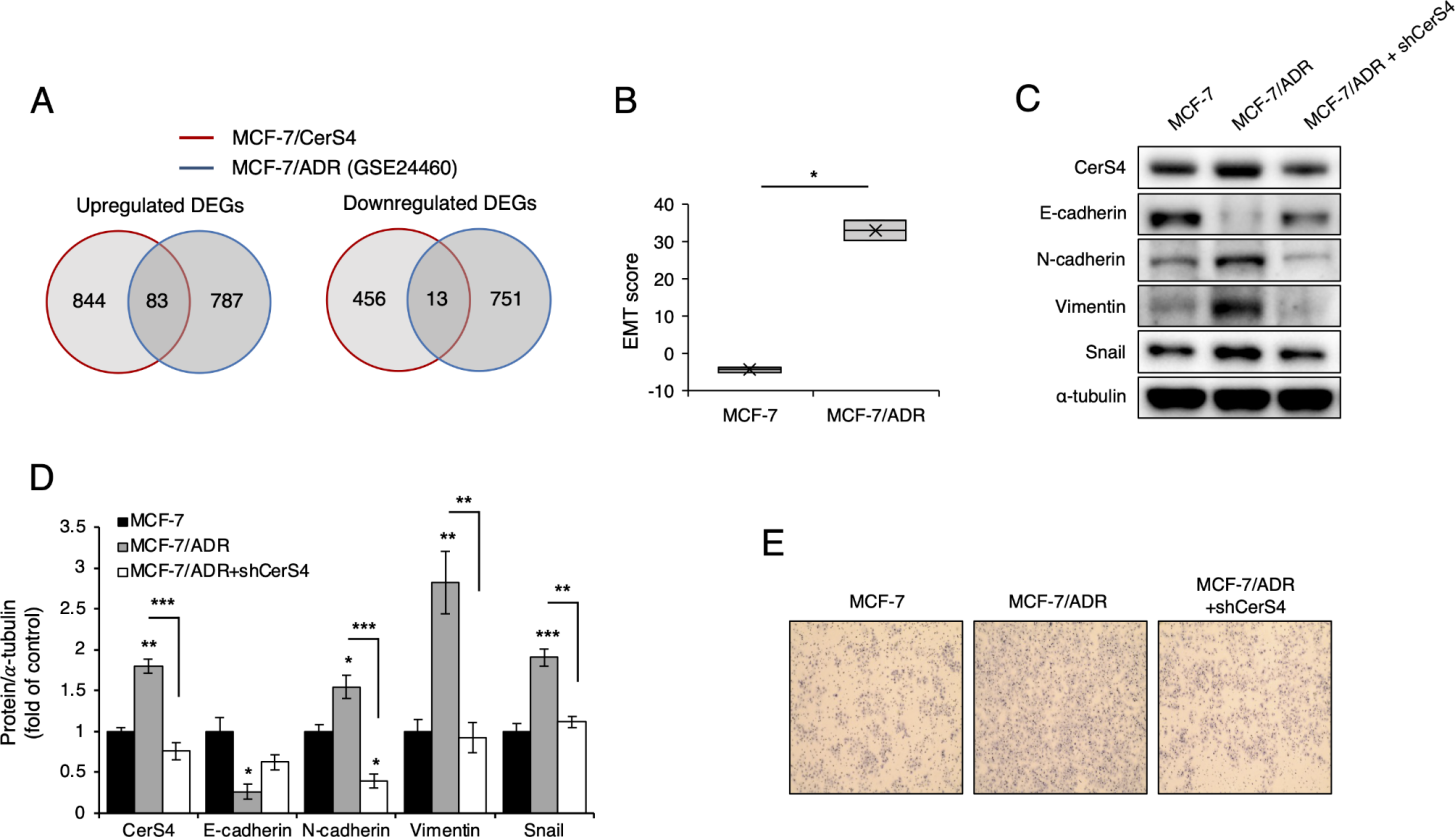
Downregulation of CerS4 in MCF-7/ADR cells reverses EMT. **(A)** The number of shared DEG between CerS4-overexpressing MCF-7 cells (MCF-7/CerS4) and MCF-7/ADR. **(B)** EMT score in MCF-7/ADR cells, calculated using data from GSE24460 (n = 2). **(C, D)** Representative western blots of an epithelial marker (E-cadherin) and mesenchymal markers (N-cadherin, vimentin, and Snail) **(C)** and their densitometric analysis **(D)** in MCF-7 cells and MCF-7/ADR cells before and after (MCF-7/ADR + shCerS4) CerS4 downregulation (n = 3). **(E)** Cell migration in MCF-7, MCF-7/ADR, and MCF-7/ADR + shCerS4 cells (n = 3). A two-tailed Student’s *t*-test was performed for (B). A one-way ANOVA and a Tukey post hoc test was performed for **(D)**. Significance is indicated as follows: ^*^P < 0.05, ^**^P < 0.01, and ^***^P < 0.001

To investigate whether targeting CerS4 hampers EMT progression in an MDR breast cancer cell model, microarray data were analyzed. A total of 83 upregulated and 13 downregulated DEGs were shared between MCF-7/CerS4 and MCF-7/ADR cells (GSE24460; Fig. 8A, Supplementary Table S2). Functional annotation of the 83 shared, upregulated DEGs revealed the upregulation of the EMT pathway in both MCF-7/CerS4 and MCF-7/ADR cells (Table 3). Similarly, the EMT score calculated using the MCF-7/ADR dataset (GSE24460) was higher for MCF-7/ADR cells than for control MCF-7 cells (Fig. 8B). CerS4 knockdown attenuated the upregulation of N-cadherin, vimentin, and Snail in MCF-7/ADR cells and partially restored E-cadherin expression (Fig. 8C and D). Additionally, CerS4 knockdown inhibited cell migration in MCF-7/ADR cells (Fig. 8E). These data suggest a critical role for CerS4 in EMT progression in MDR breast cancer.

**Table 3:** Functional annotation of upregulated genes shared between MCF-7/CerS4 and MCF-7/ADR cells using MSigDB_Hallmark_2020.

MSigDB Hallmark 2020 term	Overlap	*P*-value	Adjusted *P*-value	Odds ratio	Combined score
Epithelial−Mesenchymal Transition	15 (200)	3.94E − 15	1.42E-13	23.52782194	780.3360494
TNF-α Signaling via NF-κB	14 (200)	9.13E − 14	1.64E-12	21.52360916	646.2376942
Hypoxia	10 (200)	1.03E − 08	1.24E-07	14.22278298	261.5745424
Complement	7 (200)	1.97E − 05	1.77E-04	9.412871557	102.0059035
Apical Junction	6 (200)	1.85E − 04	1.11E-03	7.921944035	68.10278008
Inflammatory Response	6 (200)	1.85E − 04	1.11E-03	7.921944035	68.10278008
IL-2/STAT5 Signaling	5 (199)	1.44E − 03	4.81E-03	6.516983875	42.65610088
mTORC1 Signaling	5 (200)	1.47E − 03	4.81E-03	6.483234714	42.29192897
E2F Targets	5 (200)	1.47E − 03	4.81E-03	6.483234714	42.29192897
p53 Pathway	5 (200)	1.47E − 03	4.81E-03	6.483234714	42.29192897

Functional annotation for the top 10 most significant of 83 upregulated genes (Fig. 8D) shared between MCF-7/CerS and MCF-7/ADR, obtained using Enrichr against the MSigDB_Hallmark_2022 library. IL: interleukin; mTORC1: mammalian target of rapamycin complex 1; NF-κB: nuclear factor kappa B; STAT5: signal transducer and activator of transcription 5; TNF-α: tumor necrosis factor-alpha

## Discussion

Although increased CerS4 expression in breast cancer has been reported [21, 22], the molecular mechanisms underlying CerS4 function and its roles in breast cancer development and progression remain incompletely understood. Since the concept of a sphingolipid rheostat was first suggested [39], ceramide has been regarded as a tumor suppressor that induces cell death and growth arrest [40]. However, recent studies suggest that endogenously generated ceramides have diverse functions that depend on their acyl chain lengths [41]. One study suggested opposing roles for long-chain and very-long-chain ceramides in breast cancer growth, and CerS4-overexpressing MCF-7 cells were reported to exhibit lower cell viability and decreased colony formation than control MCF-7 cells [24]. However, the previous study examining CerS4 overexpression used transient plasmid transfection [24], whereas the present study investigated the effects of long-term CerS4 overexpression on tumor progression and chemoresistance in MCF-7 cells. This approach revealed that persistent CerS4 overexpression in MCF-7 cells accelerated cell proliferation and MDR acquisition, suggesting that CerS4 may represent a potential therapeutic target in LumA breast cancer.

Multiple mechanisms could be involved in these processes. MCF-7/CerS4 cells exhibit increased expression of *SREBP-1c*, which plays an important role in cancer progression [11, 12]. *FASN* and *SCD1* are also considered therapeutic targets in many cancers [36, 37], and SREBPs regulate breast cancer cell invasion and migration [11, 12]. Furthermore, chronic CerS4 overexpression upregulated ABC transporters, such as *ABCC1*, *ABCC2*, *ABCC4*, *ABCB1*, and *ABCG2*. A major mechanism underlying MDR development is thought to be the overexpression of ABC transporters, which leads to the efflux of anticancer agents from tumor cells [42, 43]. In addition, increased ERα phosphorylation at Ser118 and Ser167, as observed in MCF-7/CerS4 cells, is linked to tamoxifen resistance [44, 45]. Chronic CerS4 overexpression in MCF-7 cells also altered several critical signaling pathways, including Akt/mTOR, NF-κB, and β-catenin, which play essential roles in cancer development and progression [4–6]. CerS4 overexpression in MCF-7 cells also increased cell cycle-related (Table 1) and cancer-related (Fig. 3; Tables 2 and 3) gene expression, suggesting that CerS4 overexpression could accelerate cell division and proliferation. Together, all of the alterations induced by stable CerS4 overexpression may contribute to the development of chemoresistance and cancer progression.

Although MCF-7/CerS4 cells exhibited reduced ERα expression, ERα phosphorylation increased at both Ser118 and Ser167. ERα phosphorylation is regulated by several upstream signaling pathways, such as Akt/mTOR and ERK/p90RSK [45]. Ser118 phosphorylation can be mediated by both epidermal growth factor (EGF) and insulin-like growth factor (IGF), whereas Ser167 phosphorylation only occurs in response to EGF stimulation [45]. In addition, oncogenic RET activation can induce ERα phosphorylation at Ser118 and Ser167 [46]. However, EGF receptor, IGF receptor, and RET expression levels were unchanged in MCF-7/CerS4 cells compared with control MCF-7 cells (Supplementary Figure S1), suggesting that ERα phosphorylation may be mediated by the Akt/mTOR or p90RSK signaling pathways in MCF-7/CerS4 cells. Although both MCF-7/CerS4 and MCF-7/ADR cells showed reduced ERα expression, CerS4 downregulation in MCF-7/ADR cells did not affect ERα expression, indicating that reduced ERα protein expression in MCF-7/ADR cells is not caused by CerS4 overexpression.

MCF-7/CerS4 and MCF-7/ADR cells share similar properties, such as drug resistance and high ABC transporter expression. However, the ABC transporter expression pattern in MCF-7/CerS4 cells differed from that in MCF-7/ADR cells. MCF-7/CerS4 cells displayed increased expression of *ABCC1*, *ABCC2*, *ABCC4*, *ABCB1*, and *ABCG2*, whereas MCF-7/ADR cells showed increased expression of *ABCC1*, *ABCC4*, and *ABCB1* but not *ABCC2* and *ABCG2*. CerS4 knockdown in MCF-7/ADR cells only reduced *ABCC1* and *ABCB1* expression, suggesting that CerS4 may positively regulate *ABCC1* and *ABCB1*, the most critical ABC transporters involved in the acquisition of chemoresistance. Consistently, CerS4 knockdown partially reversed the MDR phenotype in MCF-7/ADR cells upon treatment with doxorubicin, paclitaxel, or tamoxifen. These findings implicate CerS4 as an attractive target for overcoming chemoresistance in breast cancer, and treatment with various chemotherapeutic agents increased CerS4 expression (Supplementary Figure S2). The precise role of CerS4 in drug metabolism during chemotherapy remains to be further elucidated.

Stable CerS4 overexpression in MCF-7 cells also promotes EMT and cell migration, resulting in the upregulation of the mesenchymal markers N-cadherin, vimentin, and Snail [47] and the downregulation of the epithelial marker E-cadherin [47]. The loss of E-cadherin directly correlates with the loss of the epithelial phenotype [47]. Interestingly, CerS4 knockdown restored these phenotypes in MCF-7/ADR cells, indicating that CerS4 governs the EMT process in breast cancer and that reducing CerS4 expression may contribute to the shift of mesenchymal-like cancer cells toward an epithelial state. During the EMT, the tightly connected epithelial cells lose their polarity and gain a migratory mesenchymal phenotype [48]. Therefore, long-term CerS4 overexpression in breast cancer may also play essential roles in cancer cell migration and metastasis.

The present study introduced the possible oncogenic role of CerS4 in LumA breast cancer. CerS4 was found to be associated with many cancer-related signaling pathways, chemoresistance, and EMT. CerS4 overexpression also increased cell cycle and cell proliferation. Therefore, targeting CerS4 could serve as a novel therapeutic approach to inhibit tumor growth, enhance sensitivity to chemotherapy, and potentially prevent metastasis in LumA breast cancer patients. Analyses of CerS4 expression profiles could help optimize treatment plans and avoid ineffective therapies in breast cancer patients. However, further research and clinical studies will be necessary to fully validate the clinical relevance of CerS4 and its therapeutic potential.

## Conclusions

Long-term CerS4 overexpression promotes breast cancer progression and invasiveness by activating several cancer-related signaling pathways, such as Akt/mTOR, NF-κB, and β-catenin, and inducing EMT. In addition, CerS4 impacts chemoresistance via the positive regulation of MDR1 (*ABCB1*) and MRP1 (*ABCC1*), two key ABC transporters involved in MDR acquisition. Considering TCGA-BRCA data, which revealed that higher CerS4 expression was associated with poor prognosis in LumA, CerS4 might be applied as a potential prognostic or chemoresistance marker. Chronic alteration of CerS4 expression could critically impact breast cancer progression, metastasis and chemoresistance, positioning CerS4 as a novel target candidate for breast cancer therapy.

### Electronic supplementary material

Below is the link to the electronic supplementary material.

Supplementary Material 1

Supplementary Material 2

Supplementary Material 3

Supplementary Material 4

## Data Availability

The datasets used and/or analysed during the current study are available from the corresponding author on reasonable request.

## Abbreviations

ABC: ATP-binding cassette
AD: Adriamycin
Akt: protein kinase B
ANOVA: analysis of variance
BSA: bovine serum albutmin
CerS: ceramide synthase
DEG: differentially expressed gene
EGF: epidermal growth factor
EMT: epithelial−mesenchymal transition
ER: estrogen receptor
ERK: extracellular signal-regulated kinase
FASN: fatty acid synthase
FBS: fetal bovine serum
GEO: Gene Expression Omnibus
GSK: glycogen synthase kinase
HER2: human epidermal growth factor receptor 2
HMGCR: 3-hydroxy-3-methylglutaryl coenzyme A reductase
IC50: half maximal inhibitory concentration
IGF: insulin-like growth factor
LDLR: low-density lipoprotein receptor
LumA: luminal subtype A
LumB: luminal subtype B
MCF-7/ADR: Doxorubicin-resistant MCF-7 cell line
MCF-7/CerS4: CerS4-overexpressing MCF-7 stable cell line
MDR: multiple drug resistance
MRP: MDR-associated protein
mTOR: mammalian target of rapamycin
NF-κB: nuclear factor-kappa B
PGR: progesterone receptor
RNA-seq: RNA-sequencing
SCD1: stearoyl-coenzyme A desaturase 1
SREBP: sterol regulatory element-binding protein
S6K: p90 ribosomal S6 kinase
TCGA-BRCA: the Cancer Genome Atlas Breast Invasive Carcinoma
