# Supplementary material for: Ceramide synthase 4 overexpression exerts oncogenic properties in breast cancer
Source: Lipids Health Dis. 2023 Oct 26;22:183. doi: 10.1186/s12944-023-01930-z (PMC10605224; doi:10.1186/s12944-023-01930-z)
Supplement: Supplementary file 1 — Supplementary Material 1 [file 12944_2023_1930_MOESM1_ESM.pdf]

CerS4 paper final (edited).docx

As of: Aug 2, 2023 4:32:44 PM
8,065 words - 73 matches - 70 sources

Similarity Index

9%

Mode: Similarity Report

paper text:

Submitted to Lipids in Health and Disease Ceramide Synthase 4 Overexpression Exerts Oncogenic Properties in Breast Cancer Su-Jeong Kim1,†, Incheol Seo2,†, Min Hee Kim3, Joo-Won Park3, Shin Kim4,*, Woo-Jae Park1,* 1Department of Biochemistry, Chung-Ang University College of Medicine, Seoul 06974, Republic of Korea 2Department of Immunology, Kyungpook National University School of Medicine, Daegu 41944, Republic of Korea 3Department of Biochemistry, College of Medicine, Ewha Womans University, Seoul 07804, Republic of Korea 4Department of Immunology, School of Medicine, Keimyung University, Daegu 42601, Republic of Korea †These authors contributed equally to this work. *Corresponding authors: Woo-Jae Park, Department of Biochemistry, Chung-Ang University College of Medicine, Heukseok-lo 84, DongJak-gu, Seoul 06974, Republic of Korea. Tel.: +82- 2-820-5308; E-mail: ooze@cau.ac.kr; Shin Kim, Department of Immunology, School of Medicine, Keimyung University, Dalgubeol-daero 1095, Dalseo-gu, Daegu 42601, Republic of Korea. Tel.: +82-53-258-7359; Fax: +82-53-258-7355; E-mail: god98005@dsmc.or.kr Declarations of interest: none Abstract Background: Ceramide, a bioactive signaling sphingolipid,

has long been implicated in cancer . Members of **the ceramide** 25

synthase (CerS) family determine the acyl chain lengths of ceramides, with ceramide synthase 4 (CerS4) primarily generating C18–C20-ceramide. Although CerS4 is known to be overexpressed

in breast cancer, its role in breast cancer 6

pathogenesis is not well established. Methods: To investigate the role of CerS4 in breast cancer, public datasets, including The Cancer Genome Atlas (TCGA) and two Gene Expression Omnibus (GEO) datasets (GSE115577 and GSE96058) were analyzed. Furthermore, MCF-7 cells stably overexpressing CerS4 (MCF-7/CerS4) as a model for luminal subtype A (LumA) breast cancer were produced, and doxorubicin (also known as Adriamycin [AD])-resistant MCF-7/ADR cells were generated after prolonged treatment of MCF-7 cells with doxorubicin. Kaplan–Meier survival analysis assessed the clinical significance of CERS4 expression, while Student's t-tests or Analysis of Variance (ANOVA) compared gene expression and cell viability in different MCF-7 cell lines. Results: Analysis of the public datasets revealed elevated CERS4 expression in breast cancer, especially in the most common breast cancer subtype, LumA. Persistent CerS4 overexpression in MCF-7 cells activated multiple cancer-associated pathways, including pathways involving

sterol regulatory element–binding protein, nuclear factor kappa B 48

(NF- κB), Akt/mammalian target of rapamycin (mTOR), and β-catenin. Furthermore, MCF- 7/CerS4 cells acquired doxorubicin, paclitaxel, and tamoxifen resistance, with concomitant upregulation of ATP-binding cassette (ABC) transporter genes, such as ABCB1, ABCC1, ABCC2, ABCC4, and ABCG2. MCF-7/CerS4 cells were characterized by increased cell migration and epithelial–mesenchymal transition (EMT). Finally, CERS4 knockdown in doxorubicin-resistant MCF-7/ADR cells resulted in reduced activation of cancer-associated 2 pathways (NF-κB, Akt/mTOR, β-catenin, and EMT) and diminished chemoresistance, accompanied by ABCB1 and ABCC1 downregulation. Conclusions: Chronic CerS4 overexpression may exert oncogenic effects in breast cancer via alterations in signaling, EMT, and chemoresistance. Therefore, CerS4 may represent an attractive target for anticancer therapy, especially in LumA breast cancer. Key words: ceramide synthase 4, ceramide acyl chain length, oncogene, epithelial mesenchymal transition, chemoresistance, Luminal subtype A breast cancer Background

Breast cancer is one of the most common malignancies worldwide 41

, associated with high mortality among women [1].

Breast cancer can be classified **based on the** expression status **of** 57

estrogen receptor (ER), progesterone receptor (PGR), human epidermal growth factor receptor 2 (HER2), and Ki67 [2]. Molecular breast cancer subtypes include normal-like (ER+, PGR+, HER2−, Ki67−), basal (ER−, PGR−, HER2−, basal marker+), luminal subtype A (LumA; ER+, PGR+, HER2−, Ki67−), luminal subtype B (LumB; ER+, PGR+, HER2−, Ki67+), HER2-positive (ER−, PGR−, HER2+), and triple-negative (ER−, PGR−, HER2−) [2]. Accounting for 50%–60% of all breast cancer diagnoses [3], LumA is the most prevalent subtype and is associated with relatively good clinical outcomes and prognosis; however, a mechanistic understanding of LumA pathogenesis and the development of novel chemotherapies remain critical. Various cancer

pathways, such as nuclear factor kappa B (NF-κB), protein kinase B (Akt 32

)/mammalian target of rapamycin (mTOR), and Wnt/β-catenin, play crucial roles in breast cancer pathogenesis [4-6]. Specifically, NF-κB activation, which is frequently observed

in breast cancer , promotes **the development of a hormone-independent** and **invasive** 40

3 breast cancer phenotype [7], whereas its inhibition reverses endocrine resistance [4]. Activation of the Akt/mTOR pathway, which regulates various cellular activities, including protein synthesis, metabolic regulation, cell survival, and differentiation [8], facilitates tumor growth and breast cancer cell survival [9]. Activation of the Wnt/β-catenin pathway affects breast cancer cell migration and metastasis [6].

Sterol regulatory element–binding proteins (SREBPs) are transcription factors that regulate fatty acid and cholesterol synthesis 23

[10] and are known to regulate breast cancer cell invasion and migration [11, 12]. Metastasis and chemotherapy resistance are leading causes of death among breast cancer patients. The epithelial–mesenchymal transition (EMT), during which polarized epithelial cells transition into motile mesenchymal cells, allows breast tumors to become more invasive and malignant, with enhanced stem cell properties [13]. In addition, the overexpression of ATP-binding cassette (ABC) transporters confers chemotherapy resistance by expelling various drugs from tumor cells at the expense of ATP hydrolysis and contributes to breast cancer progression and metastasis through mechanisms independent of this efflux function [14]. All of these pathways and molecular mechanisms are potential therapeutic targets in breast cancer. Sphingolipids are implicated in several pathophysiological processes associated with cancer development, progression, metastasis, and drug resistance [15]. Ceramide, located at the center of sphingolipid metabolism, acts as a biologically active lipid that determines cell fate decisions, including apoptosis, proliferation, and differentiation [16, 17]. Recent reports describe distinct roles played by ceramide species with different acyl chain lengths in various molecular pathways, including endoplasmic reticulum stress [18] and apoptosis [19]. In mammals, the acyl chain lengths of ceramides are determined by six ceramide synthase (CerS) family members [16, 17]. CerS1 and CerS2 generate C18-ceramide and C22–C24- ceramide, respectively. CerS4 primarily synthesizes C18-C20-ceramide, whereas CerS5 and 4 CerS6 primarily produce C14-C16-ceramide [20]. In human breast cancer, the mRNA expression levels of CERS2, CERS4, and CERS6 and their respective products, C16-, C24-, and C24:1-ceramides, are increased [21, 22]. In addition, alternative splicing of CerS2 in LumB breast cancer promotes cancer cell proliferation and migration and is associated with poor prognosis [23]. Thus, alterations in CerS activity or ceramide acyl chain lengths may play critical roles in breast cancer development and progression. Unlike prior studies that have investigated the roles played by CerS family members in breast cancer using transient overexpression [24, 25], in the present study, the molecular effects of long-term CerS4 overexpression on breast cancer progression and migration were explored using TCGA-BRCA data, MCF-7 cells stably overexpressing CerS4 (MCF- 7/CerS4), and doxorubicin-resistant MCF-7 (MCF-7/ADR) cells. Methods Materials The following substances were obtained: doxorubicin, paclitaxel, G418, anti-HA (H6908), anti-CerS2 (HPA027262), anti-CerS4 (SAB4301210), anti-active-?-catenin (05-665), and

anti-α-tubulin antibodies (**T9026) (Sigma-Aldrich, St. Louis, MO, USA); anti** -phospho- **NF-κB p65** 2

(3033), anti-NF-κB p65 (8242), anti-Relb (4922), anti-phospho-p44/42 MAPK (ERK1/2) (4370), anti-phospho-p90RSK (11989), anti-phospho-Akt (9271), anti-phospho- mTOR (5536), anti-phospho-p70 S6 kinase (9205), anti-ERα (8644), anti-

phospho-ERα (Ser118) (2511), anti-phospho-ERα (Ser167) (64508), anti-PGR (8757), anti-vimentin (5741), anti-Snail (3879), anti-lamin A/C (2032), ?-catenin (9562), and anti-phospho-GSK3? (5558) antibodies (Cell Signaling Biotechnology, Inc, Beverly, MA, USA); anti-LASS6 (CerS6) (sc- 100554), anti-E-cadherin (sc-71008), and anti-N-cadherin (sc-59987) antibodies (Santa Cruz Biotechnology, Santa Cruz, CA, USA); anti-CerS1 antibody (H00010715-M01) (Abnova, 5 Taipei, Taiwan); anti-GAPDH (glyceraldehyde 3-phosphate dehydrogenase) antibody (MAB374) (EMD Millipore, Billerica, MA, USA). Cell culture MCF-7 cells (RRID:CVCL_0031), a human breast cancer cell line, were

cultured in RPMI-1640 medium (Hyclone Laboratories, Logan, UT, USA 4

) containing 1% penicillin/streptomycin and

10% fetal bovine serum (FBS, Hyclone Laboratories). The cells were incubated at 37℃ in a humidified atmosphere with **5% CO2** 18

. MCF-7/CerS4 cells

were cultured in RPMI-1640 medium, 1% penicillin/streptomycin, 10 % FBS (Hyclone Laboratories), **and** 35

100 μg/ml G418 (Sigma-Aldrich). Transfection and stable cell line generation The

MCF-7 cells were transfected with 4 **μg of the** 44

pcDNA3.1-CerS4-HA, using 150 mM NaCl and Lipidofect-P Transfection Reagent (Lipidomia, Seongnam, Korea). Thereafter, G418 was used to create permanently stable transfected cell lines. At first, 300 μg/ml of G418 was strongly treated for selection, and the surviving cells were

maintained in fresh **RPMI-1640 medium containing 100 μg/ml** of **G418** 9

. G418 was removed 48 h before experiments. The MCF-7/ADR

cells were transfected with 3 **μg of the pSUPER** 56

-shCerS4. Transfected vectors such as pcDNA3.1-CerS4-HA and pSUPER-shCerS4 were provided from Professor A.H. Futerman (Weizmann Institute of Science, Rehovot, Israel). Generation of doxorubicin-resistant cell lines MCF-7/ADR cells were generated by prolonged treatment with doxorubicin at increasing concentrations (0.5~25 μM) for 6-8 months, as previously described [26]. Western blot analysis The MCF-7, MCF-7/CerS4, and MCF-7/ADR

cells were lysed by **RIPA buffer (50 mM Tris** -Cl; **pH 7.5, 150 mM NaCl, 0.1** % sodium dodecyl sulfate [**SDS], 0.5% sodium deoxycholate, 1% Triton X-100** 13

or

Nonidet P-40 (NP-40), protease inhibitors, **and** 69

phosphatase inhibitors). Cell lysates

were incubated on ice for 30 min, and 58

then

the supernatants were obtained by **centrifugation at 12,000 ×g for 15 min at 4** 30

℃. Quantification of the lysed

protein was measured with Bio-Rad Protein Assay Dye Reagent (Bio-Rad Laboratories, Hercules, CA 26

, USA). Afterward, 30~50 μg of the heat-denatured protein was loaded and

separated by 10% **SDS-PAGE. Separated proteins were transferred to a nitrocellulose membrane (Bio-Rad** 29

Laboratories), which was further blocked by

5% bovine serum albumin (BSA, Sigma-Aldrich) in TBST (**TBS** 63

with

0.1% Tween 20) for 1 h. The membrane was incubated on **a** shaking incubator **at** 8

4℃ overnight to attach the primary antibody. Then, after washing with TBST,

the secondary antibody was attached **at room temperature for 1 h** . Both primary and **secondary antibodies were** 16

dissolved in TBST. Blots on the membrane were detected using ECL western blotting detection solution (ATTO), and then a digitized image of the membrane was captured using the ChemiDoc MP imaging system (Bio-Rad Laboratories). RNA isolation and reverse transcription-real-time PCR Total mRNA of MCF-7 cells and MCF-7/CerS4 cells was extracted using RNeasy Mini Kits (Qiagen, Valencia, CA, USA), and mRNA was quantified using the NanoDrop 1000 spectrophotometer (Thermo Scientific, Wilmington, Denmark). To synthesize cDNA from mRNA, the Verso cDNA Synthesis Kit (Thermo Scientific) was used. To

perform real-time PCR, a total 70

volume of 10 **μl containing 0.1 μg cDNA, 10 pmol** forward **primer** 36

and reverse primer, and SYBR-Green Master Mix (Thunderbird) was used. The analysis was performed using the CFX Connect Real-time PCR Detection System (Bio-Rad Laboratories). Thermal 7 cycling conditions consisted of 1

min at 95℃, followed by 40 cycles of 15 s at 95℃ and 45 s **at 60℃. The** 1

primers used in this paper are summarized in Supplementary Table S1.

MTT assay Viabilities **of MCF-7 cells and** 64

MCF-7/CerS4 cells were

measured by 3-(4,5- dimethylthiazol-2-yl)-2,5-diphenyltetrazolium bromide (MTT) assay . The **cells were seeded** at 5×104 **in 96-well plates** 11

. After the cells had adhered, doxorubicin, paclitaxel, and tamoxifen were treated at different concentrations (0~50 μM) for 2 days. Then,

MTT solution (5 mg/ml in phosphate-buffered saline [PBS]) was added to each well and incubated at 37℃ **for 2** -3 **h** 15

until a purple precipitate was visible. Finally, 10% SDS was added to each well, and

the absorbance was measured at 570 nm . Transwell invasion assay **with** Matrigel **A** 49

transparent PET membrane insert with an 8.0 μm hole was inserted into a 24-well plate. For coating of inserts, Matrigel and FBS-free medium were mixed in a ratio of 1:6. Then, 40-50 μl of the mixture was placed on the insert and incubated overnight in a 37℃ incubator for drying. After incubation, 750 μl of medium with 10% FBS was placed in the bottom well, and 5×10^4 cells in 200 μl of medium containing 0.5% FBS were added to the insert. After incubation for 48 h to allow cells to penetrate the lower wells, the cells were fixed using 100% methanol and

stained with 0.4% trypan blue solution (Sigma-Aldrich 53

) for 10 min. After washing with PBS, cell invasion was captured under a microscope. LC-ESI-MS/MS analysis of ceramides Ceramide analyses by LC-ESI-MS/MS were conducted as described previously [27]. RNA-sequencing (RNA-Seq) analysis Gene expression profiles of MCF-7 and MCF-7/CerS4 cells were compared using RNA-Seq. Total mRNA

was extracted using RNeasy Mini Kits (Qiagen) according to the manufacturer's protocol. The 28

raw sequencing data were generated by the Illumina NovaSeq platform (pair-end, 2×101 bp).

FastQC was used to assess the quality of the raw sequencing reads 33

. Then, sequencing

reads were aligned to the GRCh38 **reference genome using the STAR aligner** 34

in two-pass mode with default parameters [28]. DESeq2 analysis was performed to identify differentially expressed genes (DEGs). Statistically significant DEGs were defined with adjusted P < 0.0001 and |log2FC| > 1.5. Analysis of TCGA-BRCA and GEO dataset To compare CERS4 expression between normal and cancer or LumA and non-luminal subtype A (non-LumA) breast cancer, TCGA-BRCA, GSE115577, and GSE96058 data were used. STAR raw count data for TCGA-BRCA were obtained in R using the TCGAbiolinks package. Then, the raw count was normalized using DESeq2 [29]. The RMA-normalized probe intensities were obtained for GSE115577 in R using the GEOquery package [30]. The FPKM expression matrix for GSE96058 was obtained from GEO. The molecular subtypes were obtained from metadata in each dataset [31]. Kaplan?Meier survival analysis was performed to investigate the relationship between CERS4 expression and overall survival of the patient with LumA breast cancer. The optimal cut-off value for survival analysis was determined using the survminer package in R. GSE116436 was used to investigate changes in CERS4 expression in MCF-7 cells after various treatments with a chemotherapeutic agent. The gene expression profiles and EMT score of doxorubicin-resistant MCF-7/ADR cells were investigated using MCF-7/ADR dataset (GSE24460). The RMA-normalized probe intensities were obtained, and the limma package was used in R for DEG analysis [32]. Statistically significant DEGs were defined with adjusted P < 0.01 and |log2FC| > 1. Bioinformatic and statistical analyses All experiments were independently repeated three times, and the values were

expressed as the **mean ? standard error of the mean** 62

. Half maximal inhibitory concentration (IC50) for doxorubicin, paclitaxel, and tamoxifen was calculated using the drm package in R [33]. The two-tailed Student t-test was performed to compare individual gene expression or cell viability between different MCF-7 cell lines with P < 0.05. For comparisons involving more than three cell lines, multiple time points, or treatment dosages, one- or two-way Analysis of Variance (ANOVA), and a Tukey post hoc test were performed. The functional annotation of DEGs was performed using Enrichr [34].

The EMT score was calculated from 66

RNA-Seq or microarray

by the sum of the **expression of** nine **well-known mesenchymal marker genes minus the total expression of** five **known epithelial genes** 19

, as previously described [35]. Bioinformatic and statistical

analyses were performed using R version 4.2.1. Results Expression of CERS4 **and** 37

survival analysis across breast cancer cohorts Although elevated mRNA levels have been reported for CERS2, CERS4, and CERS6 in human breast cancer [21, 22], the present study specifically focused on CerS4 because this protein was upregulated in not only TCGA-BRCA data but also MCF-7/ADR cells among all CerS proteins, indicating that CerS4 is expected to play an important role in breast cancer. To explore whether CerS4 expression is altered in breast cancer, The Cancer Genome Atlas Breast Invasive Carcinoma (TCGA-BRCA) and two Gene Expression Omnibus (GEO) datasets (GSE115577 and GSE96058) were analyzed. These analyses revealed significantly 10 higher CERS4 mRNA expression levels in tumor tissues than in normal tissues in two cohorts (TCGA-BRCA, $P < 0.0001$; GSE115577, $P < 0.0001$; Figure 1A). To determine which breast cancer subtypes are characterized by elevated CERS4 mRNA, the datasets were reanalyzed according to breast cancer subtype, revealing significantly higher CERS4 mRNA expression in LumA than in non-LumA (LumB, HER2-positive, and basal) subtypes across all three cohorts (TCGA-BRCA,

$P < 0.0001$; GSE115577, **$P = 0$** .007; GSE96058, **$P < 0.0001$** 60

; Figure 1B). To elucidate the relationship between CERS4 expression and clinical prognosis in LumA breast cancer, Kaplan–Meier survival analyses were performed comparing LumA patients with higher CERS4 expression and those with lower CERS4 expression based on an optimal cutoff value. This analysis revealed that higher CERS4 mRNA expression levels were associated with poor overall survival in LumA breast cancer (TCGA-BRCA, $P = 0.048$; GSE96058, $P = 0.0014$; Figure 1C). Identification of the molecular factors involved in cell proliferation in CerS4- overexpressing MCF-7 cells To explore the effects of chronic CerS4 overexpression, CerS4 was stably overexpressed in MCF-7 cells (MCF-7/CerS4), which express ER and PGR and are considered representative of the LumA [36]. CERS4 mRNA levels increased by approximately 120-fold, and CerS4 protein levels increased by approximately 2.3-fold in MCF-7/CerS4 cells compared with control MCF-7 cells, with no apparent alterations in the mRNA or protein levels of other CerS family members (Figure 2A and C). CerS4 overexpression also increased the levels of C18- and C20-ceramides without changes in other ceramides (Figure 2B). To examine the effect of persistent CerS4 overexpression on cell proliferation, cell growth

was measured using the MTT cell viability assay 24

. MCF-7/CerS4 cell proliferation was greater than that for control MCF-7 cells (Figure 2D), and functional 278 annotation of upregulated DEGs in MCF-7/CerS4 cells compared with control MCF-7 cells 279 further showed activated cell cycle pathways (Table 1). These results suggest that CerS4 280 contributes to tumor cell proliferation by promoting cell cycle transitions. 281 282 Table 1. Functional annotation of upregulated genes in MCF-7/CerS4 cells using 283 Reactome_2022. 284 285 286 287 288 289 290 291 292 293 294 Innate Immune System R-HSA-168249 Immune System R-HSA-168256 Cell Cycle, Mitotic R-HSA-69278

Mitotic G1 Phase and G1/S Transition R- HSA-453279 43

Cell Cycle

R-HSA -1640170 **Interleukin-4 and Interleukin-13 Signaling R-HSA-6785807** G1/S Transition 14
R-HSA

-69206 G1/S-Specific Transcription

R-HSA -69205 **NGF-Stimulated Transcription R-HSA- 9031628** 14

100 (1035) 155 (1943) 60 (523) 28 (147) 68 (654) 23 (107) 25 (129) 12 (29) 13 (39) 1.80E−12 7.02E−12 9.03E−11 1.55E−10 3.77E−10 5.57E−10 1.02E−09 2.29E−09 1.11E−08 2.36E-09 4.60E-09 3.94E-08 5.09E-08 9.87E-08 1.22E-07 1.90E-07 3.75E-07 1.35E-06 2.34569897 1.940958578 2.781618301 4.960806124 2.497379682 5.751514433 5.055272471 14.70086789 10.41958425 63.43460249 49.846957 64.33409133 112.0443945 54.19260145 122.5517755 104.6787947 292.4689634 190.8686062 Functional annotation for the top 10 most significant of 927 differentially upregulated genes in MCF-7/CerS4, obtained using Enrichr against the Reactome_2022 library. To identify the molecular mechanisms involved in the increased proliferation observed for MCF-7/CerS4 cells compared with control MCF-7 cells, several proliferation- related signaling pathways were examined. NF-κB and Akt/mTOR activation and increased p90 ribosomal S6 kinase (p90RSK) phosphorylation were observed in MCF-7/CerS4 cells compared with control MCF-7 cells, whereas extracellular signal-regulated kinase (ERK) phosphorylation was reduced (Figure 3A), implying that p90RSK activation occurred independent of ERK activation. In MCF-7/CerS4 cells, glycogen synthase kinase-3β (GSK3β) phosphorylation was accompanied by β-catenin activation (Figure 3A). In MCF- 7/CerS4 cells, β-catenin levels in both the nucleus and the cytosol increased compared with levels in control MCF-7 cells (Figure 3B), revealing β-catenin pathway activation in the presence of persistent CerS4 overexpression. Because MCF-7 cells are positive for both ER and PGR [37], the potential alterations in the expression of certain hormone receptors, including ERα (ESR1), ERβ (ESR2), and PGR (PGR), which are clinically important for predicting endocrine therapy efficacy, following persistent CerS4 overexpression were evaluated. ESR1 mRNA levels were significantly reduced in MCF-7/CerS4 cells compared with control MCF-7 cells, whereas the expression levels of other genes, such as ESR2 and PGR, were not altered (Figure 3C). Consistent with lower ESR1 mRNA levels, ERα protein levels were also reduced in MCF-7/CerS4 cells compared with control MCF-7 cells, and ERα phosphorylation increased at both Ser118 and Ser167 (Figure 3D and E). Increased PGR protein expression was observed in MCF-7/CerS4 cells compared with control MCF-7 cells, despite unaltered PGR mRNA levels (Figure 3C, D and E). SREBPs regulate fatty acid and cholesterol metabolism [10], and their targets genes, such as

fatty acid synthase (FASN) and stearoyl-CoA desaturase -1 (SCD1), **are** therapeutic targets **in** 42

many cancers [38, 39]. Therefore, the potential effects of persistent CerS4 overexpression on the expression of SREBPs and their downstream targets, such as FASN, SCD1, β-hydroxy-β-methylglutaryl coenzyme A (HMG-CoA) reductase (HMGCR), and low- density lipoprotein receptor (LDLR), were examined. The mRNA expression levels of SREBP-1c and SREBP-2 increased in MCF-7/CerS4 cells compared with control MCF-7 cells, accompanied by the upregulation of FASN, SCD, HMGCR, and LDLR (Figure 3F). RNA-Seq analysis revealed that upregulated genes in MCF-7/CerS4 cells compared with control MCF-7 cells included well-known cancer-related transcription factors, such as ERG, 319 320 321 322 323 MYC, ESR1, TP53, SP1, EP300, RAD21, NFKB1, SMAD3, and HDAC2 (Table 2).

These data suggest a potential **role for** 68

CerS4 in breast cancer progression via transcriptional regulation. Table 2. Functional annotation of upregulated genes in MCF-7/CerS4 cells using Transcription_Factor_PPIs. Transcription_ Factor_PPIs term Overlap P-value Adjusted P-value Odds ratio Combined score ERG 17 (41) 9.65E-13 2.39E−10 14.82751832 410.2284253 MYC 93 (957) 8.40E-12 8.14E−10 2.350115741 59.93357056 ESR1 87 (871) 9.85E-12 8.14E−10 2.416094206 61.23323517 TP53 65 (628) 1.12E-09 6.97E−08 2.47915748 51.08547827 SP1 37 (263) 1.68E-09 8.35E−08 3.466933479 70.04192295 EP300 51 (473) 1.93E-08 7.97E−07 2.573094527 45.70841512 RAD21 32 (237) 5.67E-08 2.01E−06 3.29078076 54.90623716 NFKB1 33 (254) 8.98E-08 2.78E−06 3.148774636 51.09018537 SMAD3 41 (375) 3.30E-07 9.09E−06 2.59627134 38.74730225 HDAC2 40 (387) 1.92E-06 4.77E−05 2.433615236 32.03028341 324 325 326 327 328 Functional annotation for the top 10 most significant of 927 differentially upregulated genes in MCF-7/CerS), obtained using Enrichr against the Transcription_Factor_PPIs library Multidrug-resistance (MDR) properties of CerS4-overexpressing MCF-7 cells Because chemotherapy failure can lead to cancer recurrence and death, the role of 329 persistent CerS4 overexpression in the alteration of the chemoresistance properties of MCF-7 330 cells was examined by evaluating the impact of CerS4 overexpression on the responses of 331 MCF-7 cells to the anticancer drugs doxorubicin, paclitaxel, and tamoxifen. Increased 332 resistance to these three drugs in MCF-7/CerS4 cells compared with control MCF-7 cells was 333 detected (Figure 4A–C). To identify the molecular mechanisms involved in CerS4-induced 334 chemoresistance, microarray data were analyzed to examine the expression profiles of 335 various ABC transporter genes.

As shown in Figure 4D, **the** mRNA **expression levels of** 22

ABCA3, ABCA12, TAP2, ABCC1, ABCC2, ABCD1, ABCD3, ABCE1, ABCF1, ABCF2, and 14 ABCG2 were significantly higher in MCF-7/CerS4 cells than in control MCF-7 cells, whereas the levels of ABCA2, ABCA5, ABCA7, ABCB6, ABCB9, ABCC5, ABCD4, and ABCG1 were significantly reduced. Three groups of ABC transporters play major roles in chemoresistance:

the classical P-glycoproteins (MDR1, ABCB1), the MDR-associated proteins (MRPs, in the ABCC subfamily), and ABCG2 (an ABC half-transporter) [40]. These gene expression results were then confirmed using real-time PCR. Consistent with increased chemoresistance following CerS4 overexpression (Figure 4A–C), ABCB1 (MDR1), ABCC1 (

MRP1), ABCC2 (MRP2), ABCC4 (MRP4), and **ABCG2 (breast cancer resistance protein** 20

) were upregulated in MCF-7/CerS4 cells compared with control MCF-7 cells, whereas ABCB4 (MDR3) and ABCC11 (MRP8) were downregulated (Figure 4E). Cell migration and EMT properties in CerS4-overexpressing MCF-7 cells The effect of CerS4 overexpression on the EMT process was analyzed in MCF-7 cells. The levels of E-cadherin, an epithelial marker, were reduced in MCF-7/CerS4 cells compared with control MCF-7 cells, whereas the

levels of mesenchymal cell markers , such as **N-cadherin, vimentin, and Snail** 22

, increased (Figure 5A). The EMT score was significantly

higher in MCF-7 /CerS4 cells **than in** control MCF-7 **cells** (Figure **5B** 54

). In addition, cell migration increased in MCF-7/CerS4 cells compared with control MCF-7 cells (Figure 5C). These data suggest that CerS4 overexpression causes MCF-7 cells to acquire the characteristics of migratory mesenchymal cells. Impacts of CerS4 in overcoming MDR in MCF-7 cells To test the potential that CerS4 may serve

as a therapeutic target for overcoming MDR **in breast cancer** 52

, CerS expression in Adriamycin (AD, also known as doxorubicin)- resistant MCF-7/ADR cells was analyzed. CERS2 mRNA expression was reduced, whereas 15 CERS4 mRNA expression was significantly upregulated in MCF-7/ADR cells compared with control MCF-7 cells (Figure 6A). Though C16- and C20-ceramide levels were elevated in MCF-7/ADR cells, C22-, C24-, C24:1-ceramide levels were reduced (Figure 6B). To elucidate whether CerS4

plays a role in the acquisition **of** chemoresistance, **MCF-7** /ADR **cells** 45

were transfected with plasmids expressing CerS4-targeting shRNA (shCerS4). Reduced CERS4 expression and C20-ceramide level following shCerS4 plasmid transfection were confirmed in MCF-7/ADR cells (Figure 6A and B). CerS4 knockdown partially reversed drug resistance in MCF-7/ADR cells treated with doxorubicin, paclitaxel, or tamoxifen (Figure 6C–E), implying a critical role for CerS4 in the acquisition of MDR in breast cancer. To further elucidate the

molecular mechanisms through which CerS4 knockdown overcomes breast cancer chemoresistance, various signaling pathways associated with cell proliferation were examined. As shown in Figure 7A, the NF-κB, Akt/mTOR, and β-catenin pathways were activated in MCF-7/ADR cells, and this activation was suppressed by CerS4 knockdown. However, downregulated ERα expression in MCF-7/ADR cells was not recovered by CerS4 knockdown. To explore whether CerS4

plays a role in the regulation of ABC transporter **gene expression** 47

, microarray data were analyzed. ABCA3, ABCB1 (209993_at and 209994_s_at), ABCB8, TAP1 (202307_s_at), TAP2 (204770_at), ABCC1 (202804_at and 202805_s_at), ABCC4, ABCC6 (214033_at), ABCE1 (201872_s_at), ABCF2 (209247_s_at), and ABCF3 (202394_s_at) were significantly upregulated in MCF-7/ADR cells compared with control MCF-7 cells, whereas ABCA5, ABCA12, ABCC3 (209641_s_at), ABCG1 (204567_s_at), and ABCG2 were downregulated (Figure 7B). CerS4 knockdown partially attenuated the upregulation of ABCB1 and ABCC1 and restored ABCC2 expression in MCF-7/ADR cells (Figure 7C). These data suggest that CerS4 overexpression may overcome chemoresistance by modulating gene expression associated with proliferation- related signaling pathways and ABC transporters, such as MDR1 and MRP1. 16 To investigate whether targeting CerS4 hampers EMT progression in an MDR breast cancer cell model, microarray data were analyzed. A total of 83 upregulated and 13 downregulated DEGs were shared between MCF-7/CerS4 and MCF-7/ADR cells (GSE24460; Figure 8A, Supplementary Table S2). Functional annotation of the 83 shared, upregulated DEGs revealed the upregulation of the EMT pathway in both MCF-7/CerS4 and MCF-7/ADR cells (Table 3). Similarly, the EMT score calculated using the MCF-7/ADR dataset (GSE24460) was higher for MCF-7/ADR cells than for control MCF-7 cells (Figure 8B). CerS4 knockdown attenuated the upregulation of N-cadherin, vimentin, and Snail in MCF-7/ADR cells and partially restored E-cadherin expression (Figure 8C and D). Additionally, CerS4 knockdown inhibited cell migration in MCF-7/ADR cells (Figure 8E). These data suggest a critical role for CerS4 in EMT progression in MDR breast cancer. Table 3. Functional annotation of upregulated genes shared between MCF-7/CerS4 and MCF-7/ADR cells using MSigDB_Hallmark_2020. MSigDB Hallmark 2020 Overlap P-value Adjusted term P-value Odds ratio Combined score Epithelial?Mesenchymal Transition 15 (200) 3.94E−15 1.42E-13 23.52782194 780.3360494 TNF-α Signaling via NF- κB 14 (200) 9.13E−14 1.64E-12 21.52360916 646.2376942 Hypoxia 10 (200) 1.03E−08 1.24E-07 14.22278298 261.5745424 Complement 7 (200) 1.97E−05 1.77E-04 9.412871557 102.0059035 Apical Junction 6 (200) 1.85E−04 1.11E-03 7.921944035 68.10278008 Inflammatory Response 6 (200) 1.85E−04 1.11E-03 7.921944035 68.10278008 IL-2/STAT5 Signaling 5 (199) 1.44E−03 4.81E-03 6.516983875 42.65610088 mTORC1 Signaling 5 (200) 1.47E−03 4.81E-03 6.483234714 42.29192897 E2F Targets 5 (200) 1.47E−03 4.81E-03 6.483234714 42.29192897 p53 Pathway 5 (200) 1.47E−03 4.81E-03 6.483234714 42.29192897 401 Functional annotation for the top 10 most significant of 83 upregulated genes (Figure 8D) 402 shared between MCF-7/CerS and MCF-7/ADR, obtained using Enrichr against the MSigDB_Hallmark_2022 library. IL, interleukin; mTORC1, mammalian target of rapamycin 17 404 complex

1; NF-κB, nuclear factor kappa B ; STAT5, **signal transducer and activator of** 405 **transcription** 31

5;

TNF-α, tumor necrosis factor-alpha	67

. Discussion Although increased CerS4 expression in breast cancer has been reported [21, 22], the molecular mechanisms underlying CerS4 function and its roles in breast cancer development and progression remain incompletely understood. Since the concept of a sphingolipid rheostat was first suggested [41], ceramide has been regarded as a tumor suppressor that induces cell death and growth arrest [42]. However, recent studies suggest that endogenously generated ceramides have diverse functions that depend on their acyl chain lengths [43]. One study suggested opposing roles for long-chain and very-long-chain ceramides in breast cancer growth, and CerS4-overexpressing MCF-7 cells were reported to exhibit lower cell viability and decreased colony formation than control MCF-7 cells [44]. However, the previous study examining CerS4 overexpression used transient plasmid transfection [44], whereas the present study investigated the effects of long-term CerS4 overexpression on tumor progression and chemoresistance in MCF-7 cells. This approach revealed that persistent CerS4 overexpression in MCF-7 cells accelerated cell proliferation and MDR acquisition, suggesting that CerS4 may represent a potential therapeutic target in LumA breast cancer. Multiple mechanisms could be involved in these processes. MCF-7/CerS4 cells exhibit increased expression of SREBP-1c, which plays an important role in cancer progression [11, 12]. FASN and SCD1 are also considered therapeutic targets in many cancers [38, 39], and SREBPs regulate breast cancer cell invasion and migration [11, 12]. Furthermore, chronic CerS4 overexpression upregulated ABC transporters, such as ABCC1, ABCC2, ABCC4, ABCB1, and ABCG2. A major mechanism underlying MDR development is thought to be the overexpression of ABC transporters, which leads to the efflux of anticancer agents from tumor cells [45, 46]. In addition, increased ERα phosphorylation at Ser118 and Ser167, as observed in MCF-7/CerS4 cells, is linked to tamoxifen resistance [47]. Chronic CerS4 overexpression in MCF-7 cells also altered several critical signaling pathways, 19 including Akt/mTOR, NF-κB, and β-catenin, which play essential roles in cancer development and progression [4-6]. CerS4 overexpression in MCF-7 cells also increased cell cycle-related (Table 1) and cancer-related (Figure 3, Tables 2 and 3) gene expression, suggesting that CerS4 overexpression could accelerate cell division and proliferation. Together, all of the alterations induced by stable CerS4 overexpression may contribute to the development of chemoresistance and cancer progression. Although MCF-7/CerS4 cells exhibited reduced ERα expression, ERα phosphorylation increased at both Ser118 and Ser167. ERα phosphorylation is regulated by several upstream signaling pathways, such as Akt/mTOR and ERK/p90RSK [48]. Ser118 phosphorylation can be

**mediated by** both **epidermal growth factor (EGF**) and **insulin-like growth factor (IGF**	38

), whereas Ser167 phosphorylation only occurs in response to EGF stimulation [48]. In addition, oncogenic RET activation can induce ERα phosphorylation at Ser118 and Ser167 [49]. However, EGF receptor, IGF receptor, and RET expression levels were unchanged in MCF-7/CerS4 cells compared with control MCF-7 cells (Supplementary Figure S1), suggesting that ERα phosphorylation

may be mediated by the Akt/mTOR 65

or p90RSK signaling pathways in MCF-7/CerS4 cells. Although both MCF-7/CerS4 and MCF- 7/ADR cells showed reduced ERα expression, CerS4 downregulation in MCF-7/ADR cells did not affect ERα expression, indicating that reduced ERα protein expression in MCF- 7/ADR cells is not caused by CerS4 overexpression. MCF-7/CerS4 and MCF-7/ADR cells share similar properties, such as drug resistance and high ABC transporter expression. However, the ABC transporter expression pattern in MCF-7/CerS4 cells differed from that in MCF-7/ADR cells. MCF-7/CerS4 cells displayed increased expression of ABCC1, ABCC2, ABCC4, ABCB1, and ABCG2, whereas MCF- 7/ADR cells showed increased expression of ABCC1, ABCC4, and ABCB1 but not ABCC2 and ABCG2. CerS4 knockdown in MCF-7/ADR cells only reduced ABCC1 and ABCB1 20 expression, suggesting that CerS4 may positively regulate ABCC1 and ABCB1, the most critical ABC transporters involved in the acquisition of chemoresistance. Consistently, CerS4 knockdown partially reversed the MDR phenotype in MCF-7/ADR cells upon treatment with doxorubicin, paclitaxel, or tamoxifen. These findings implicate CerS4 as an attractive target for overcoming chemoresistance in breast cancer, and treatment with various chemotherapeutic agents increased CerS4 expression (Supplementary Figure S2). The precise role of CerS4 in drug metabolism during chemotherapy remains to be further elucidated. Stable CerS4 overexpression in MCF-7 cells also promotes EMT and cell migration, resulting in the

upregulation of the **mesenchymal markers N-cadherin, vimentin, and** 59

Snail [50] and the downregulation of the epithelial marker E-cadherin [50]. The loss

of E-cadherin directly correlates **with** the loss **of the** epithelial **phenotype** 17

[50]. Interestingly, CerS4 knockdown restored these phenotypes in MCF-7/ADR cells, indicating that CerS4 governs the EMT process in breast cancer and that reducing CerS4 expression may contribute to the shift of mesenchymal-like cancer cells toward an epithelial state. During the EMT, the tightly connected

epithelial cells lose their polarity **and gain a migratory** mesenchymal **phenotype** 51

[51]. Therefore, long-term CerS4 overexpression in breast cancer may also play essential roles in cancer cell migration and metastasis. The present study introduced the possible oncogenic role of CerS4 in LumA breast cancer. CerS4 was found to be associated with many cancer-related signaling pathways, chemoresistance, and EMT. CerS4 overexpression also increased cell cycle and cell proliferation. Therefore, targeting CerS4 could serve as a novel therapeutic approach to inhibit tumor growth, enhance sensitivity to chemotherapy, and potentially prevent metastasis in LumA breast cancer

patients. Analyses of CerS4 expression profiles could help optimize treatment plans and avoid ineffective therapies in breast cancer patients. However, further research and clinical studies

will be necessary to fully validate the clinical relevance of 46

CerS4 and its therapeutic potential. Conclusions Long-term CerS4 overexpression promotes breast cancer progression and invasiveness by activating several cancer-related signaling pathways, such as Akt/mTOR, NF-κB, and β-catenin, and inducing EMT. In addition, CerS4 impacts chemoresistance via the positive regulation of MDR1 (ABCB1) and MRP1 (ABCC1), two key ABC transporters involved in MDR acquisition. Considering TCGA-BRCA data, which

revealed that higher CerS4 **expression was associated with** poor prognosis **in** 55

LumA, CerS4 might be applied as a potential prognostic or chemoresistance marker. Chronic alteration of CerS4 expression could critically impact breast cancer progression, metastasis and chemoresistance, positioning CerS4

as a novel target candidate **for breast cancer therapy** 7

. List of abbreviations ABC: ATP-binding cassette; AD: Adriamycin; Akt: protein kinase B; ANOVA: analysis of variance; BSA: bovine serum albutmin; CerS: ceramide synthase; DEG, differentially expressed gene; EGF: epidermal growth factor; EMT: epithelial?mesenchymal transition; ER: estrogen receptor; ERK: extracellular signal-regulated kinase; FASN: fatty acid synthase; FBS: fetal bovine serum; GEO: Gene Expression Omnibus;

GSK: glycogen synthase kinase; HER2: human epidermal growth factor receptor 2 3

; HMGCR:

HMG-CoA reductase; IC50: half maximal inhibitory concentration 12

; IGF:

insulin-like growth factor; LDLR: low- density lipoprotein receptor 50

; LumA: luminal subtype A; LumB: luminal subtype B; MCF- 7/ADR: Doxorubicin-resistant MCF-7 cell line; MCF-7/CerS4: CerS4-overexpressing MCF-7 stable cell line; MDR: multiple drug resistance; MRP: MDR-associated protein; mTOR: 22 mammalian target of rapamycin; NF-?B: nuclear factor-kappa B; PGR: progesterone receptor; RNA-seq: RNA-sequencing; SCD1: stearoyl-coenzyme A desaturase 1; SREBP: sterol regulatory element-binding protein; S6K: p90 ribosomal S6 kinase; TCGA-BRCA: the Cancer Genome Atlas Breast Invasive Carcinoma. Declarations Ethics approval and consent to participate: none Consent for publication: none Availability of data and materials: The datasets used and/or analysed during the current study are available from the corresponding author on reasonable request. Competing interests: The authors declare that they have no competing interests. Funding: This work was supported by the National Research Foundation of Korea grants funded by the Korean Government Ministry of Education, Science and Technology; grant no. NRF-2021R1F1A1045565 and 2021R1I1A3A04037479. Authors' contributions: JWP, SK, WJP contributed to the conception and design of the study. SJK, IS, MHK, and WJP performed the experiments and shared raw data for legitimacy. IS, JWP, SK and WJP contributed to the acquisition of data and wrote the manuscript. SJK, IS, MHK and WJP edited figures. IS, JWP, SK, and WJP reviewed and edited the manuscript. All authors read and approved the manuscript. Acknowledgements: This study met the publication guidelines provided by TCGA (http://www.cancer.gov/about-nci/organization/ccg/research/structrual-genomics/tcga/using- tcga/citing-tcga). Reference 1. Giaquinto AN, Sung H, Miller KD, Kramer JL, Newman LA, Minihan A, Jemal A, Siegel RL: Breast Cancer Statistics, 2022. CA Cancer J Clin 2022, 72:524-541. 2. Dai X, Li T, Bai Z, Yang Y, Liu X, Zhan J, Shi B: Breast cancer intrinsic subtype classification, clinical use and future trends. Am J Cancer Res 2015, 5:2929-2943. 3. Yersal O, Barutca S: Biological subtypes of breast cancer: Prognostic and therapeutic implications. World J Clin Oncol 2014, 5:412-424. 4. Zhou Y, Eppenberger-Castori S, Eppenberger U, Benz CC: The NFkappaB pathway and endocrine- resistant breast cancer. Endocr Relat Cancer 2005, 12 Suppl 1:S37-46. 5. Paplomata E, O'Regan R: The PI3K/AKT/mTOR pathway in breast cancer: targets, trials and biomarkers. Ther Adv Med Oncol 2014, 6:154-166. 6. Abreu de Oliveira WA, El Laithy Y, Bruna A, Annibali D, Lluis F: Wnt Signaling in the Breast: From Development to Disease. Front Cell Dev Biol 2022, 10:884467. 7. Wang W, Nag SA, Zhang R: Targeting the NFkappaB signaling pathways for breast cancer prevention and therapy. Curr Med Chem 2015, 22:264-289. 8. Watanabe R, Wei L, Huang J: mTOR Signaling, Function, Novel Inhibitors, and Therapeutic Targets. Journal of Nuclear Medicine 2011, 52:497. 9. Li H, Prever L, Hirsch E, Gulluni F: Targeting PI3K/AKT/mTOR Signaling Pathway in Breast Cancer. Cancers (Basel) 2021, 13. 10. Horton JD, Goldstein JL, Brown MS: SREBPs: activators of the complete program of cholesterol and fatty acid synthesis in the liver. J Clin Invest 2002, 109:1125-1131. 11. Bao J, Zhu L, Zhu Q, Su J, Liu M, Huang W: SREBP-1 is an independent prognostic marker and promotes invasion and migration in breast cancer. Oncol Lett 2016, 12:2409-2416. 12. Jie Z, Xie Z, Xu W, Zhao X, Jin G, Sun X, Huang B, Tang P, Wang G, Shen S, et al: SREBP-2 aggravates breast cancer associated osteolysis by promoting osteoclastogenesis and breast cancer metastasis. Biochim Biophys Acta Mol Basis Dis 2019, 1865:115-125. 13. Sun X, Wang M, Wang M, Yao L, Li X, Dong H, Li M, Li X, Liu X, Xu Y: Exploring the Metabolic Vulnerabilities of Epithelial-Mesenchymal Transition in Breast Cancer. Front Cell Dev Biol 2020, 8:655. 14. He J, Fortunati E, Liu DX, Li Y: Pleiotropic Roles of ABC Transporters in Breast Cancer. Int J Mol Sci 2021, 22. 15. Ogretmen B: Sphingolipid metabolism in cancer signalling and therapy. Nat Rev Cancer 2018, 18:33- 50. 16. Park JW, Park WJ, Futerman AH: Ceramide synthases as potential targets for therapeutic intervention in human diseases. Biochim Biophys Acta 2014, 1841:671-681. 17. Park WJ, Park JW: The effect of altered sphingolipid acyl chain length on various disease models. Biol Chem 2015, 396:693-705. 18. Kim YR, Lee EJ, Shin KO, Kim MH, Pewzner-Jung Y, Lee YM, Park JW, Futerman AH, Park WJ: Hepatic triglyceride accumulation via endoplasmic reticulum stress-induced SREBP-1 activation is regulated by ceramide synthases. Exp Mol Med 2019, 51:129. 19.

Mesicek J, Lee H, Feldman T, Jiang X, Skobeleva A, Berdyshev EV, Haimovitz-Friedman A, Fuks Z, Kolesnick R: Ceramide synthases 2, 5, and 6 confer distinct roles in radiation-induced apoptosis in HeLa cells. Cell Signal 2010, 22:1300-1307. 20. Levy M, Futerman AH: Mammalian ceramide synthases. IUBMB Life 2010, 62:347-356. 21. Schiffmann S, Sandner J, Birod K, Wobst I, Angioni C, Ruckhäberle E, Kaufmann M, Ackermann H, Lötsch J, Schmidt H, et al: Ceramide synthases and ceramide levels are increased in breast cancer tissue. Carcinogenesis 2009, 30:745-752. 22. Erez-Roman R, Pienik R, Futerman AH: Increased ceramide synthase 2 and 6 mRNA levels in breast cancer tissues and correlation with sphingosine kinase expression. Biochem Biophys Res Commun 2010, 391:219-223. 23. Pani T, Rajput K, Kar A, Sharma H, Basak R, Medatwal N, Saha S, Dev G, Kumar S, Gupta S, et al: Alternative splicing of ceramide synthase 2 alters levels of specific ceramides and modulates cancer cell proliferation and migration in Luminal B breast cancer subtype. Cell Death Dis 2021, 12:171. 24. Hartmann D, Lucks J, Fuchs S, Schiffmann S, Schreiber Y, Ferreiros N, Merkens J, Marschalek R, Geisslinger G, Grosch S: Long chain ceramides and very long chain ceramides have opposite effects on human breast and colon cancer cell growth. Int J Biochem Cell Biol 2012, 44:620-628. 25. Wegner MS, Wanger RA, Oertel S, Brachtendorf S, Hartmann D, Schiffmann S, Marschalek R, Schreiber Y, Ferreiros N, Geisslinger G, Grosch S: Ceramide synthases CerS4 and CerS5 are upregulated by 17beta-estradiol and GPER1 via AP-1 in human breast cancer cells. Biochem Pharmacol 2014, 92:577-589. 26. Kovalchuk O, Filkowski J, Meservy J, Ilnytskyy Y, Tryndyak VP, Chekhun VF, Pogribny IP: Involvement of microRNA-451 in resistance of the MCF-7 breast cancer cells to chemotherapeutic drug doxorubicin. Mol Cancer Ther 2008, 7:2152-2159. 27. Kim YR, Lee EJ, Shin KO, Kim MH, Pewzner-Jung Y, Lee YM, Park JW, Futerman AH, Park WJ: Hepatic triglyceride accumulation via endoplasmic reticulum stress-induced SREBP-1 activation is regulated by ceramide synthases. Exp Mol Med 2019, 51:1-16. 28. Dobin A, Davis CA, Schlesinger F, Drenkow J, Zaleski C, Jha S, Batut P, Chaisson M, Gingeras TR: STAR: ultrafast universal RNA-seq aligner. Bioinformatics 2013, 29:15-21. 29. Love MI, Huber W, Anders S: Moderated estimation of fold change and dispersion for RNA-seq data with DESeq2. Genome Biol 2014, 15:550. 30. Davis S, Meltzer PS: GEOquery: a bridge between the Gene Expression Omnibus (GEO) and BioConductor. Bioinformatics 2007, 23:1846-1847. 31. Parker JS, Mullins M, Cheang MC, Leung S, Voduc D, Vickery T, Davies S, Fauron C, He X, Hu Z, et al: Supervised risk predictor of breast cancer based on intrinsic subtypes. J Clin Oncol 2009, 27:1160-1167. 32. Ritchie ME, Phipson B, Wu D, Hu Y, Law CW, Shi W, Smyth GK: limma powers differential expression analyses for RNA-sequencing and microarray studies. Nucleic Acids Res 2015, 43:e47. 33. Ritz C, Baty F, Streibig JC, Gerhard D: Dose-Response Analysis Using R. PLoS One 2015, 10:e0146021. 34. Xie Z, Bailey A, Kuleshov MV, Clarke DJB, Evangelista JE, Jenkins SL, Lachmann A, Wojciechowicz ML, Kropiwnicki E, Jagodnik KM, et al: Gene Set Knowledge Discovery with Enrichr. Curr Protoc 2021, 1:e90. 35. Salt MB, Bandyopadhyay S, McCormick F: Epithelial-to-mesenchymal transition rewires the molecular path to PI3K-dependent proliferation. Cancer Discov 2014, 4:186-199. 36. Moon HR, Ospina-Munoz N, Noe-Kim V, Yang Y, Elzey BD, Konieczny SF, Han B: Subtype-specific characterization of breast cancer invasion using a microfluidic tumor platform. PLoS One 2020, 15:e0234012. 37. Comsa S, Cimpean AM, Raica M: The Story of MCF-7 Breast Cancer Cell Line: 40 years of Experience in Research. Anticancer Res 2015, 35:3147-3154. 38. Igal RA: Stearoyl CoA desaturase-1: New insights into a central regulator of cancer metabolism. Biochim Biophys Acta 2016, 1861:1865-1880. 39. Menendez JA, Lupu R: Fatty acid synthase (FASN) as a therapeutic target in breast cancer. Expert Opin Ther Targets 2017, 21:1001-1016. 40. Glavinas H, Krajcsi P, Cserepes J, Sarkadi B: The role of ABC transporters in drug resistance, metabolism and toxicity. Curr Drug Deliv 2004, 1:27-42. 41. Cuvillier O, Pirianov G, Kleuser B, Vanek PG, Coso OA, Gutkind S, Spiegel S: Suppression of ceramide-mediated programmed cell death by sphingosine-1-phosphate. Nature 1996, 381:800-803. 42. Dany M, Ogretmen B: Ceramide induced mitophagy and tumor suppression. Biochim Biophys Acta 2015, 1853:2834-2845. 43. Saddoughi SA,

Ogretmen B: Diverse functions of ceramide in cancer cell death and proliferation. Adv Cancer Res 2013, 117:37-58. 44. Hartmann D, Lucks J, Fuchs S, Schiffmann S, Schreiber Y, Ferreirós N, Merkens J, Marschalek R, Geisslinger G, Grösch S: Long chain ceramides and very long chain ceramides have opposite effects on human breast and colon cancer cell growth. Int J Biochem Cell Biol 2012, 44:620-628. 45. Catalano A, Iacopetta D, Ceramella J, Scumaci D, Giuzio F, Saturnino C, Aquaro S, Rosano C, Sinicropi 652 MS: Multidrug Resistance (MDR): A Widespread Phenomenon in Pharmacological Therapies. Molecules 2022, 27. 46. Sun YL, Patel A, Kumar P, Chen ZS: Role of ABC transporters in cancer chemotherapy. Chin J Cancer 2012, 31:51-57. 47. Chen M, Cui YK, Huang WH, Man K, Zhang GJ: Phosphorylation of estrogen receptor α at serine 118 is correlated with breast cancer resistance to tamoxifen. Oncol Lett 2013, 6:118-124. 48. de Leeuw R, Neefjes J, Michalides R: A role for estrogen receptor phosphorylation in the resistance to tamoxifen. Int J Breast Cancer 2011, 2011:232435. 49. Morandi A, Plaza-Menacho I, Isacke CM: RET in breast cancer: functional and therapeutic implications. Trends Mol Med 2011, 17:149-157. 50. Thiery JP: Epithelial-mesenchymal transitions in tumour progression. Nat Rev Cancer 2002, 2:442- 454. 51. Leggett SE, Hruska AM, Guo M, Wong IY: The epithelial-mesenchymal transition and the cytoskeleton in bioengineered systems. Cell Commun Signal 2021, 19:32.
Figure legends Figure 1. CERS4 expression levels and prognostic significance in patients with breast cancer. (A) CERS4 expression in breast cancer tissue (tumor) and adjacent normal breast tissue. (B) CERS4 expression in LumA and PAM50 molecular breast cancer subtypes. (C) Kaplan?Meier plot showing survival among individuals with LumA breast cancer in the higher and lower CERS4 expression groups. The number of subjects is shown in parentheses. Two-tailed Student's t-test was performed for (A and B). The log-rank test was performed for (C). Significance is indicated as follows: **P < 0.01 and #P < 0.0001. Figure 2. Establishment of a CerS4 overexpressing MCF-7 cell line. MCF-7 cells overexpressing CerS4 were selected by G418 treatment. (A) Relative mRNA expression levels of various CerS family members in MCF-7 and CerS4-overexpressing MCF-7 cells (MCF-7/CerS4) (n=3). (B) Ceramide levels in MCF-7 and CerS4-overexpressing MCF-7 cells (MCF-7/CerS4) using liquid chromatography-electrospray ionization-tandem mass spectrometry (LC-ESI-MS/MS) (n=3). (C) Representative western blots showing protein expression levels of various CerS family members (left) and densitometric analysis (right) of CerS protein levels in MCF-7 and MCF-7/CerS4 cells (n=3). (D) Cell proliferation in MCF-7 and MCF-7/CerS4 cells (n=3). A two-tailed Student's t-test was performed for (A, B, and C). A two-way ANOVA and a Tukey post hoc test was performed for (D). Significance is indicated as follows: *P < 0.05, **P < 0.01, and ***P < 0.001. Figure 3. Multiple cancer-related pathways and receptors are activated in CerS4-overexpressing MCF-7 cells. (A) Well-known tumor-related pathway components (Akt/mTOR, NF-κB, and GSK3β/β-catenin) (left) and densitometric analysis (right) of the indicated protein in MCF-7 and MCF-7/CerS4 cells (n=3) (B) β-catenin levels in nuclear and cytosol fractions were assessed in MCF-7 and CerS4-overexpressing MCF-7 cells (MCF-7/CerS4). (C) Relative ESR1, ESR2, and PGR mRNA expression levels in MCF-7 and MCF- 7/CerS4 cells. (D) Representative western blots showing protein levels of ERα, PGR, and phosphorylated (Ser118, Ser167) ERα and (E) their densitometric analysis in MCF-7 and MCF-7/CerS4 cells (n=3). (F)

Increased expression of lipid- **related genes in MCF** -7/CerS4 **cells** 10

compared with MCF-7 cells (n=3). Two-tailed Student's t-test was performed. Significance is indicated as follows: *P < 0.05, **P < 0.01, and ***P < 0.001. Akt, protein kinase B; ERα, estrogen receptor alpha; ERK, extracellular signal-related

kinase; FAS, fatty acid synthase; GAPDH, glyceraldehyde 3-phosphate dehydrogenase; GSK3β, glycogen synthase kinase-3 betta; HA, hemi-agglutinin; HMGCR, 3-hydroxy-3-methylglutaryl-CoA reductase; LDLR,

low-density lipoprotein receptor ; mTOR, **mammalian target of rapamycin** 21

; NF-κB, nuclear factor kappa B; PGR, progesterone receptor; S6K,

ribosomal S6 kinase; SCD1, stearoyl-CoA desaturase 1 61

; SREBP, sterol regulatory element–binding protein. Figure 4. Increased resistance to chemotherapeutic agents in CerS4-overexpressing MCF-7 cells. (A–C) Cell death following treatment of MCF-7 and CerS4-overexpressing MCF-7 cells (MCF-7/CerS4) with (A) doxorubicin, (B) paclitaxel, or (C) tamoxifen (n=3). IC50, half-maximal inhibitory concentration. (D) Expression of ATP-binding cassette (ABC) genes in MCF-7/CerS4 cells was assessed by RNA sequencing (n=3). (E) Relative mRNA levels of ABC transporters (ABCC1, ABCC2, ABCC4, ABCC11, ABCB1, ABCB4, and ABCG2) was assessed by real-time PCR (n=3). A two-way ANOVA and a Tukey post hoc test was performed for (A, B, and C). The adjusted P-values obtained from DESeq2-analysis for (D). A two-tailed Student's t-test was performed for (E). Significance is indicated as follows: *P < 0.05, **P < 0.01, and ***P < 0.001. 28 Figure 5. Expression of EMT markers is increased in CerS4-overexpressing MCF-7 cells. (

A) Representative western blots of an **epithelial marker (E-cadherin**) and **mesenchymal** markers (N-cadherin, **vimentin, and** 5

Snail) (left) and their densitometric analysis (right) in MCF-7 and MCF-7/CerS4 cells (n=3). (B) EMT scores in MCF-7 and MCF-7/CerS4 cells (n=3). (C) Cell migration in MCF-7 and MCF-7/CerS4 cells (n=3). Two-tailed Student's t- test was performed. Significance is indicated as follows :*P < 0.05, **P < 0.01. Figure 6. Downregulation of CerS4 in MCF-7/ADR cells partially reverses multiple drug resistance. (A) mRNA expression of various CerS family members in MCF-7 cells and MCF-7/ADR before and after (MCF-7/ADR+shCerS4) CerS4 downregulation (n=3). (B) Ceramide levels in MCF-7 and MCF-7/ADR before and after (MCF-7/ADR+shCerS4) CerS4 downregulation using LC-ESI-MS/MS (n=3). (C–E) Cell death was examined in MCF-7, MCF-7/ADR, and MCF-7/ADR+shCerS4 cells following treatment with (C) doxorubicin, (D) paclitaxel, or (E) tamoxifen (n=3). IC50, half-maximal inhibitory concentration. A one- way ANOVA was performed for (A and B), and a two-way ANOVA was performed for (C, D, and E), respectively. Additionally, a Tukey post hoc test was conducted for each analysis. Significance is indicated compared to control group; *P < 0.05, **P < 0.01, ***P < 0.001 and compared to MCF-7/ADR group; ##P < 0.01, ###P<0.001. Figure 7. CerS4 downregulation in MCF-7/ADR cells partially reverses cancer-related pathway activation and increases ABC gene expression. (A) Representative western blot analysis of the indicated protein levels (left) and their densitometric analysis (right) in MCF-7 cells and MCF-7/ADR before and after (MCF-7/ADR+shCerS4) CerS4 downregulation (n=3). (B) Relative mRNA levels of ATP-binding cassette (ABC) transporters in MCF- 29 7/ADR cells, using data from GSE24460 (n=2). (C) Relative mRNA levels of ABC

transporters (ABCC1, ABCC2, ABCC4, ABCB1, and ABCG2) in MCF-7/ADR cells before and after (MCF-7/ADR+shCerS4) CerS4 downregulation (

n=3). A **one-way ANOVA** and a Tukey **post hoc test was performed** 39

for (A and C). The adjusted P-values obtained from limma-analysis for (B). Significance is indicated as follows: *P < 0.05, **P < 0.01, and ***P < 0.001. Akt,

protein kinase B; GSK3β, glycogen synthase kinase-3 beta; mTOR, mammalian target of rapamycin 27

; S6K, ribosomal S6 kinase. Figure 8. Downregulation of CerS4 in MCF-7/ADR cells reverses EMT. (A) The number of shared DEG between CerS4-overexpressing MCF-7 cells (MCF-7/CerS4) and MCF- 7/ADR. (B) EMT score in MCF-7/ADR cells, calculated using data from GSE24460 (n=2). (C, D)

Representative western blots of an **epithelial marker (E-cadherin**) and **mesenchymal** markers (N-cadherin, **vimentin, and** 5

Snail) (C) and their densitometric analysis (D) in MCF-7 cells and MCF-7/ADR cells before and after (MCF-7/ADR+shCerS4) CerS4 downregulation (n=3). (E) Cell migration in MCF-7, MCF-7/ADR, and MCF-7/ADR+shCerS4 cells (n=3). A two-tailed Student’s t-test was performed for (B). A one-way ANOVA and a Tukey post hoc test was performed for (D). Significance is indicated as follows: *P < 0.05, **P < 0.01, and ***P < 0.001. 1 2 3 4 5 6 7 8 9 10 11 12 13 14 15 16 17 18 19 20 21 22 23 24 25 26 27 28 29 30 31 32 33 34 35 36 37 38 39 40 41 42 43 44 45 46 47 48 49 50 51 52 53 54 55 56 57 58 59 60 61 62 63 64 65 66 67 68 69 70 71 72 73 74 75 76 77 78 79 80 81 82 83 84 85 86 87 88 89 90 91 92 93 94 95 96 97 98 99 100 101 102 103 104 105 106 107 108 109 110 111 112 113 114 115 116 117 118 119 120 121 122 123 124 125 126 127 128 129 130 131 132 133 134 135 136 137 138 139 140 141 142 143 144 145 146 147 148 149 150 151 152 153 154 155 156 157 158 159 160 161 162 163 164 165 166 167 168 169 170 171 172 173 174 175 176 177 178 179 180 181 182 183 184 185 186 187 188 189 190 191 192 193 194 195 196 197 198 199 200 201 202 203 204 205 206 207 208 209 210 211 212 213 214 215 216 217 218 219 220 221 222 223 224 225 226 227 228 229 230 231 232 233 234 235 236 237 238 239 240 241 242 243 244 245 246 247 248 249 250 251 252 253 254 255 256 257 258 259 260 261 262 263 264 265 266 267 268 269 270 271 272 273 274 275 276 277 295 296 297 298 299 300 301 302 303 304 305 306 307 308 309 310 311 312 313 314 315 316 317 318 337 338 339 340 341 342 343 344 345 346 347 348 349 350 351 352 353 354 355 356 357 358 359 360 361 362 363 364 365 366 367 368 369 370 371 372 373 374 375 376 377 378 379 380 381 382 383 384 385 386 387 388 389 390 391 392 393 394 395 396 397 398 399 400 406 407 408 409 410 411 412 413 414 415 416 417 418 419 420 421 422 423 424 425 426 427 428 429 430 431 432 433 434 435 436 437 438 439 440 441 442 443 444 445 446 447 448 449 450 451 452 453 454 455 456 457 458 459 460 461 462 463 464 465 466 467 468 469 470 471 472 473 474 475 476 477 478 479 480 481 482 483 484

485 486 487 488 489 490 491 492 493 494 495 496 497 498 499 500 501 502 503 504 505 506 507 508 509 510 511 512 513 514 515 516 517 518 519 520 521 522 523 524 525 526 527 528 529 530 531 532 533 534 535 536 537 538 539 540 541 542 543 544 545 546 547 548 549 550 551 552 553 554 555 556 557 558 559 560 561 562 563 564 565 566 567 568 569 570 571 572 573 574 575 576 577 578 579 580 581 582 583 584 585 586 587 588 589 590 591 592 593 594 595 596 597 598 599 600 601 602 603 604 605 606 607 608 609 610 611 612 613 614 615 616 617 618 619 620 621 622 623 624 625 626 627 628 629 630 631 632 633 634 635 636 637 638 639 640 641 642 643 644 645 646 647 648 649 650 651 Fig. 01 Fig.02 Fig.03 Fig.04 Fig.05 Fig.06 Fig.07 Fig.08 1 6 8 9 11 12 13 336 403 18 21 23 24 25 26 27 30 31 32 33 34 35 36 37 38

sources:

1 16 words / < 1% match - from 21-Mar-2023 12:00AM
docksci.com

2 14 words / < 1% match - Internet from 26-Jul-2022 12:00AM
docksci.com

3 11 words / < 1% match - from 16-May-2023 12:00AM
docksci.com

4 10 words / < 1% match - from 23-May-2023 12:00AM
docksci.com

5 21 words / < 1% match - Internet from 08-Jul-2020 12:00AM
www.wjgnet.com

6 8 words / < 1% match - Internet from 03-Oct-2022 12:00AM
www.wjgnet.com

7 8 words / < 1% match - Internet from 16-Jan-2023 12:00AM
www.wjgnet.com

8 13 words / < 1% match - from 29-Jun-2023 12:00AM
journals.plos.org

9 10 words / < 1% match - Internet from 13-Jan-2023 12:00AM
journals.plos.org

10 8 words / < 1% match - Internet from 12-Mar-2019 12:00AM
journals.plos.org

11 21 words / < 1% match - Internet from 30-Mar-2016 12:00AM
www.dovepress.com

12 8 words / < 1% match - Internet from 11-Nov-2021 12:00AM
www.dovepress.com

13 25 words / < 1% match - Crossref Posted Content
Stephanie F. Maurina, John P. O'Sullivan, Geetika Sharma, Daniel C. Pineda Rodriguez et al. "An evolutionarily conserved strategy for ribosome binding and inhibition by β-coronavirus non-structural protein 1", Cold Spring Harbor Laboratory, 2023

14 21 words / < 1% match - Internet from 18-Dec-2022 12:00AM
digitalcommons.wustl.edu

15 20 words / < 1% match - from 19-Mar-2023 12:00AM
link.springer.com

16 12 words / < 1% match - Internet from 22-Dec-2021 12:00AM
www.spandidos-publications.com

17 8 words / < 1% match - Internet from 08-Feb-2021 12:00AM
www.spandidos-publications.com

18 19 words / < 1% match - Internet from 29-Oct-2020 12:00AM
ar.iiarjournals.org

19 19 words / < 1% match - Internet from 19-Jan-2023 12:00AM
core.ac.uk

20 10 words / < 1% match - Internet from 10-Jan-2022 12:00AM
coek.info

21 8 words / < 1% match - Internet from 19-Feb-2022 12:00AM
coek.info

22 18 words / < 1% match - Internet from 11-Jan-2021 12:00AM
test.dovepress.com

23 16 words / < 1% match - Internet from 29-Sep-2009 12:00AM
hepatitis-central.com

24 8 words / < 1% match - from 15-Jun-2023 12:00AM
www.mdpi.com

25 8 words / < 1% match - Internet from 01-Oct-2022 12:00AM
www.mdpi.com

26 15 words / < 1% match - Internet from 02-Dec-2021 12:00AM
booksc.org

27 14 words / < 1% match - Crossref
Mohamad Hafizi Abu Bakar, Cheng Kian Kai, Wan Najihah Wan Hassan, Mohamad Roji Sarmidi, Harisun Yaakob, Hasniza Zaman Huri. "Mitochondrial dysfunction as a central event for mechanisms underlying insulin resistance: the roles of long chain fatty acids", Diabetes/Metabolism Research and Reviews, 2015

28 14 words / < 1% match - Internet from 12-Sep-2022 12:00AM
animaldairy.uga.edu

29 14 words / < 1% match - Internet from 29-Oct-2020 12:00AM
kuscholarworks.ku.edu

30 14 words / < 1% match - Internet from 06-Dec-2020 12:00AM
www.thieme-connect.com

31 13 words / < 1% match - Internet from 22-Feb-2023 12:00AM
encyclopedia.pub

32 13 words / < 1% match - Internet from 28-Sep-2021 12:00AM
www.hindawi.com

33 12 words / < 1% match - Crossref
Schoebel, Corine N., Stefan Zoller, and Daniel Rigling. "Detection and genetic characterisation of a novel mycovirus in Hymenoscyphus fraxineus, the causal agent of ash dieback", Infection Genetics and Evolution, 2014.

34 11 words / < 1% match - Crossref
Stefano Mangiola, Ryan Stuchbery, Geoff Macintyre, Michael J Clarkson et al. "Periprostatic fat tissue transcriptome reveals a signature diagnostic for high-risk prostate cancer", Endocrine-Related Cancer, 2018

35 11 words / < 1% match - Internet from 10-Oct-2022 12:00AM
e-century.us

36 11 words / < 1% match - Internet from 07-May-2020 12:00AM
obgyn.pericles-prod.literatumonline.com

37 11 words / < 1% match - Internet from 12-Dec-2022 12:00AM
static.frontiersin.org

38 11 words / < 1% match - Internet from 04-Mar-2022 12:00AM
www.dignityhealth.org

39 10 words / < 1% match - Crossref Posted Content

Inga Mohr, Amin Mirzaiebadizi, Sibaji K. Sanyal, Pichaporn Chuenban, Mohammad R. Ahmadian, Rumen Ivanov, Petra Bauer. " Characterization of the small GTPase and ADP-ribosylation factor-like 2 protein TITAN5 ", Cold Spring Harbor Laboratory, 2023

40 10 words / < 1% match - Crossref
John C. Widen, Aaron M. Kempema, Peter W. Villalta, Daniel A. Harki. "Targeting NF-κB p65 with a Helenalin Inspired Bis-electrophile", ACS Chemical Biology, 2016

41 10 words / < 1% match - Crossref
Zahra Soleimani, Davood Kheirkhah, Mohammad Reza Sharif, Alireza Sharif, Mohammad Karimian, Younes Aftabi. "Association of CCND1 Gene c.870G>A Polymorphism with Breast Cancer Risk: A Case-ControlStudy and a Meta-Analysis", Pathology & Oncology Research, 2016

42 10 words / < 1% match - Internet from 28-Nov-2022 12:00AM
academic.oup.com

43 10 words / < 1% match - Internet from 02-Jul-2022 12:00AM
github.com

44 9 words / < 1% match - Crossref
J.-R. Weng. "OSU-A9, a Potent Indole-3-Carbinol Derivative, Suppresses Breast Tumor Growth by Targeting the Akt-NF-B Pathway and Stress Response Signaling", Carcinogenesis, 08/25/2009

45 9 words / < 1% match - Crossref
Prabhat C. Goswami. "Phospholipid Hydroperoxide Glutathione Peroxidase Induces a Delay in G 1 of the Cell Cycle", Free Radical Research, 6/1/2003

46 9 words / < 1% match - Crossref
Sachi Horibata, Edward J. Rice, Hui Zheng, Chinatsu Mukai, Tinyi Chu, Brooke A. Marks, Scott A. Coonrod, Charles G. Danko. "A bi-stable feedback loop between GDNF, EGR1, and ERα contribute to endocrine resistant breast cancer", PLOS ONE, 2018

47 9 words / < 1% match - ProQuest
Zayas, Candilianne Serrano. "Molecular Regulation of Low-Density Lipoprotein Receptor Expression", University of Michigan, 2023

48 9 words / < 1% match - Internet from 16-Aug-2017 12:00AM
ajcn.nutrition.org

49 9 words / < 1% match - from 28-Mar-2023 12:00AM
brieflands.com

50 9 words / < 1% match - Internet from 11-Jan-2023 12:00AM
journals.lww.com

51 9 words / < 1% match - Internet from 01-Dec-2017 12:00AM
mdpi.com

52 9 words / < 1% match - Internet from 02-May-2016 12:00AM
spandidos-publications.com

53 9 words / < 1% match - Internet from 16-Oct-2022 12:00AM
www.jcancer.org

54 8 words / < 1% match - Crossref
Luciano Vellon. "Up-regulation of α_Vβ₃ integrin expression is a novel molecular response to chemotherapy-induced cell damage in a heregulin-dependent manner", Differentiation, 11/2007

55 8 words / < 1% match - Crossref
Rasha Irshad, Sazi Tabassum, Mohammad Husain. "Aberrant Lipid Metabolism in Cancer: Current Status and Emerging Therapeutic Perspectives", Current Topics in Medicinal Chemistry, 2023

56 8 words / < 1% match - Crossref
X. Lin. "Overexpression of PKC is required to impart estradiol inhibition and tamoxifen-resistance in a T47D human breast cancer tumor model", Carcinogenesis, 12/12/2005

57 8 words / < 1% match - Internet from 25-Mar-2016 12:00AM
eprints.whiterose.ac.uk

58 8 words / < 1% match - Internet from 29-Nov-2022 12:00AM
etd.lib.metu.edu.tr

59 8 words / < 1% match - Internet from 15-Nov-2022 12:00AM
mdpi-res.com

60 8 words / < 1% match - Internet from 18-Jan-2023 12:00AM
worldwidescience.org

61 8 words / < 1% match - Internet from 05-Aug-2017 12:00AM
www.jlr.org

62 8 words / < 1% match - Internet from 28-Jul-2020 12:00AM
www.phcog.com

63 8 words / < 1% match - Internet from 16-Mar-2019 12:00AM
www.tandfonline.com

64 7 words / < 1% match - Crossref
S. Kalla Singh, D. Moretta, F. Almaguel, M. De León, Daisy D. De León. " Precursor Igf-ii (Proigf-ii) And Mature Igf-ii (Migf-ii) Induce Bcl-2 And Bcl-x Expression Through Different Signaling Pathways In Breast Cancer Cells ", Growth Factors, 2009

65 7 words / < 1% match - Crossref
Xian-Guo Meng, Shou-Wei Yue. "Dexamethasone Disrupts Cytoskeleton Organization and Migration of T47D Human Breast Cancer Cells by Modulating the AKT/mTOR/RhoA Pathway",

Asian Pacific Journal of Cancer Prevention, 2015

66	6 words / < 1% match - Crossref<br>Jiaoyu Ai, Sonja M. Wörmann, Kıvanç Görgülü, Mireia Vallespinos et al. "Bcl3 Couples Cancer Stem Cell Enrichment With Pancreatic Cancer Molecular Subtypes", Gastroenterology, 2021
67	6 words / < 1% match - Crossref<br>P.A. Borea, S. Gessi, S. Bar-Yehuda, P. Fishman. "Chapter 10 A3 Adenosine Receptor: Pharmacology and Role in Disease", Springer Science and Business Media LLC, 2009
68	6 words / < 1% match - Crossref<br>Russo, Sarah Brice, Catalin F. Baicu, An Van Laer, Tuoyu Geng, Harinath Kasiganesan, Michael R. Zile, and L. Ashley Cowart. "Ceramide synthase 5 mediates lipid-induced autophagy and hypertrophy in cardiomyocytes", Journal of Clinical Investigation, 2012.
69	6 words / < 1% match - Internet from 13-Feb-2023 12:00AM<br>patents.google.com
70	6 words / < 1% match - Internet from 11-Jul-2020 12:00AM<br>www.jove.com
