# Supplementary material for: Ceramide synthase 4 overexpression exerts oncogenic properties in breast cancer
Source: Lipids Health Dis. 2023 Oct 26;22:183. doi: 10.1186/s12944-023-01930-z (PMC10605224; doi:10.1186/s12944-023-01930-z)

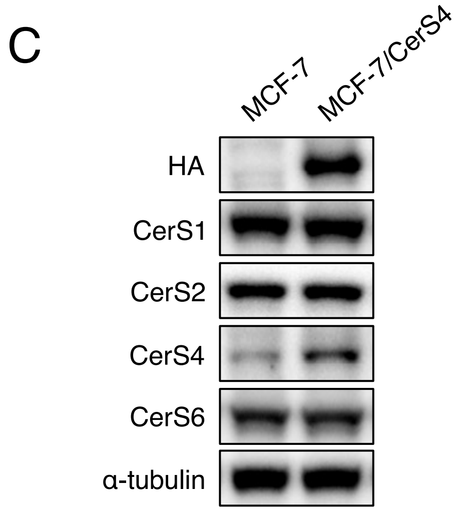
C
MCF-7
MCF-7/CerS4
HA
CerS1
CerS2
CerS4
CerS6
α-tubulin

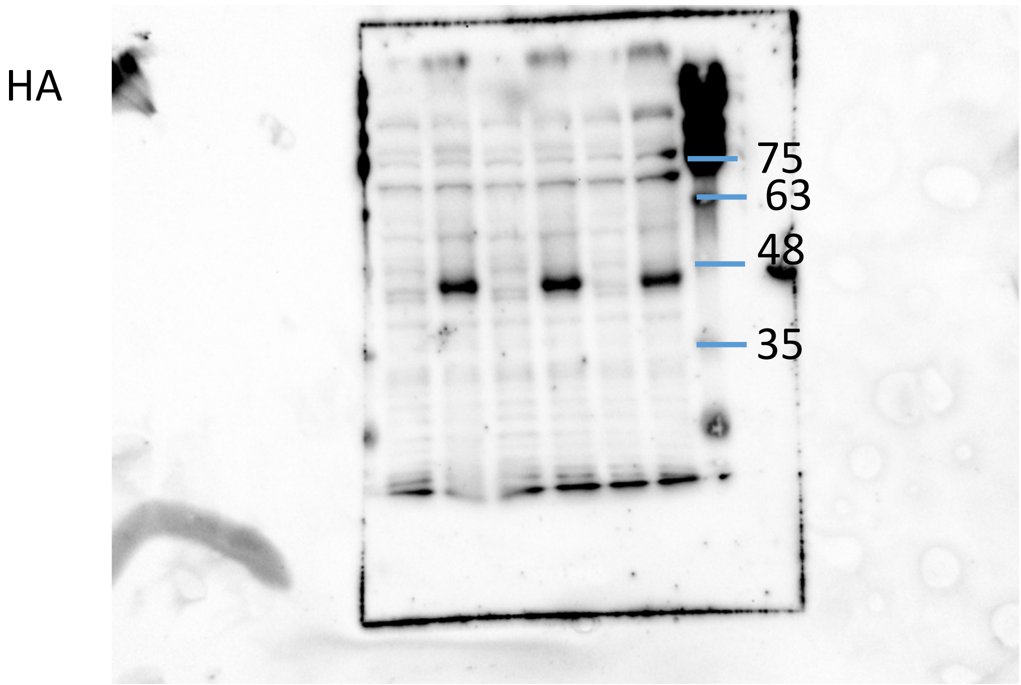
HA
75
63
48
35

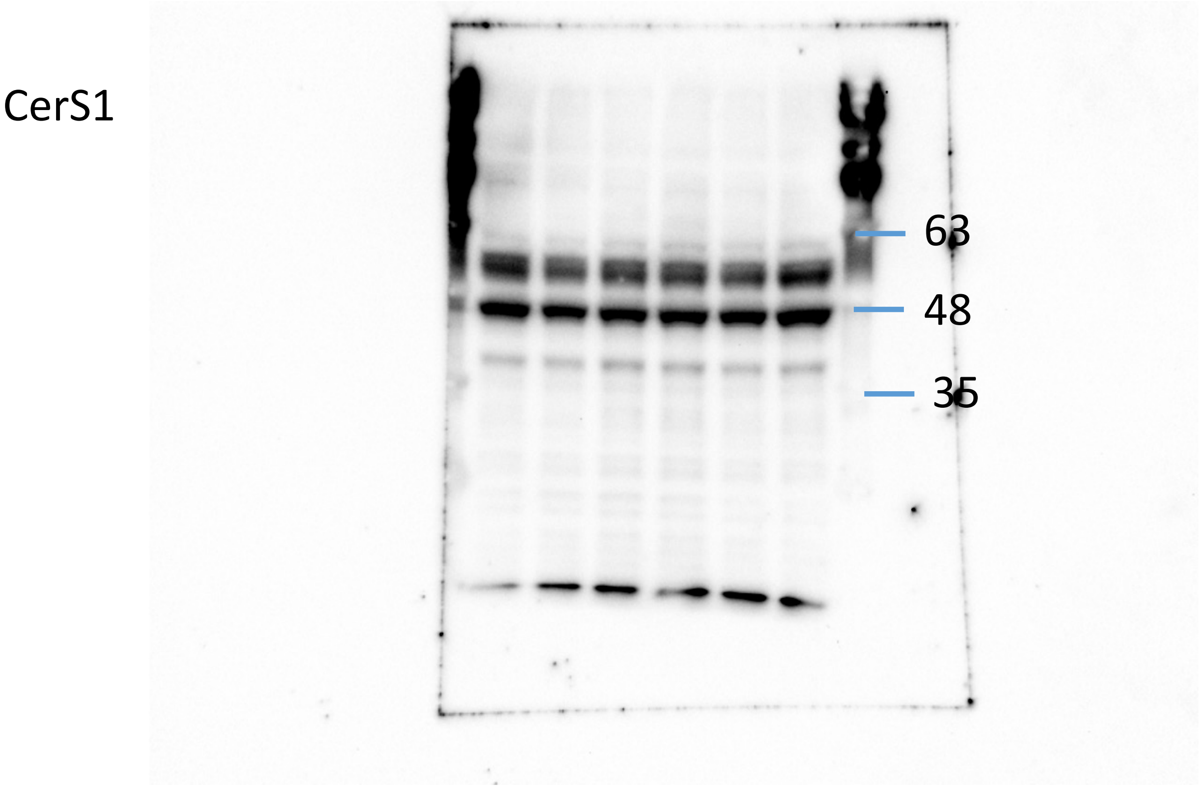
CerS1
63
48
35

CerS2

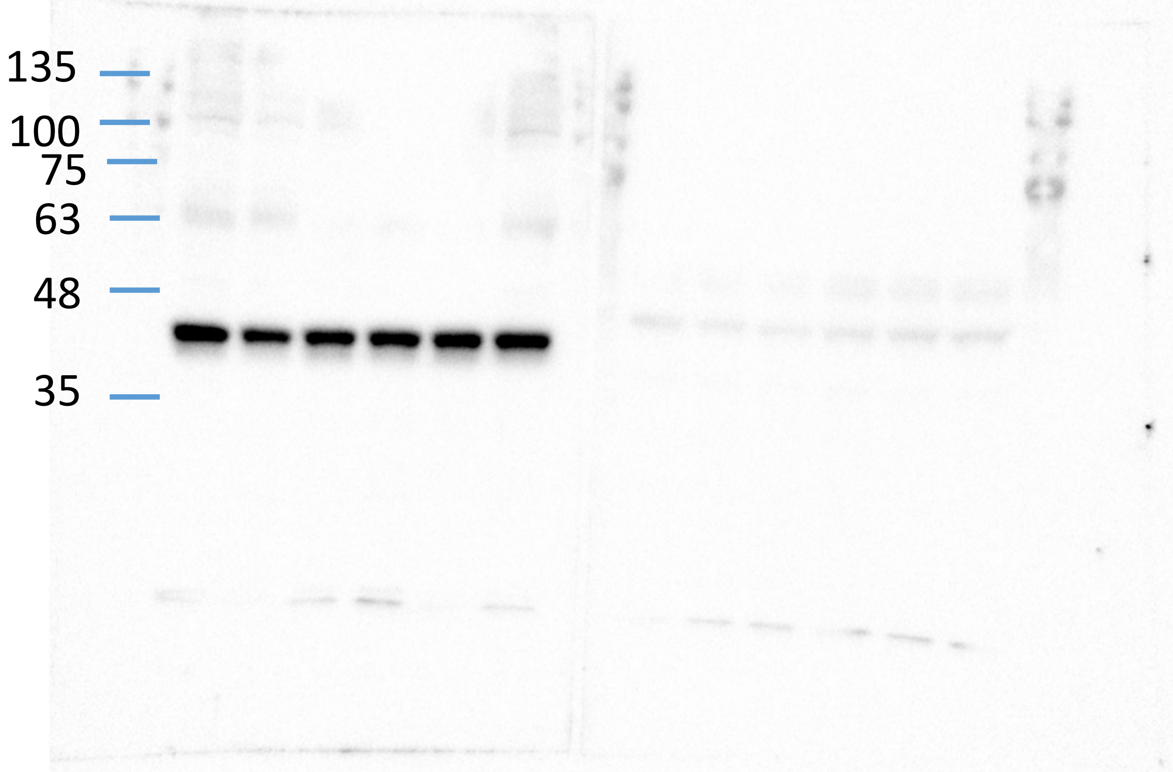

CerS4

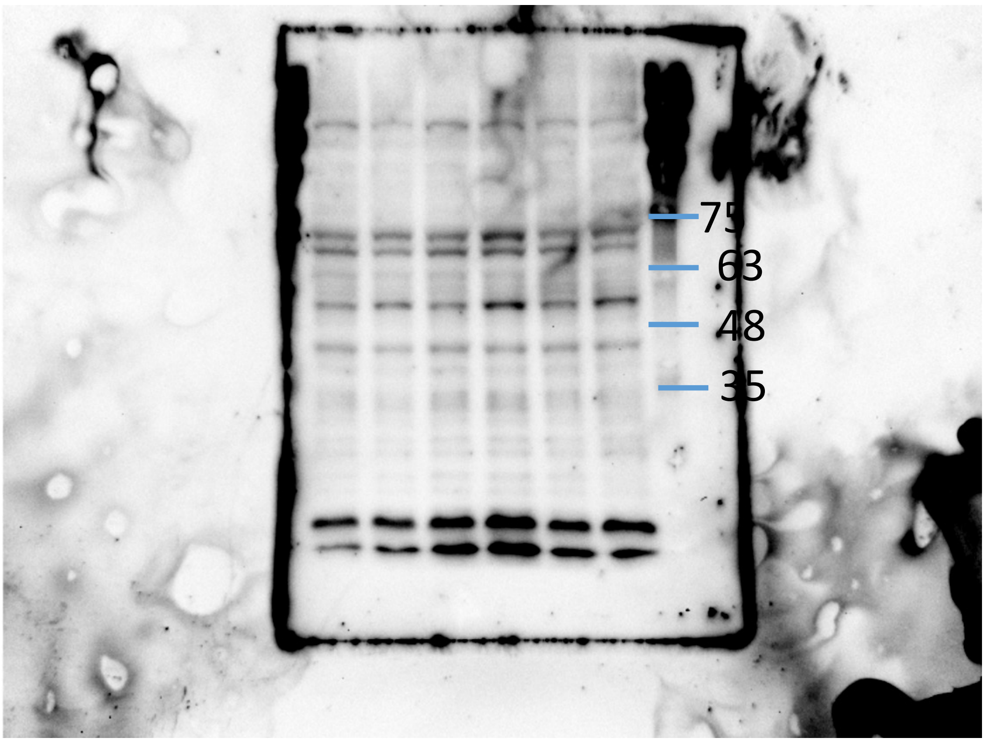

CerS6

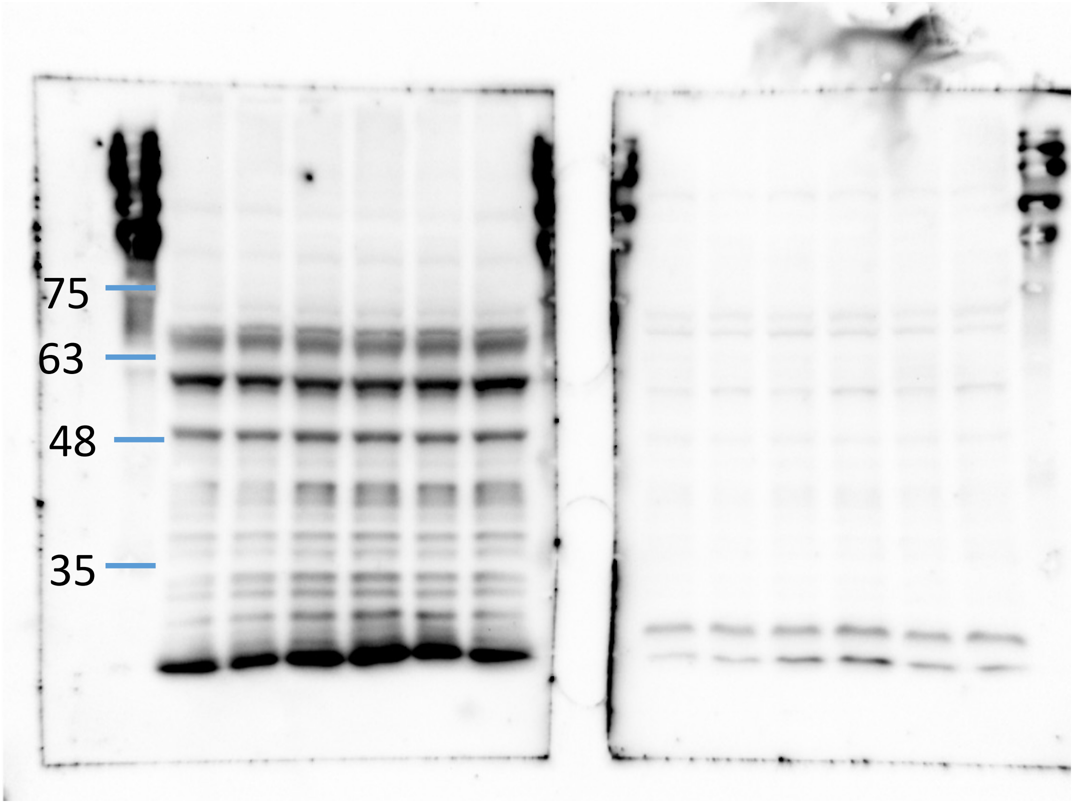

α-tubulin

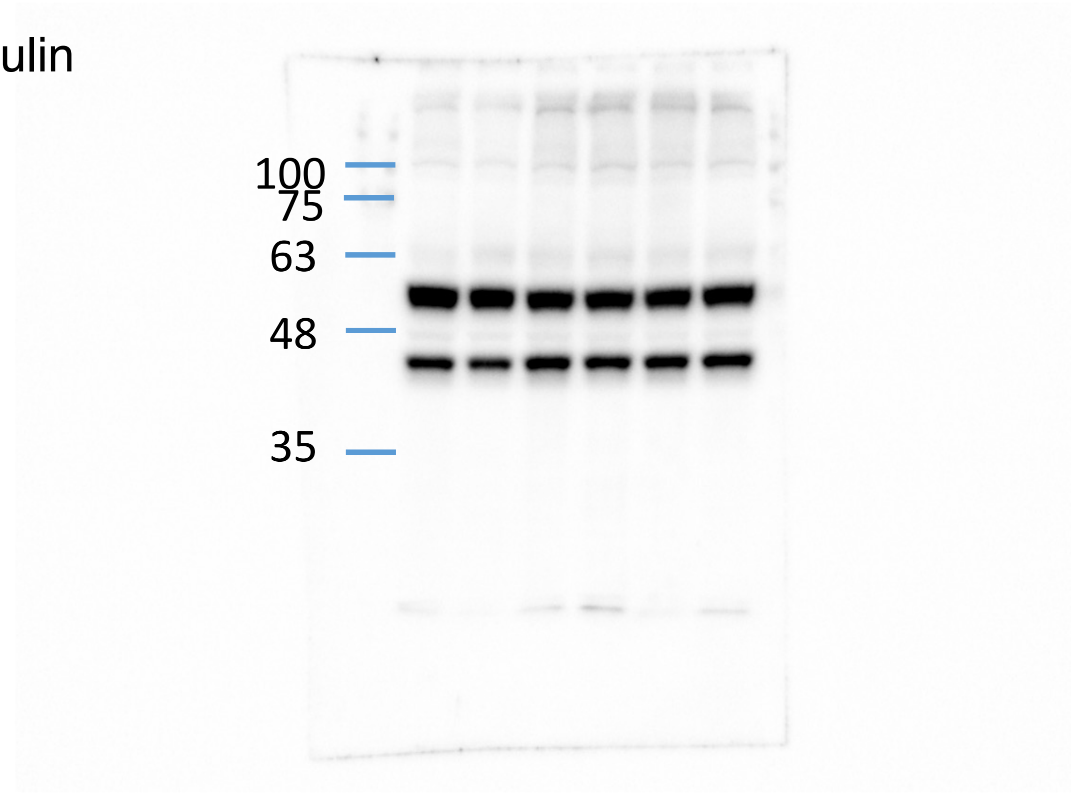

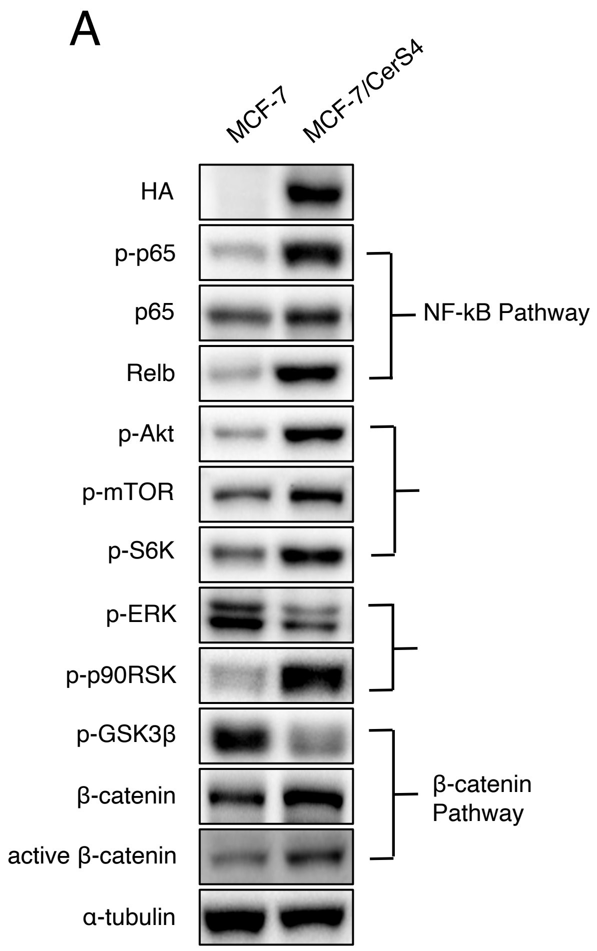
A
MCF-7
MCF-7/CerS4
HA
p-p65
p65
Relb
NF-kB Pathway
p-Akt
p-mTOR
p-S6K
p-ERK
p-p90RSK
p-GSK3β
β-catenin
active β-catenin
β-catenin Pathway
α-tubulin

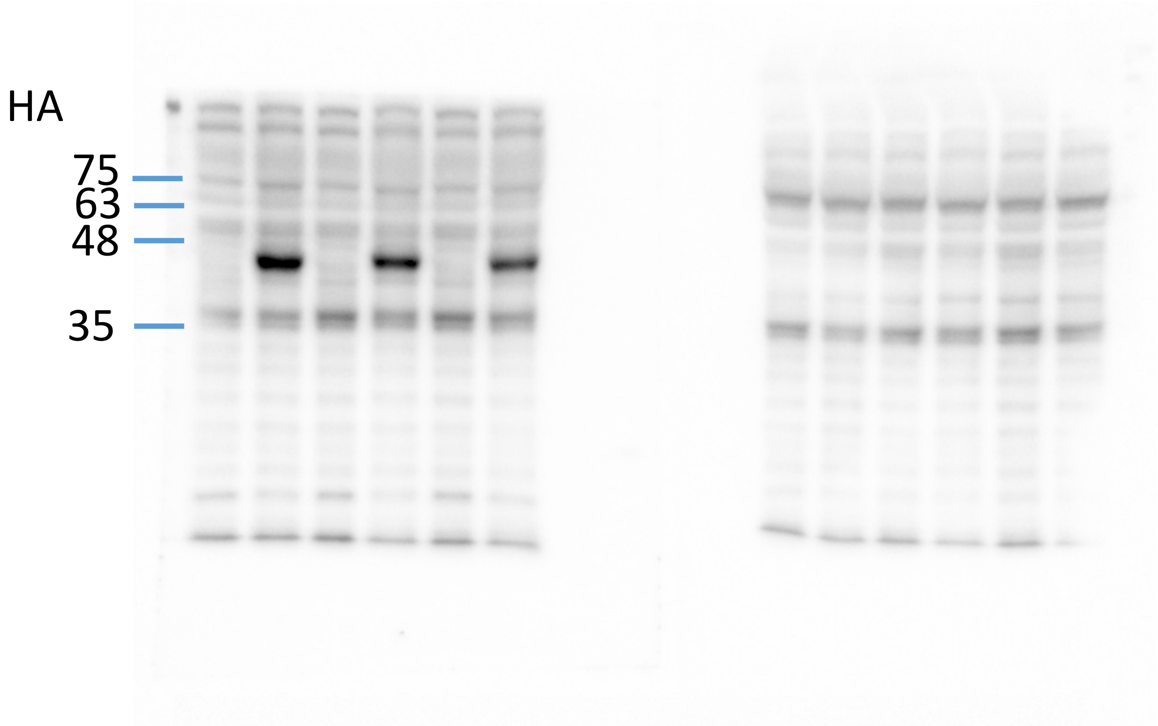
HA
75
63
48
35

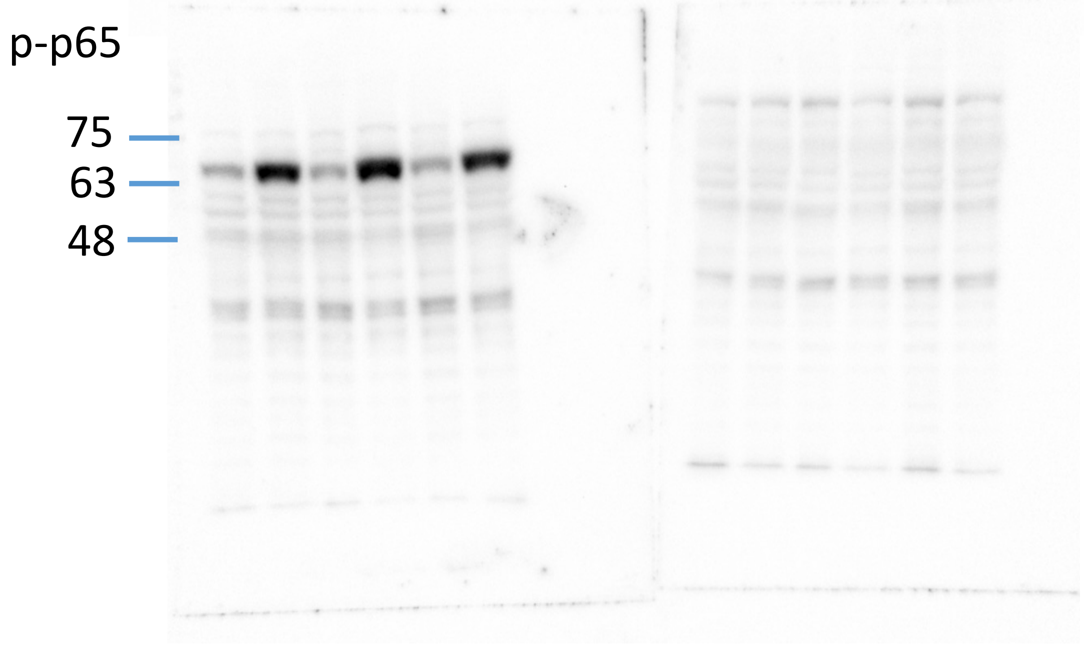
p-p65
75
63
48

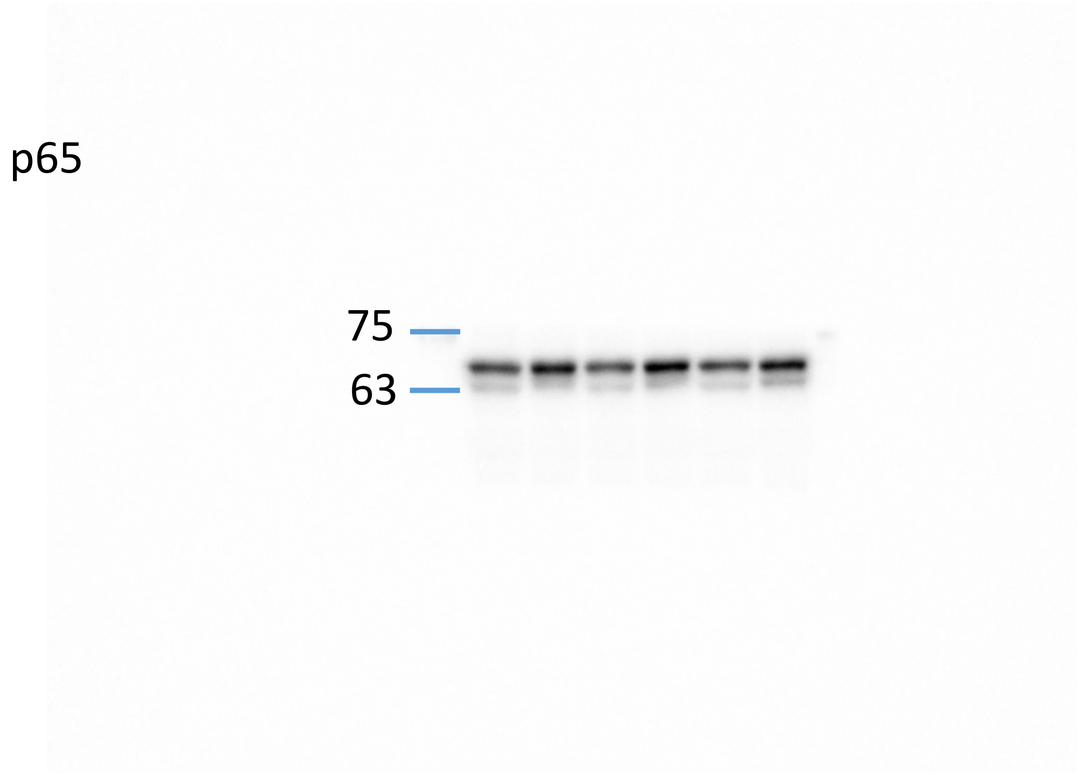
p65
75
63

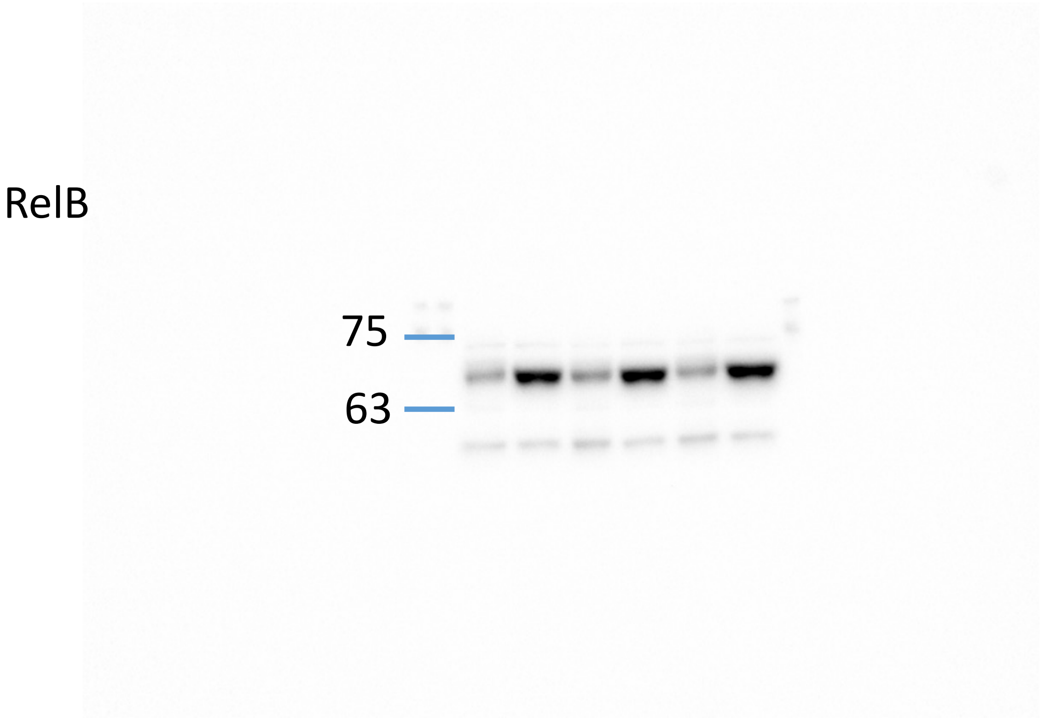
RelB
75
63

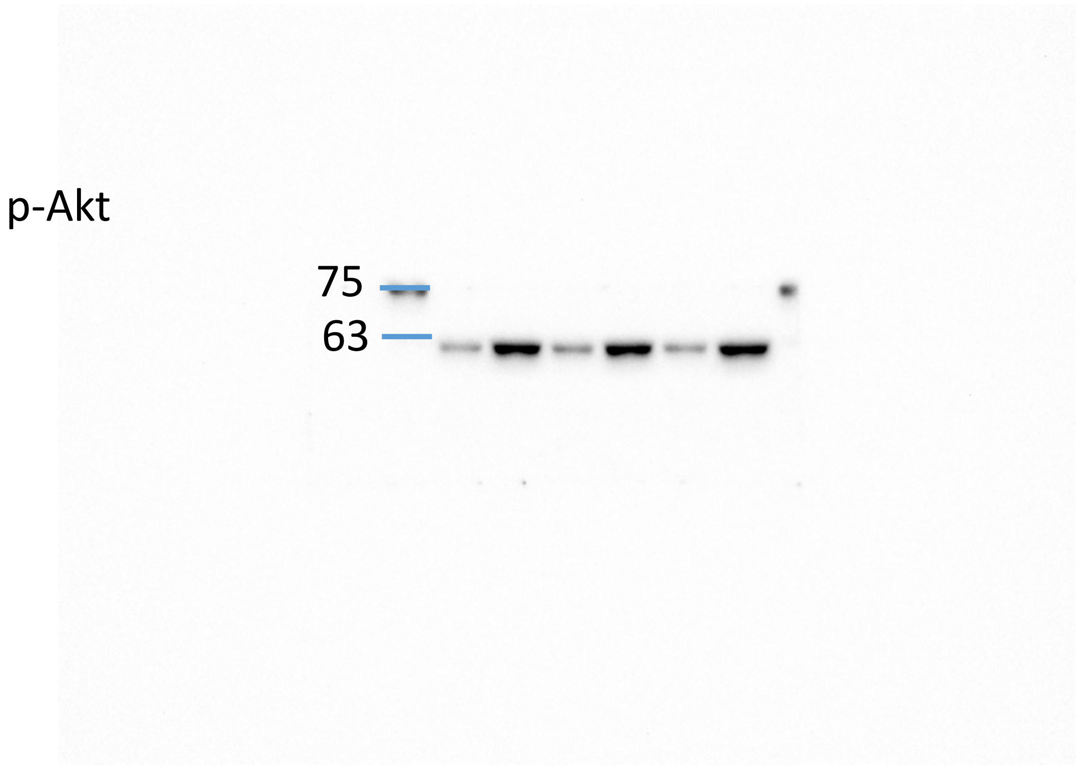
p-Akt
75
63

p-mTOR

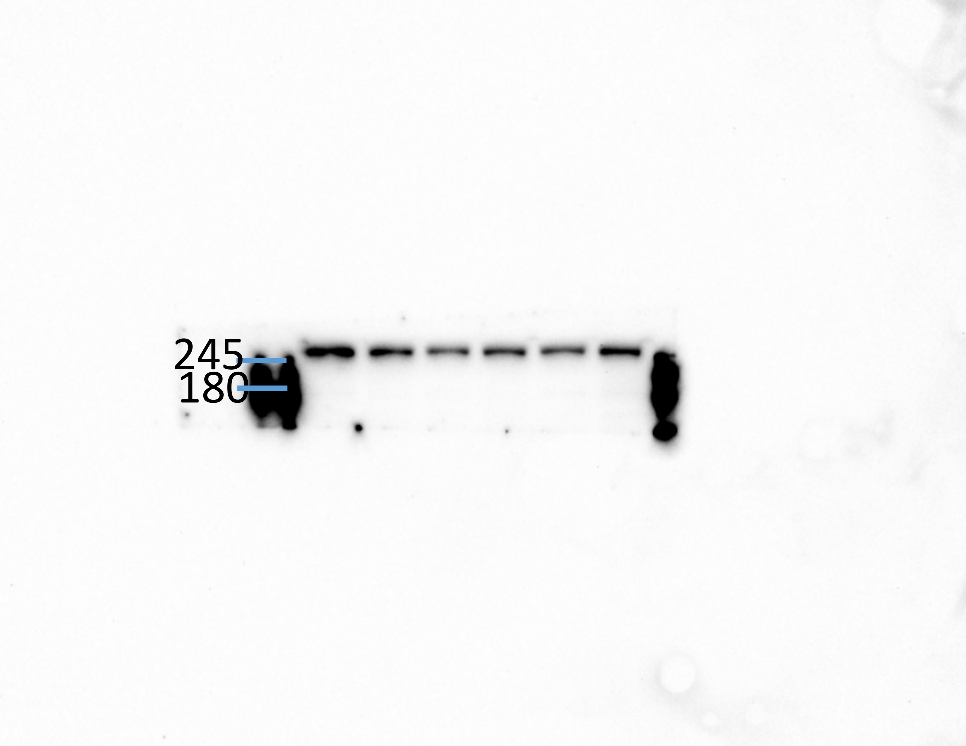

p-S6K

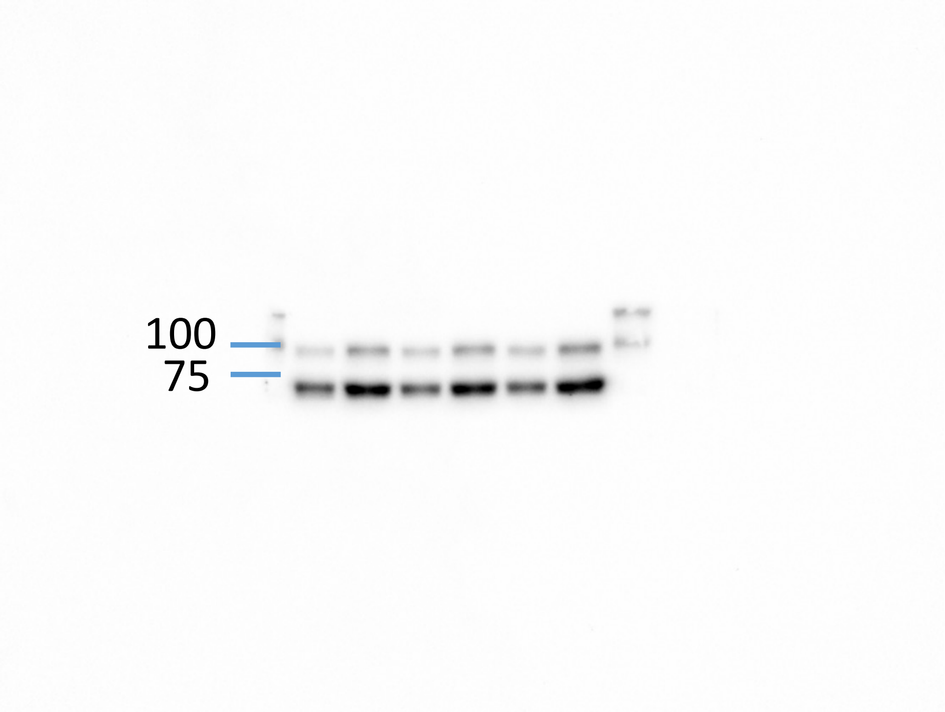

p-ERK

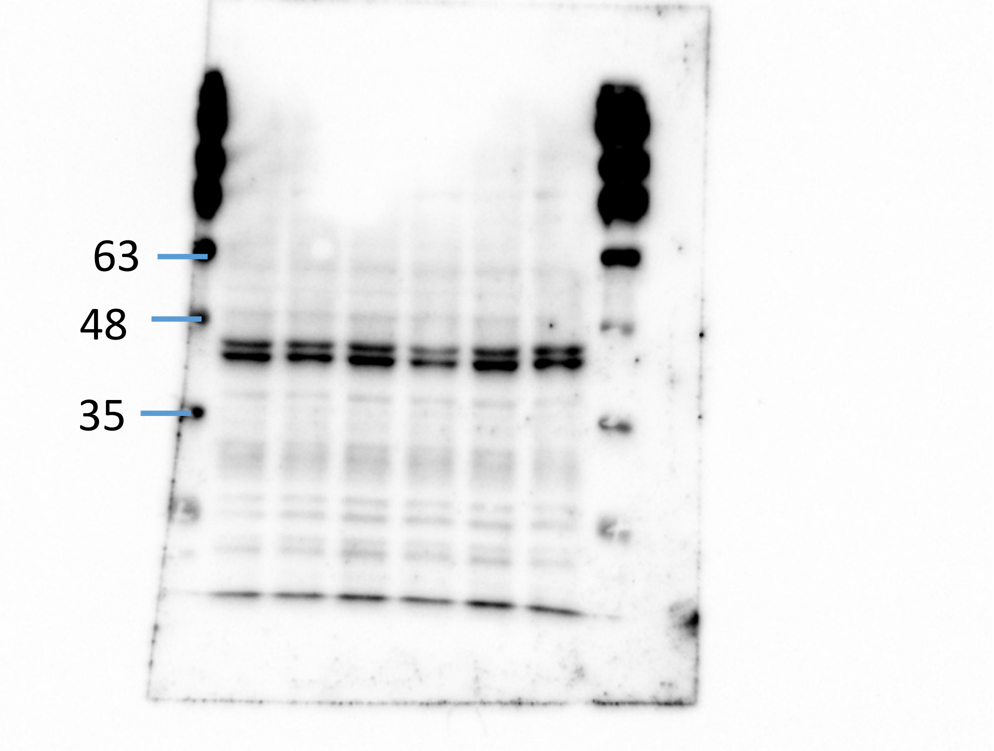

p-90RSK

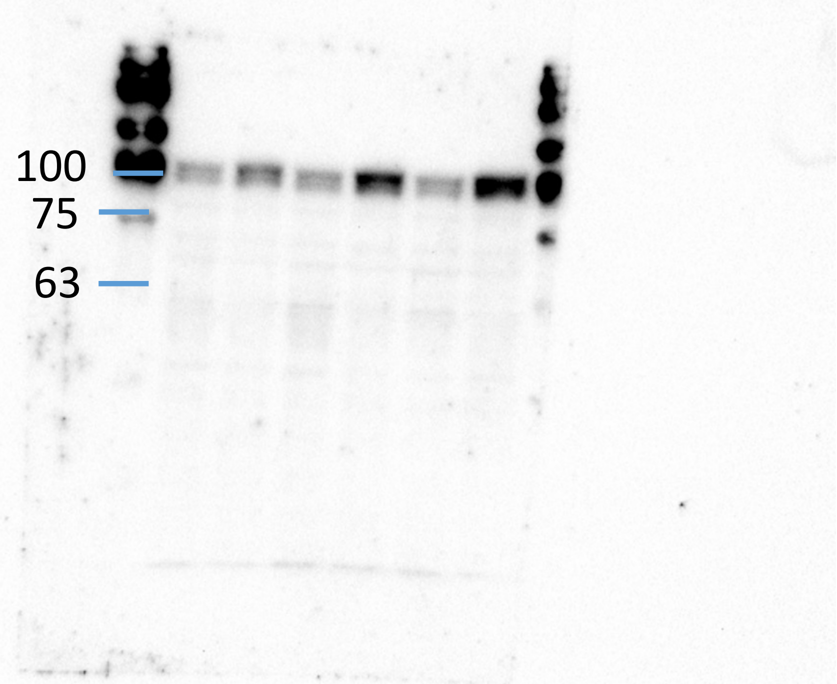

p-GSK3b

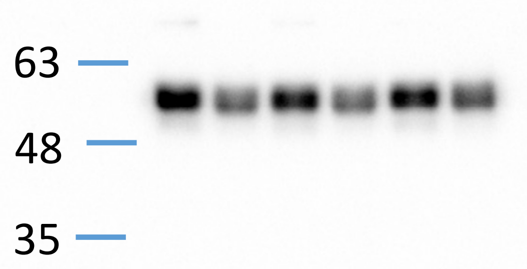

b-catenin

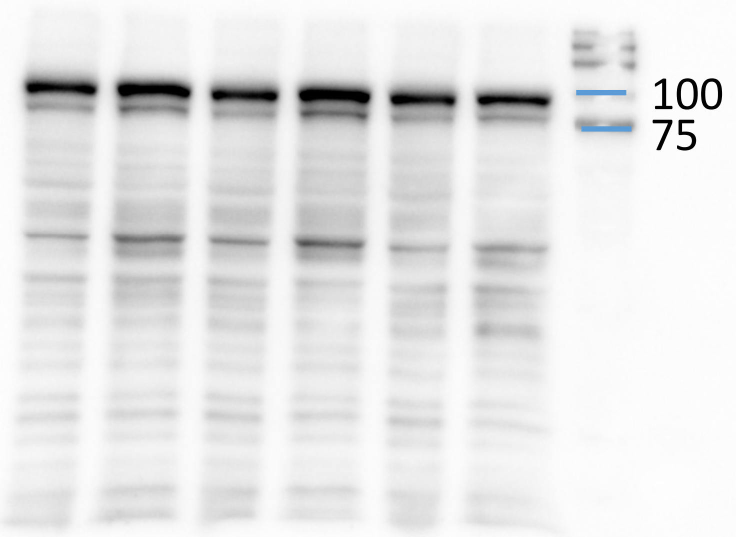

active b-catenin

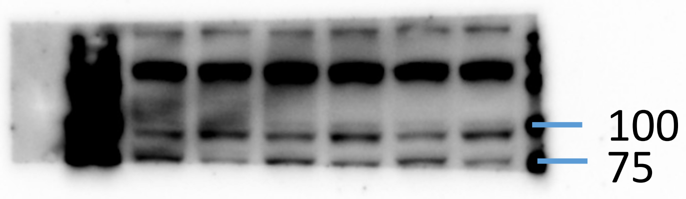

α-tubulin

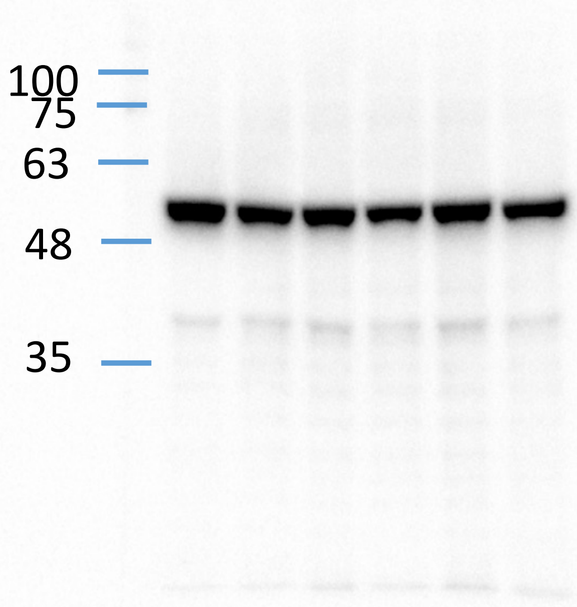

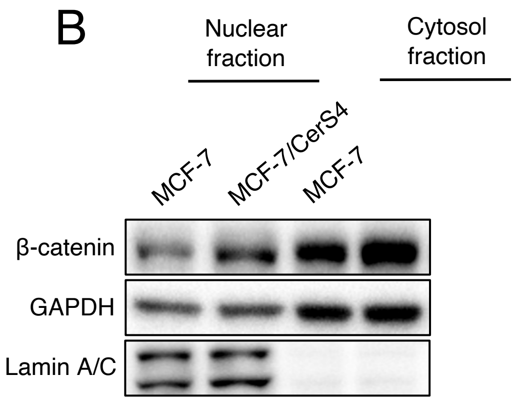

β-catenin

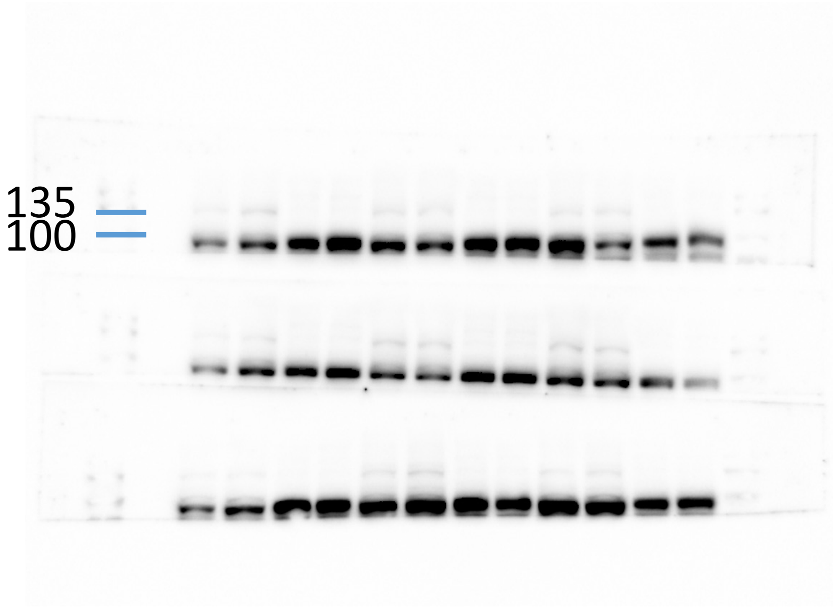

GAPDH

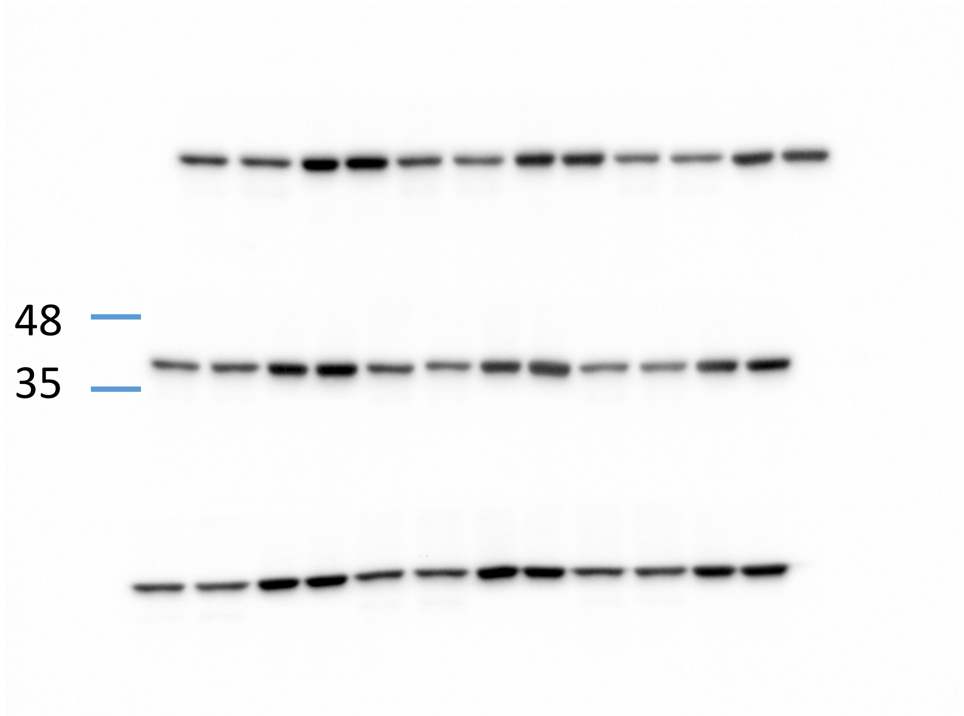

Lamin A/C

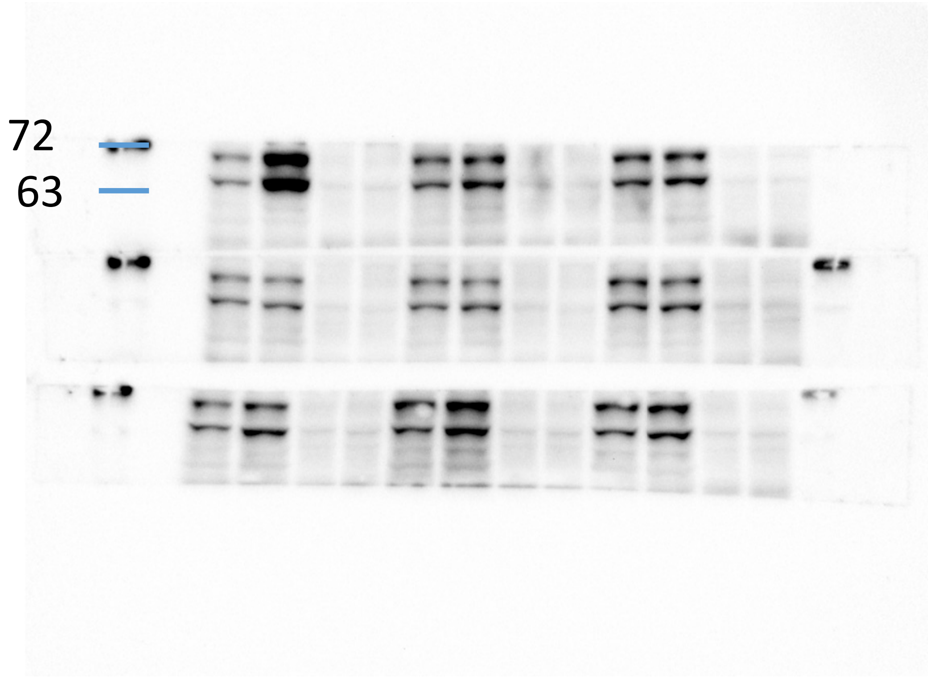

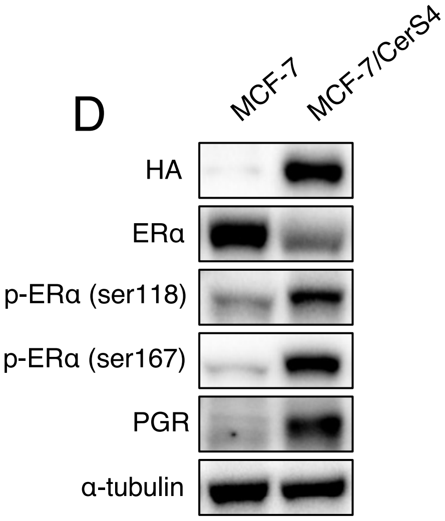
D
MCF-7
MCF-7/CerS4
HA
ERα
p-ERα (ser118)
p-ERα (ser167)
PGR
α-tubulin

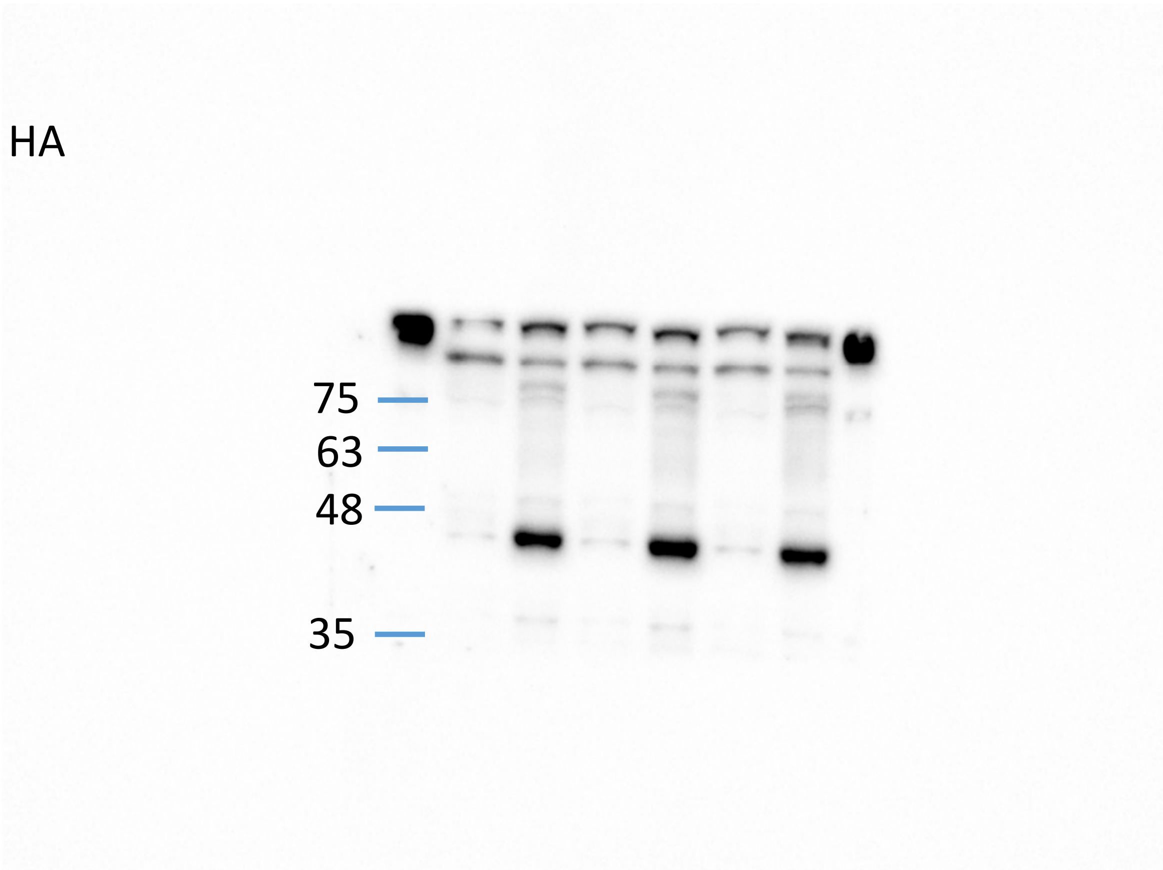
HA
75
63
48
35

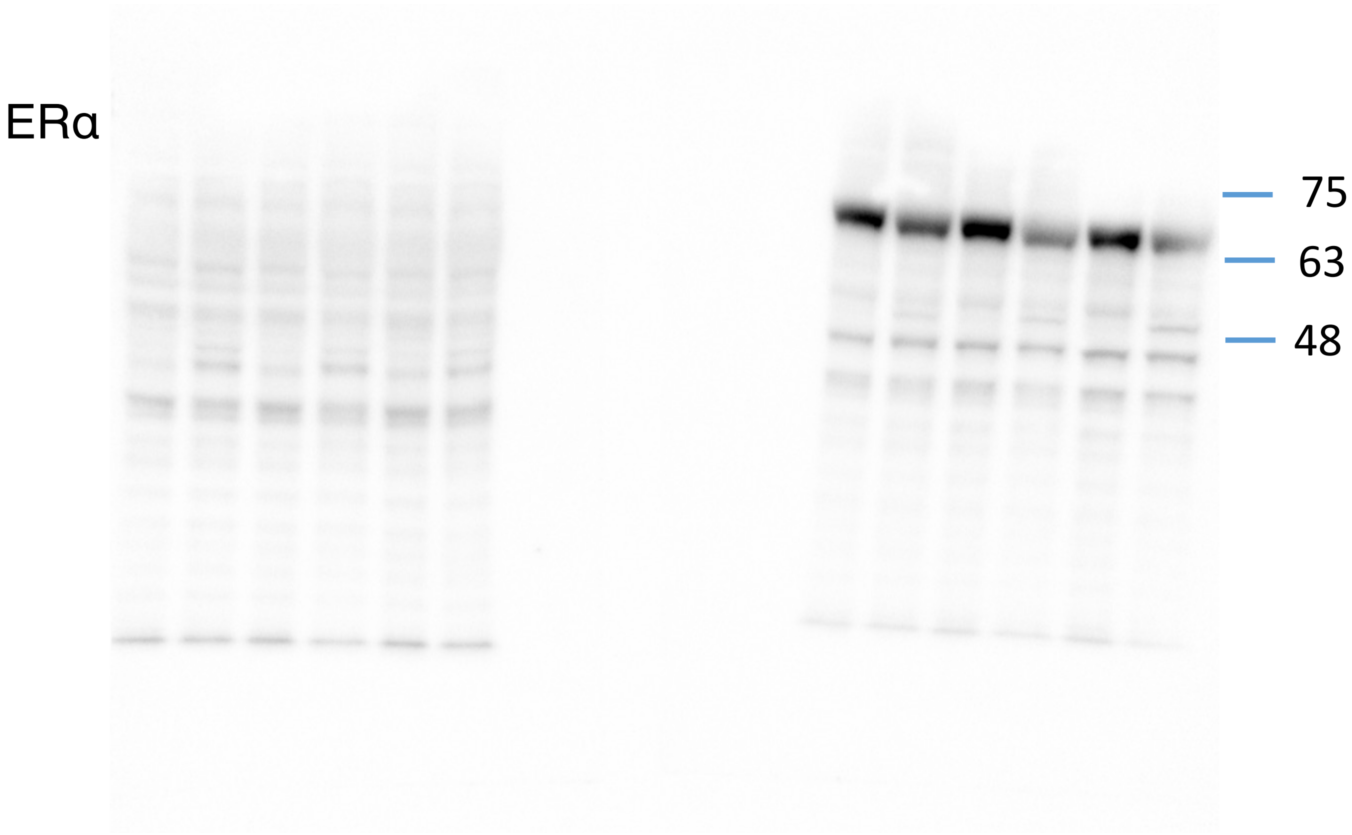
ERα
75
63
48

p-ERα (ser118)

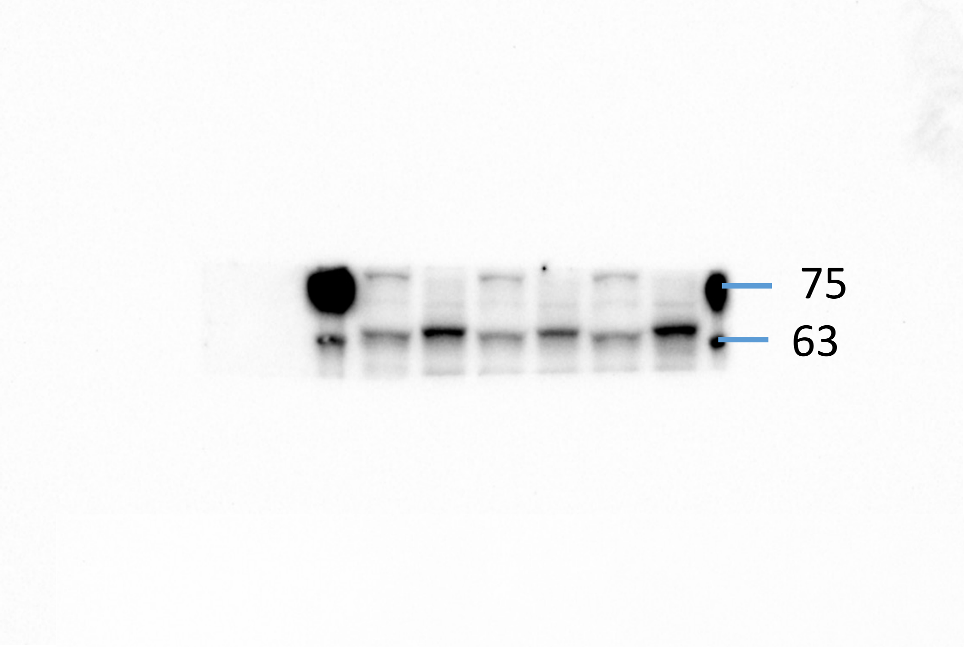

p-ERα (ser167)

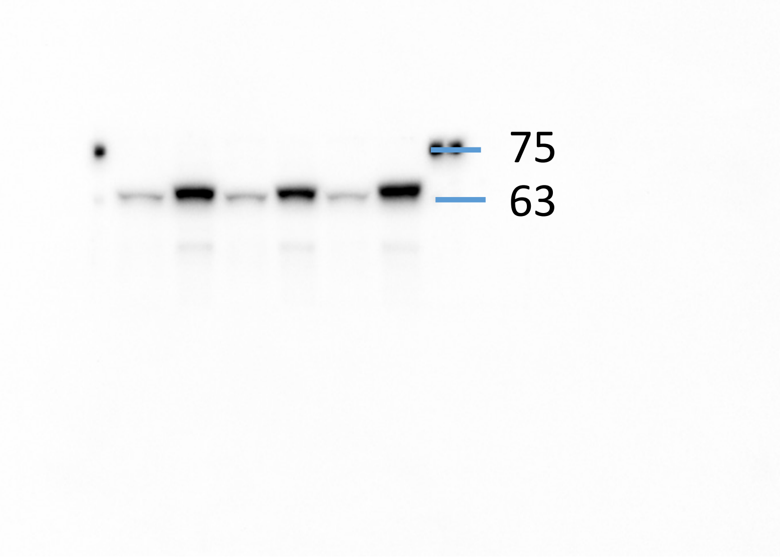

PGR

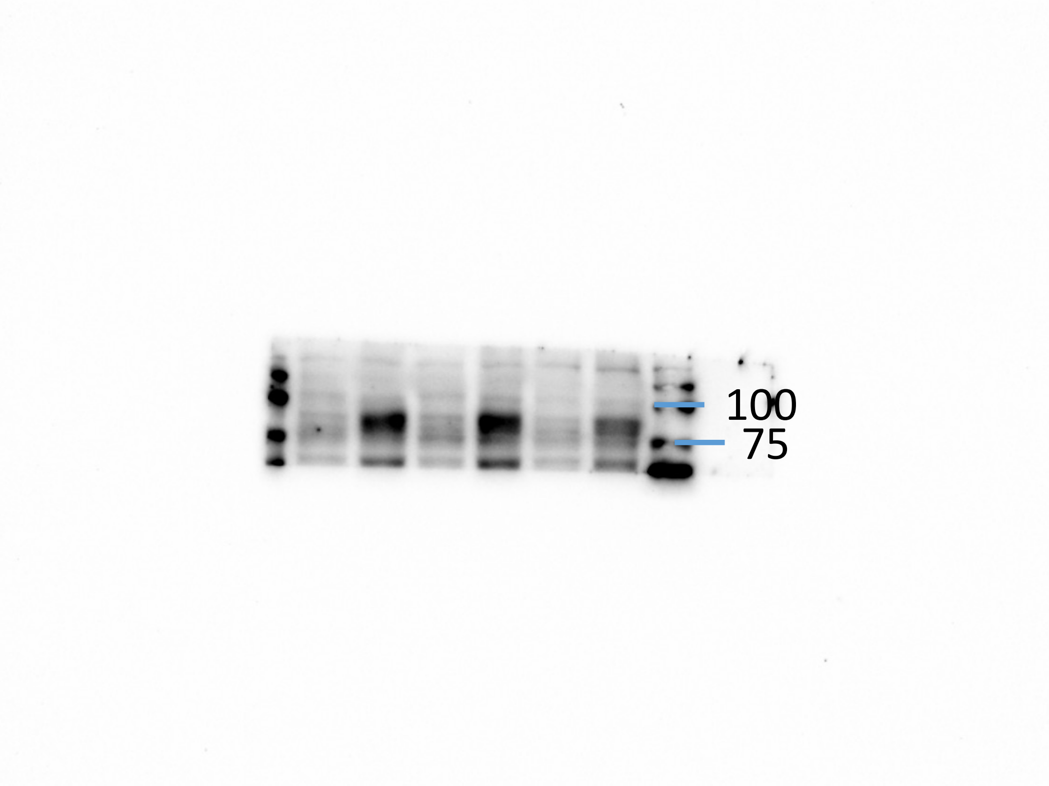

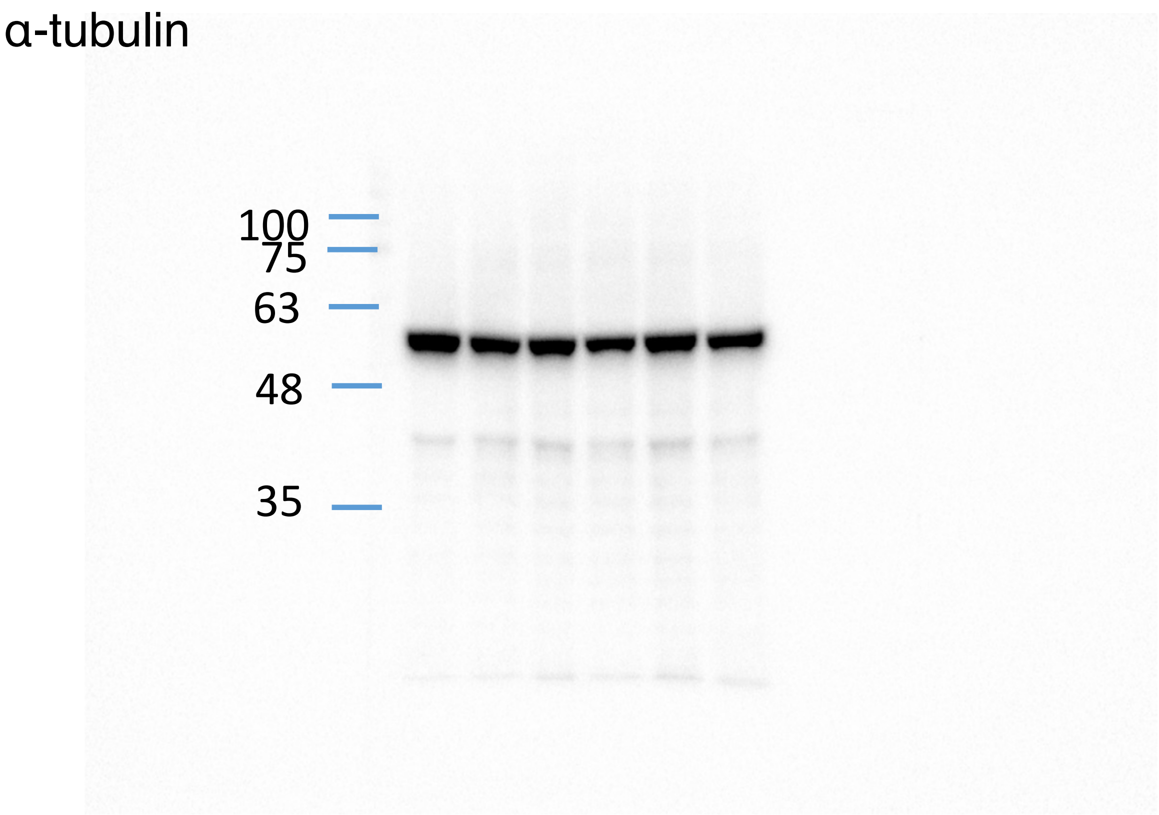
α-tubulin
100
75
63
48
35

A

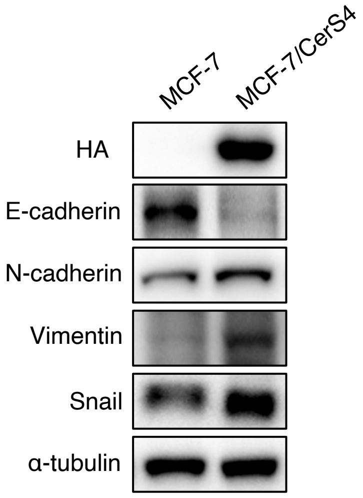

HA

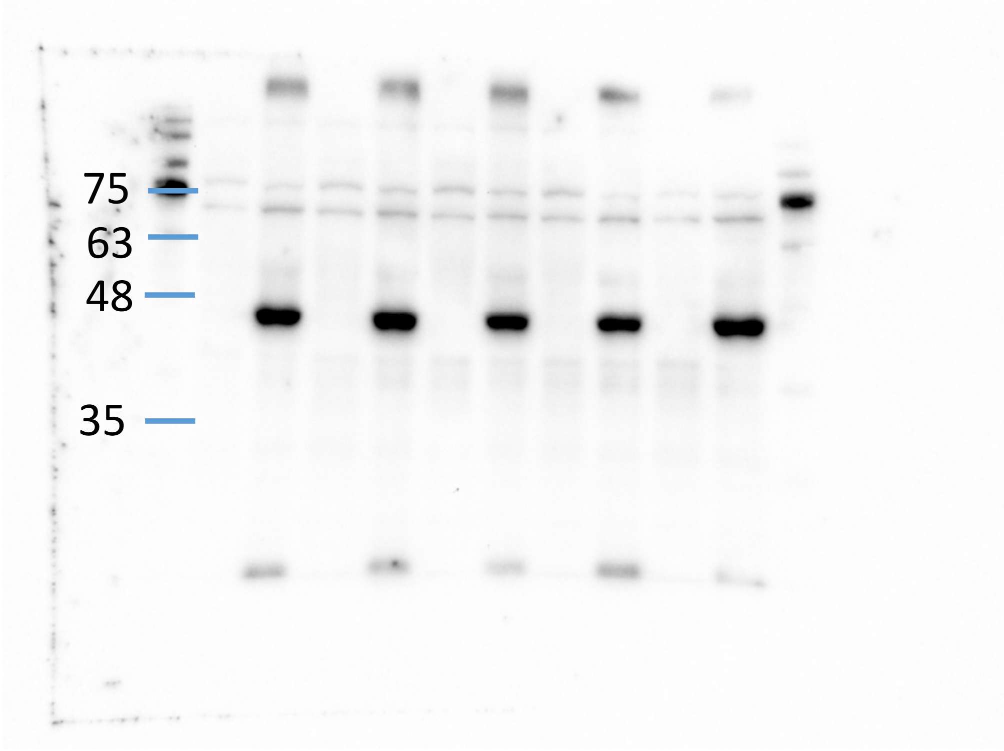

E-cadherin

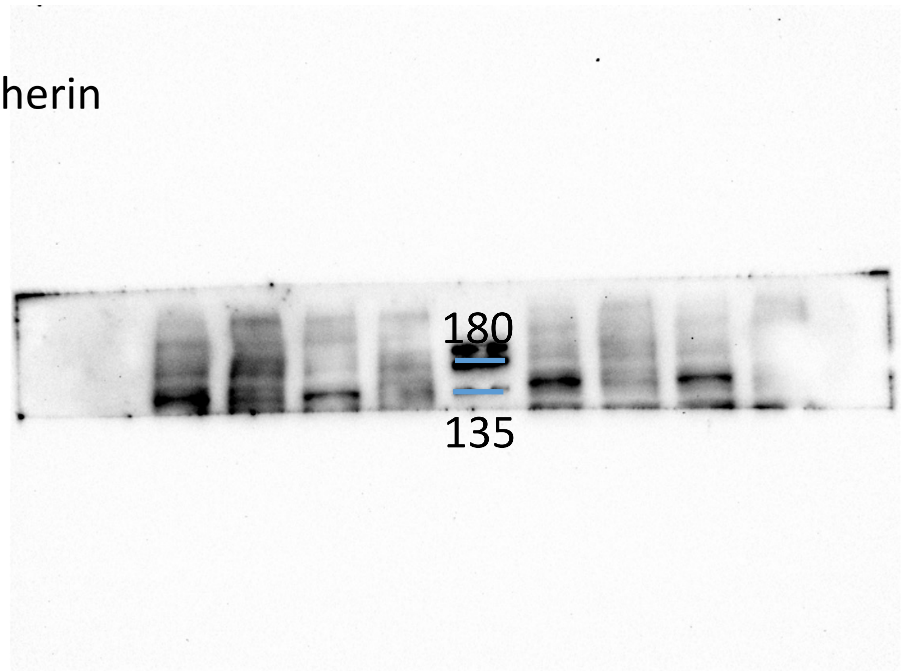

N-cadherin

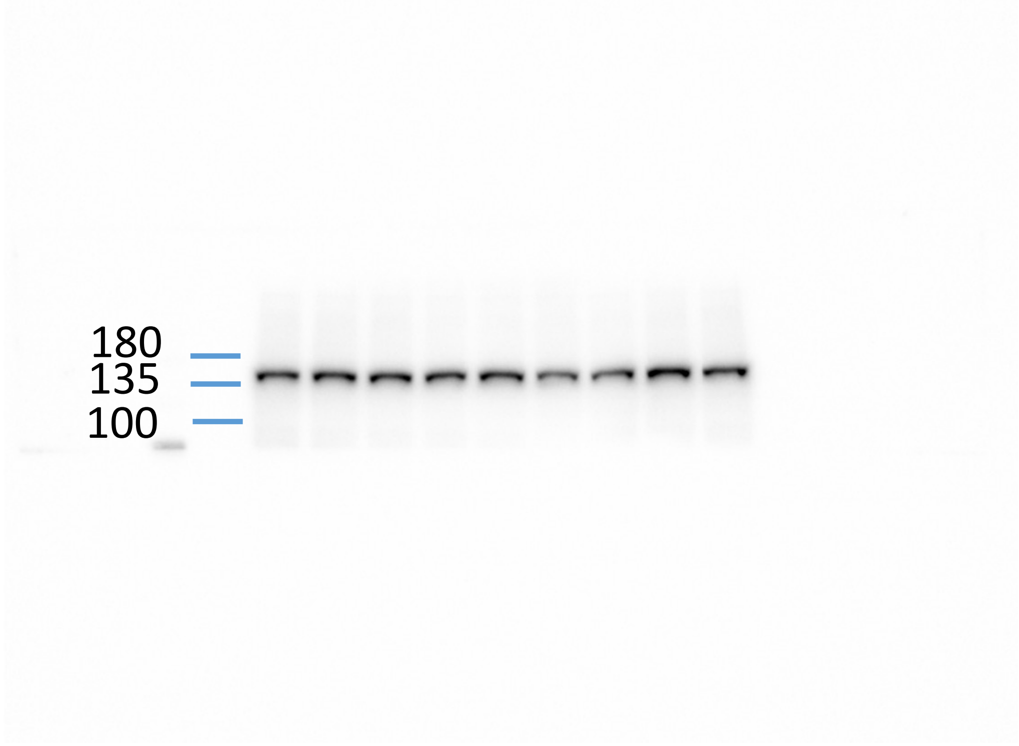

Vimentin

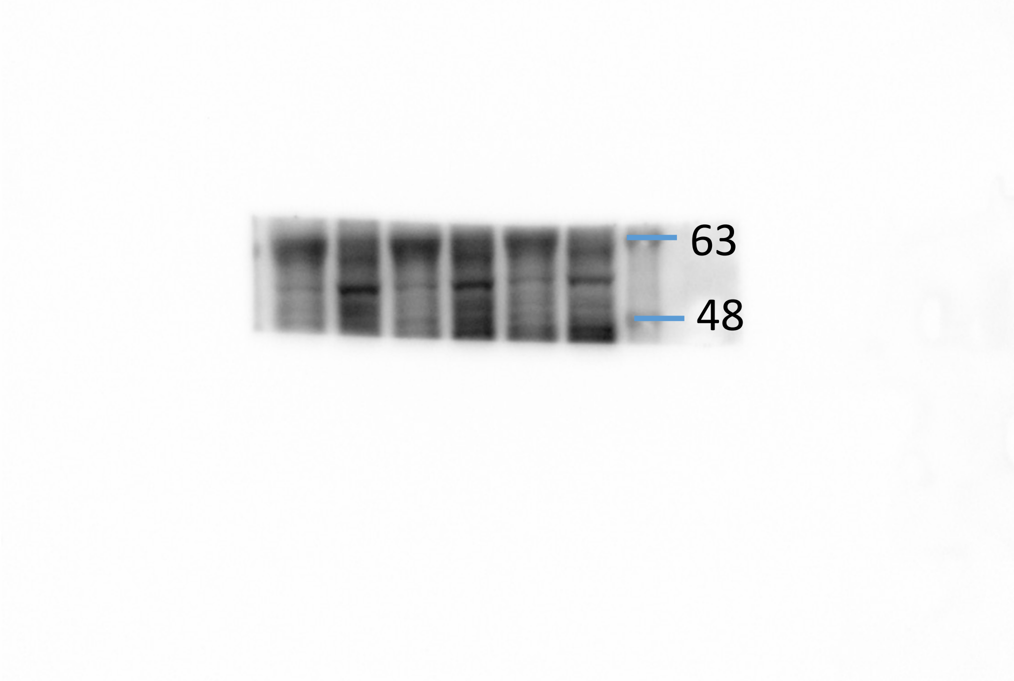

Snail

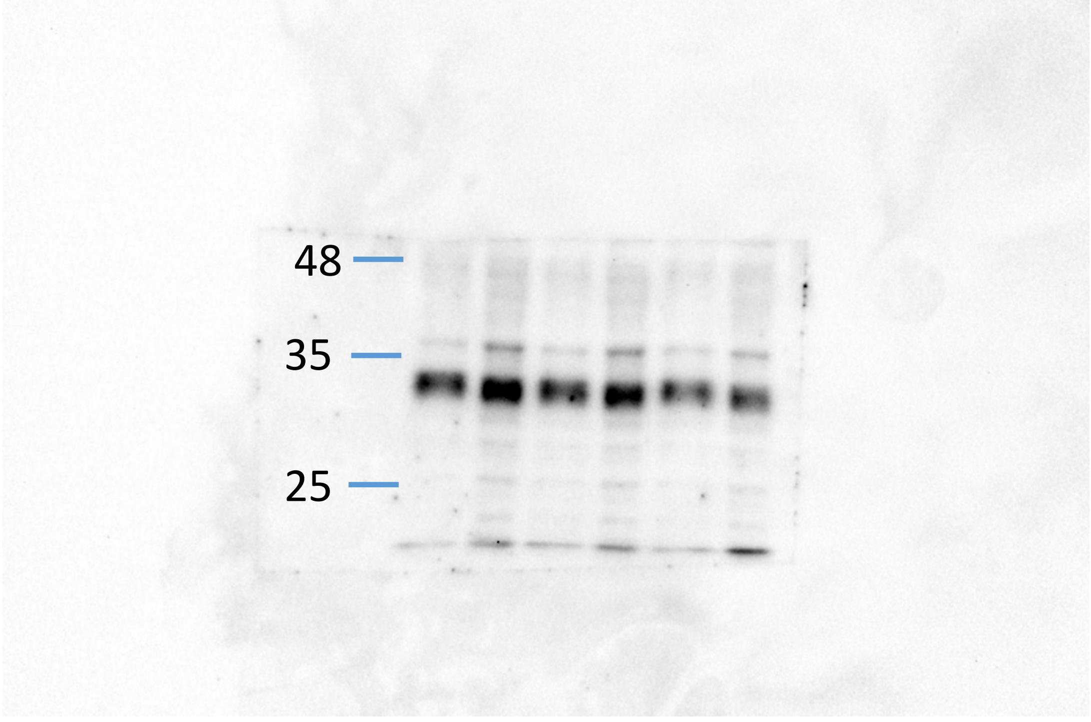

α-tubulin

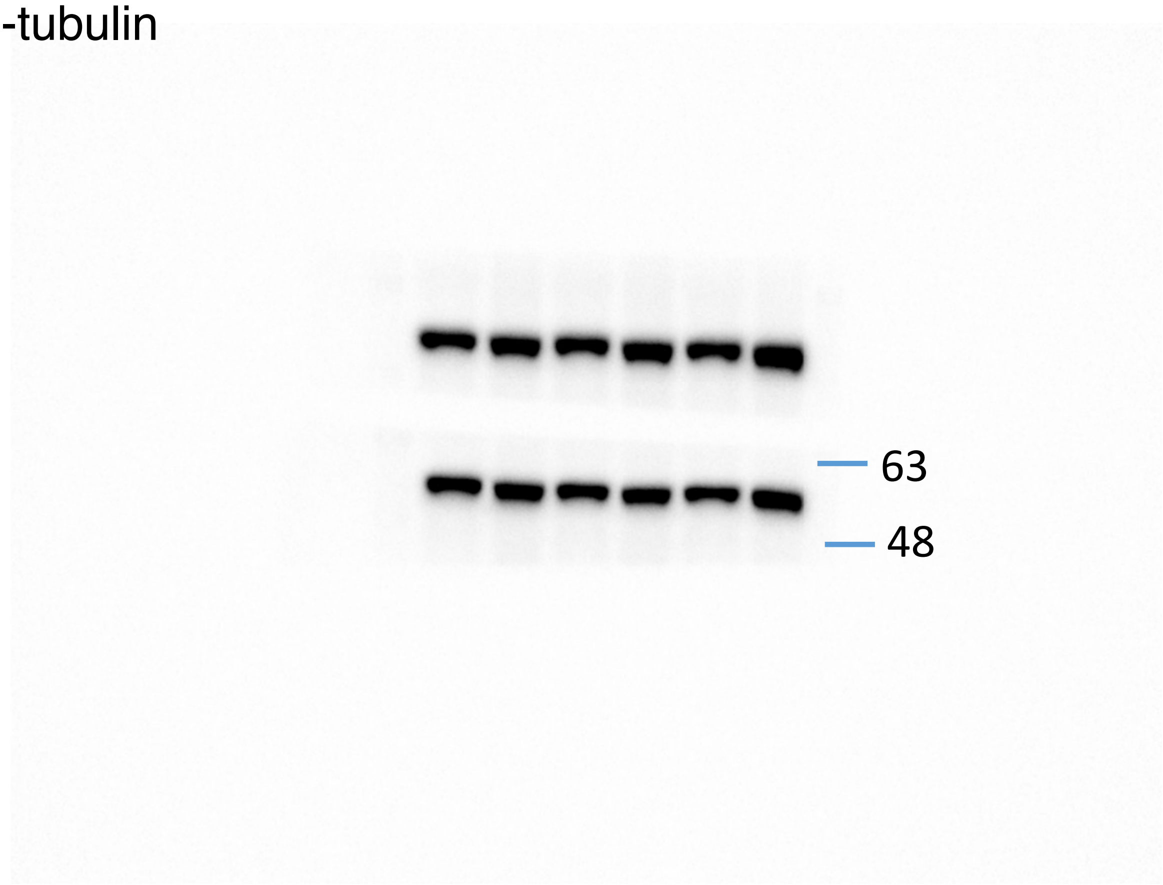

A

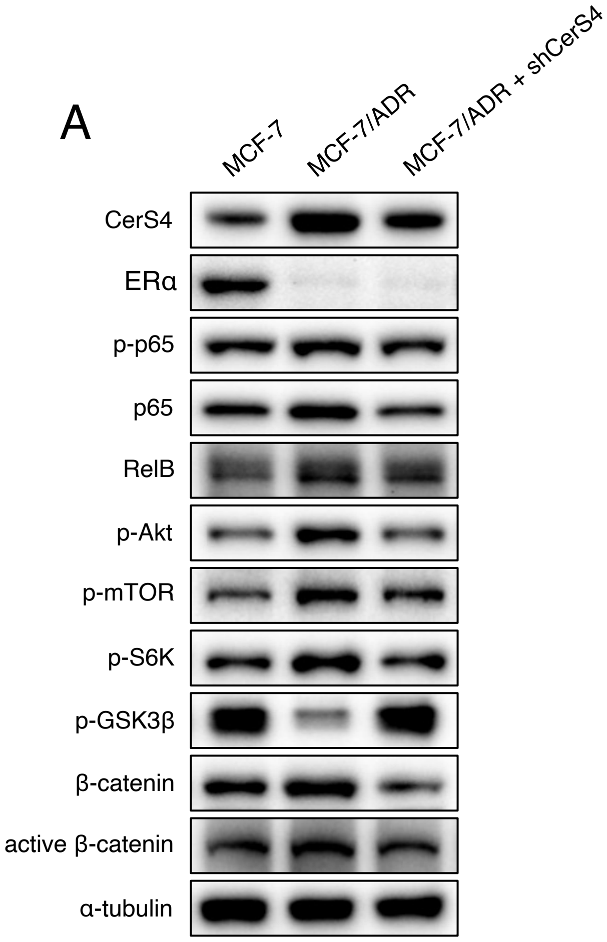
MCF-7
MCF-7/ADR
MCF-7/ADR + shCerS4
CerS4
ERα
p-p65
p65
RelB
p-Akt
p-mTOR
p-S6K
p-GSK3β
β-catenin
active β-catenin
α-tubulin

CerS4

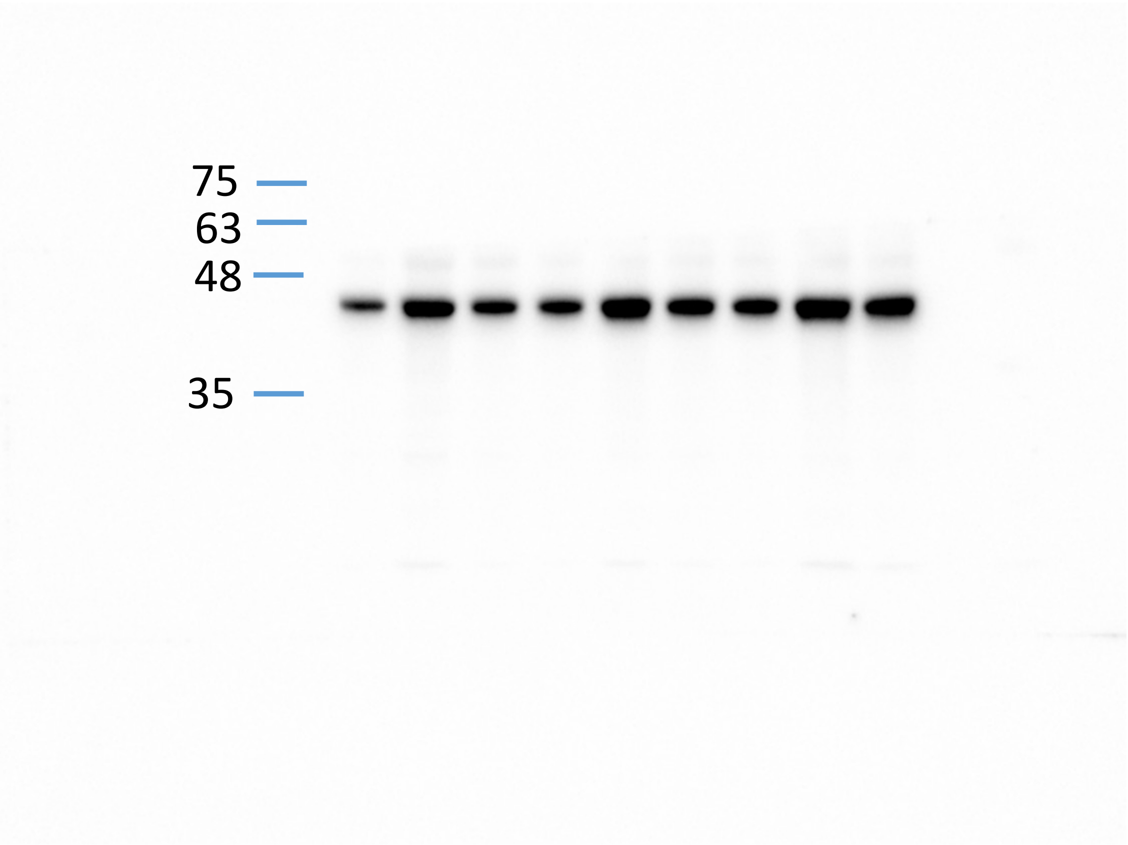
75
63
48
35

ERα

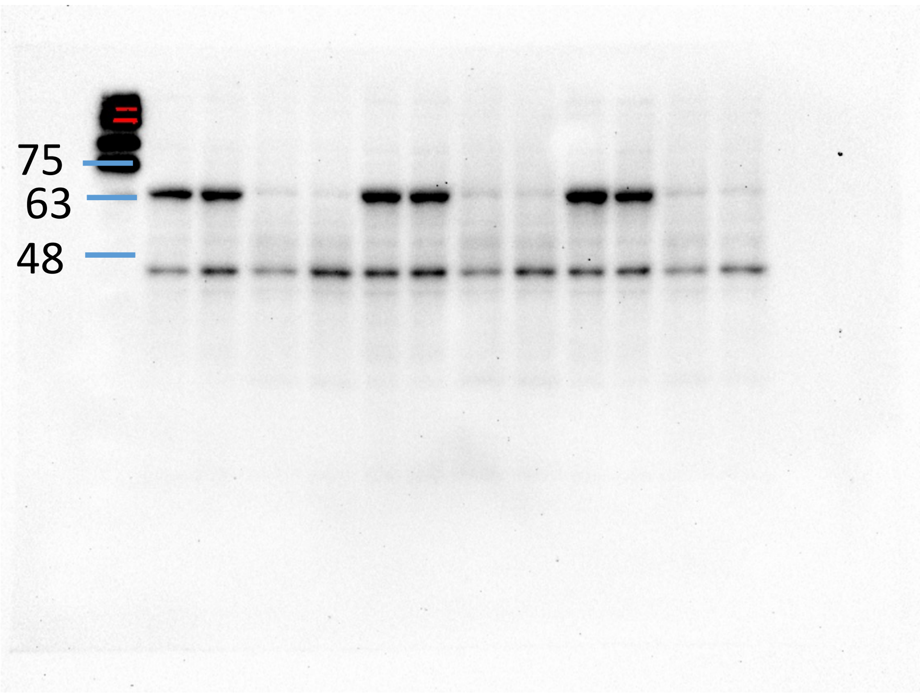
75
63
48

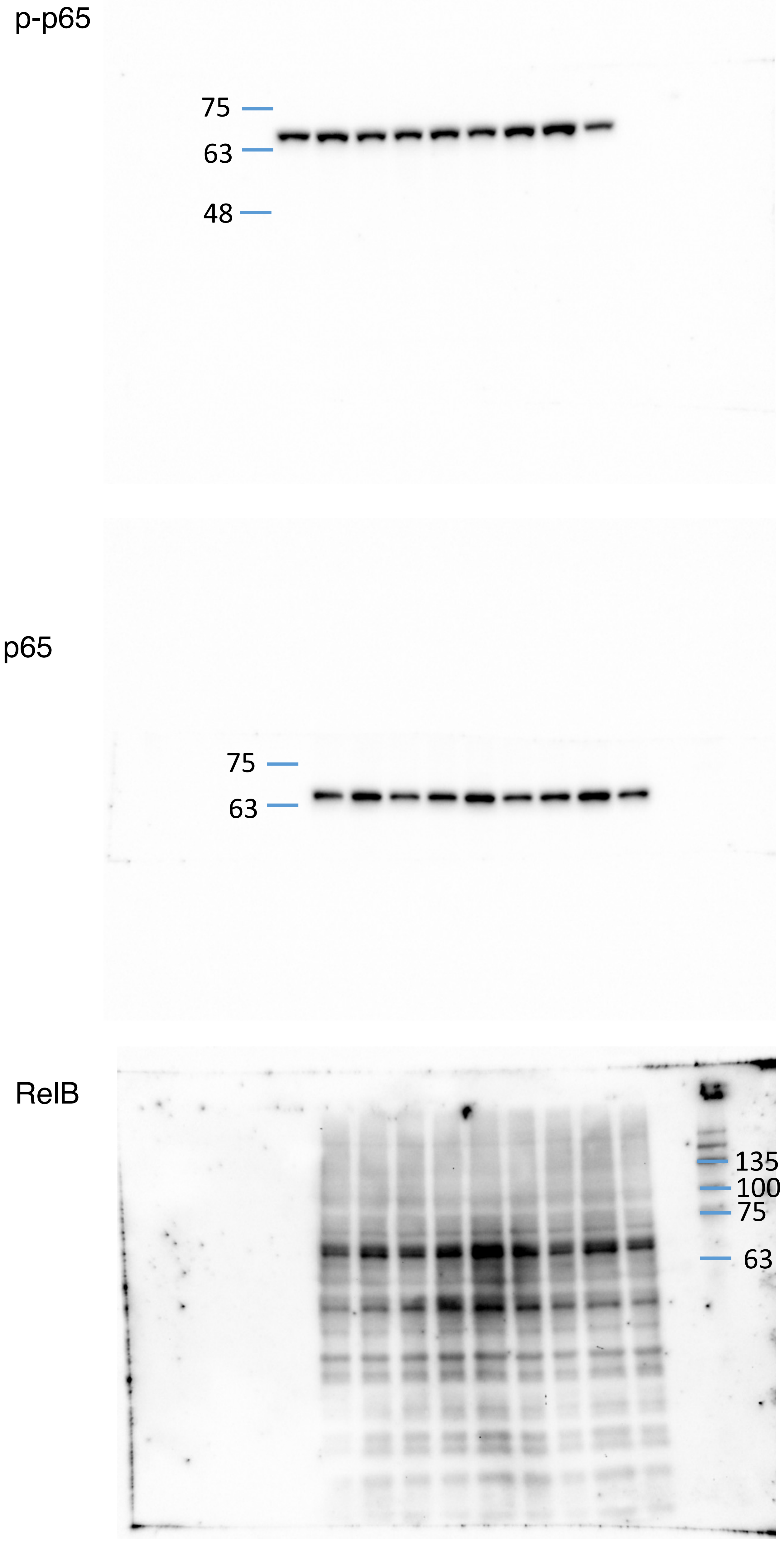
p-p65
75
63
48
p65
75
63
RelB
135
100
75
63

p-Akt

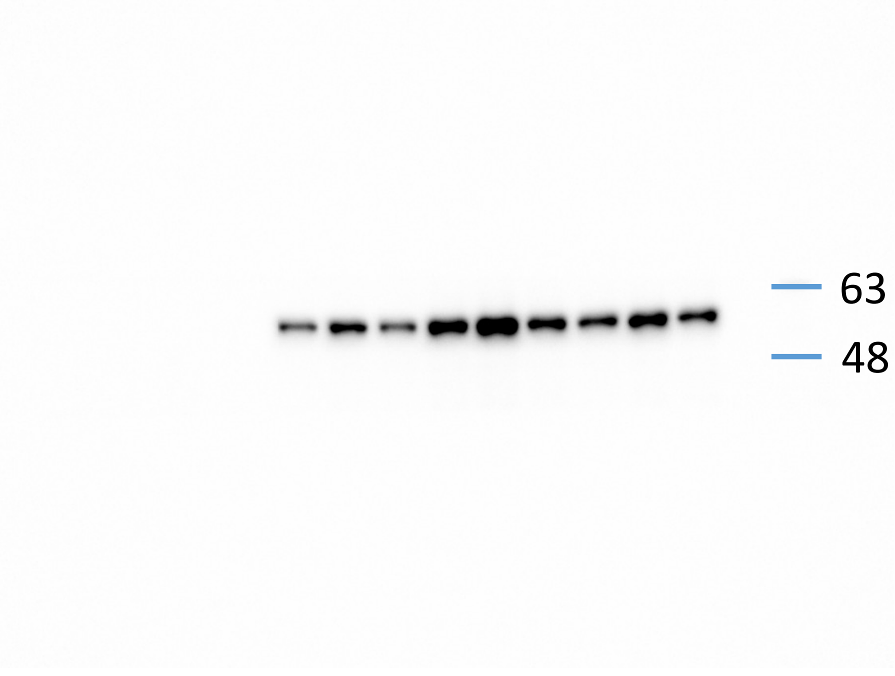

p-mTOR

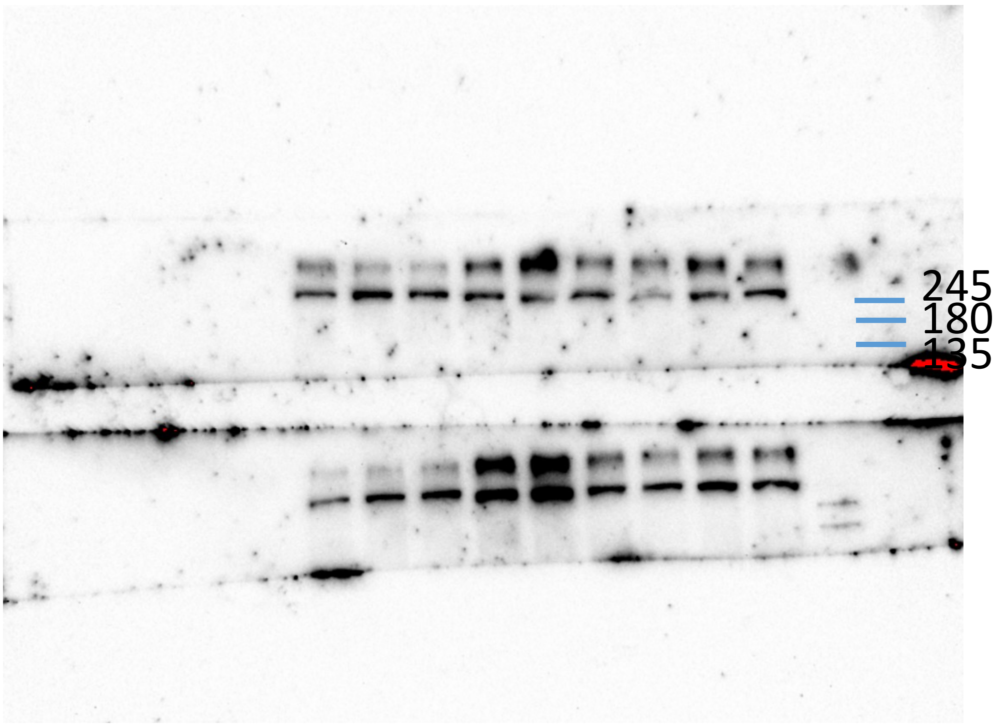

p-S6K

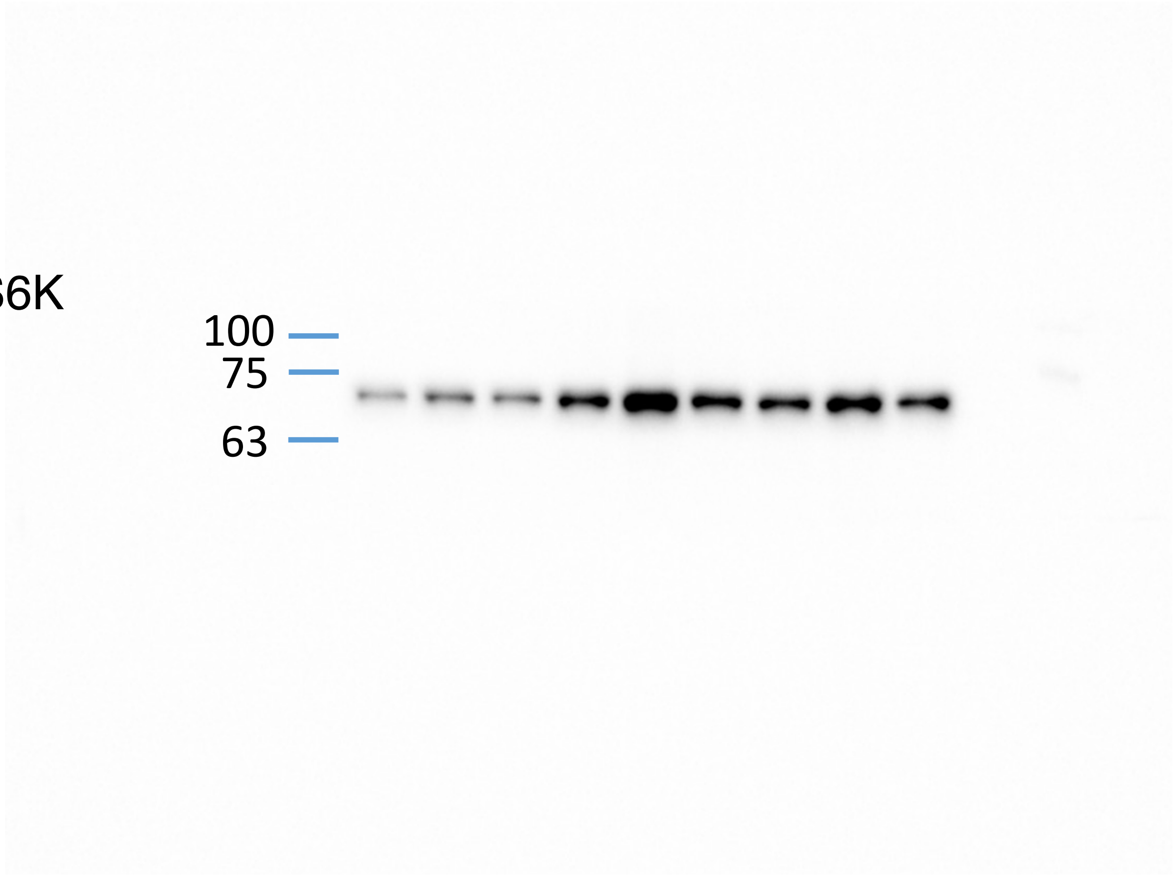

p-GSK3β

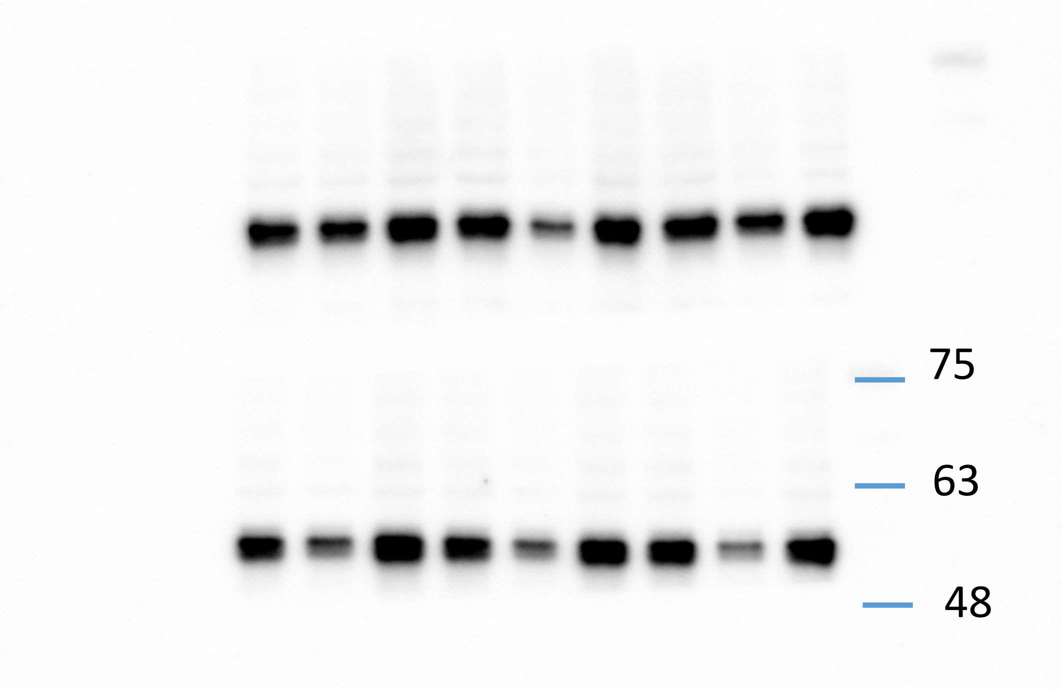

β-catenin

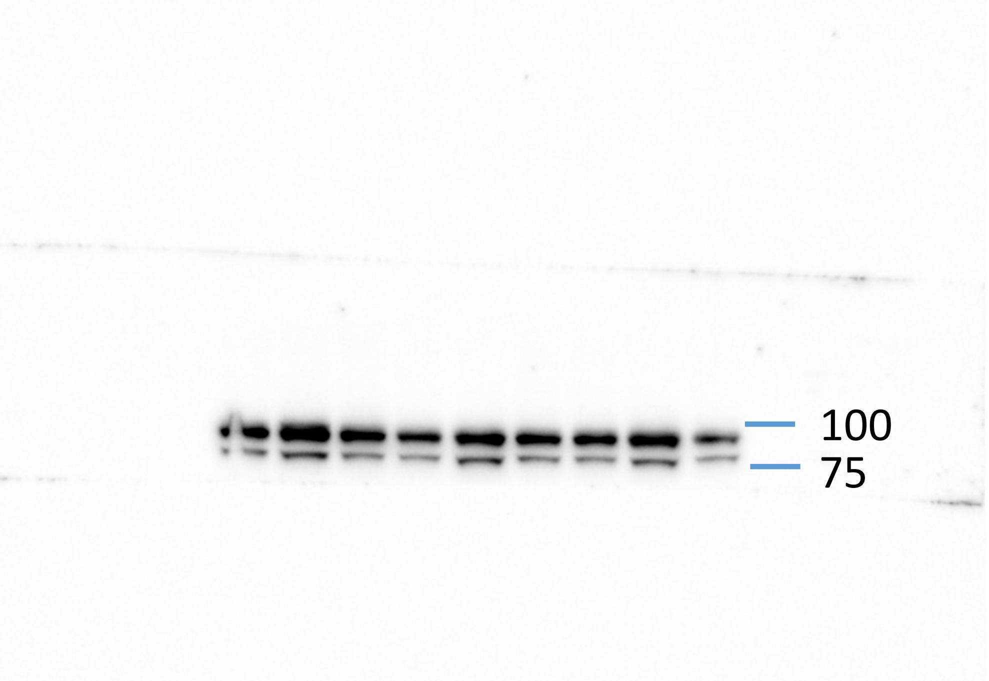

active β-catenin

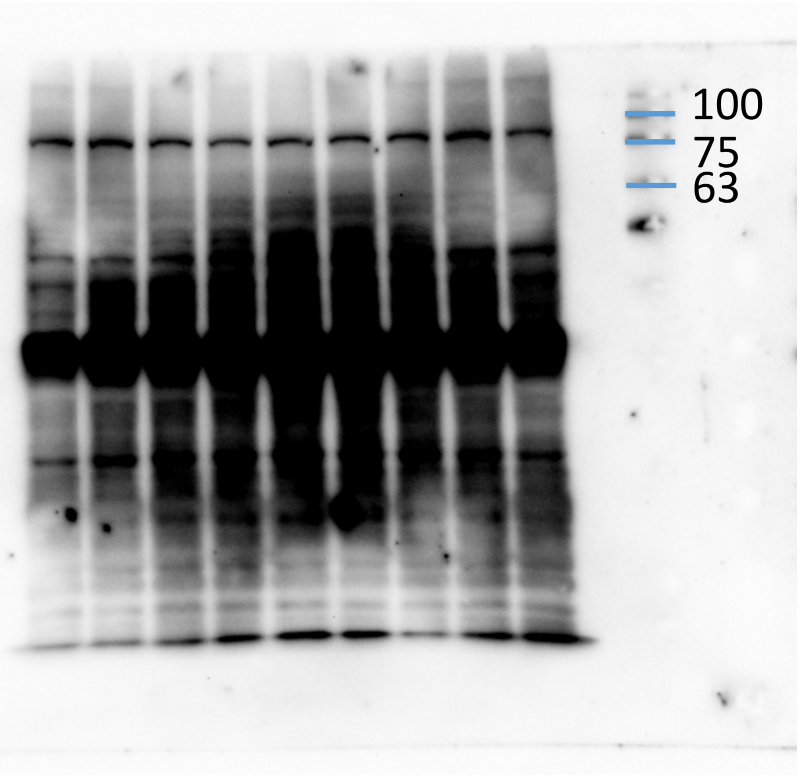

α-tubulin

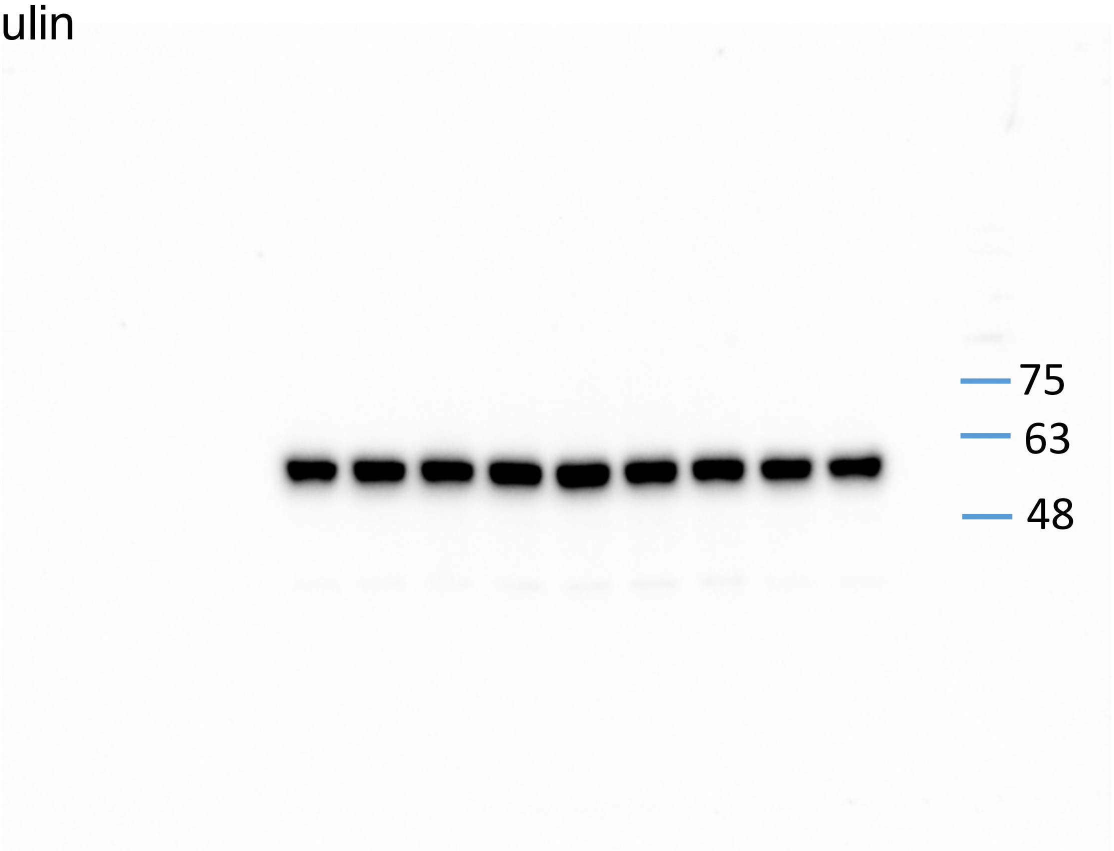

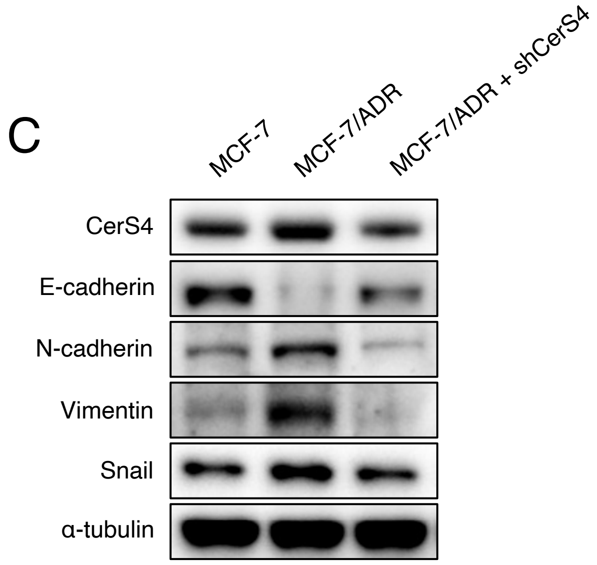

C
MCF-7
MCF-7/ADR
MCF-7/ADR + shCerS4
CerS4
E-cadherin
N-cadherin
Vimentin
Snail
α-tubulin

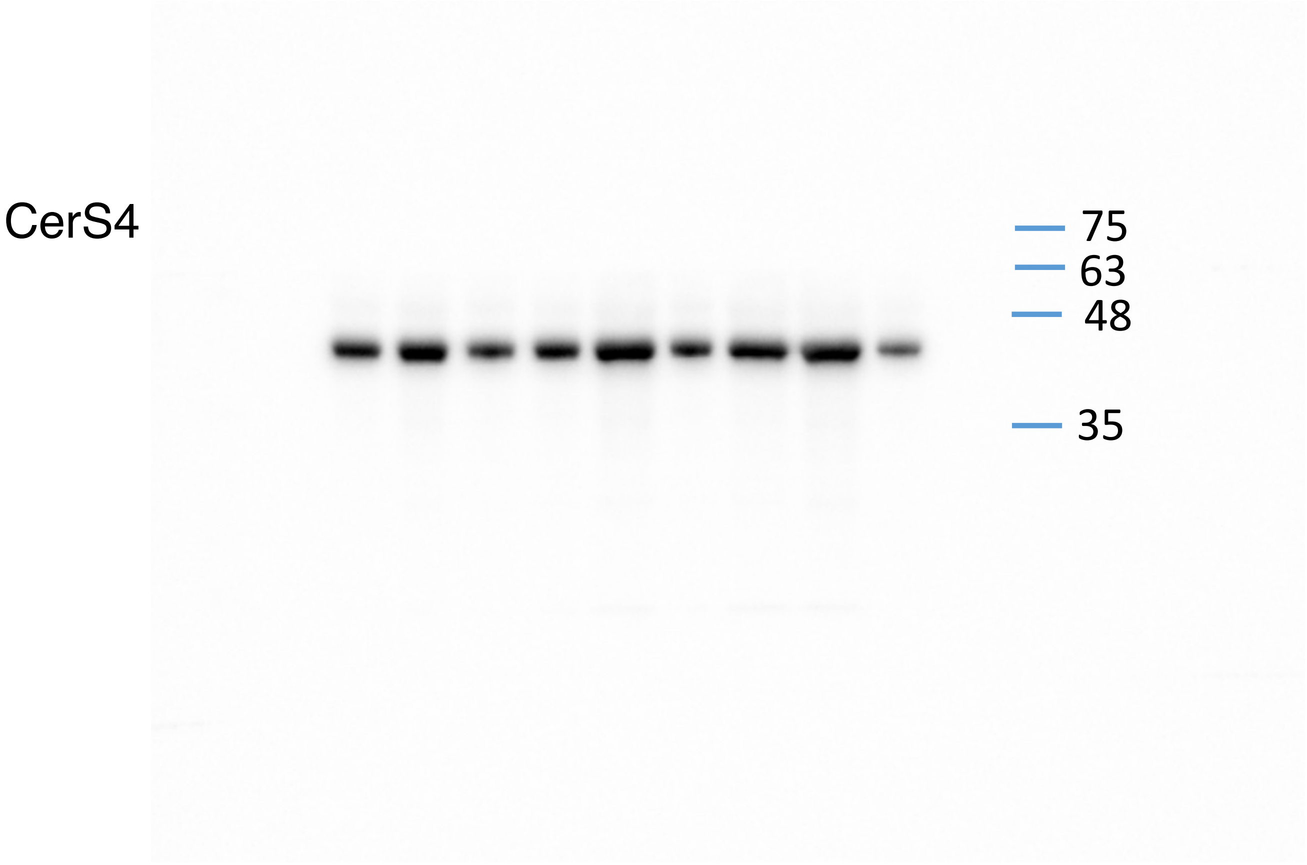

CerS4
75
63
48
35

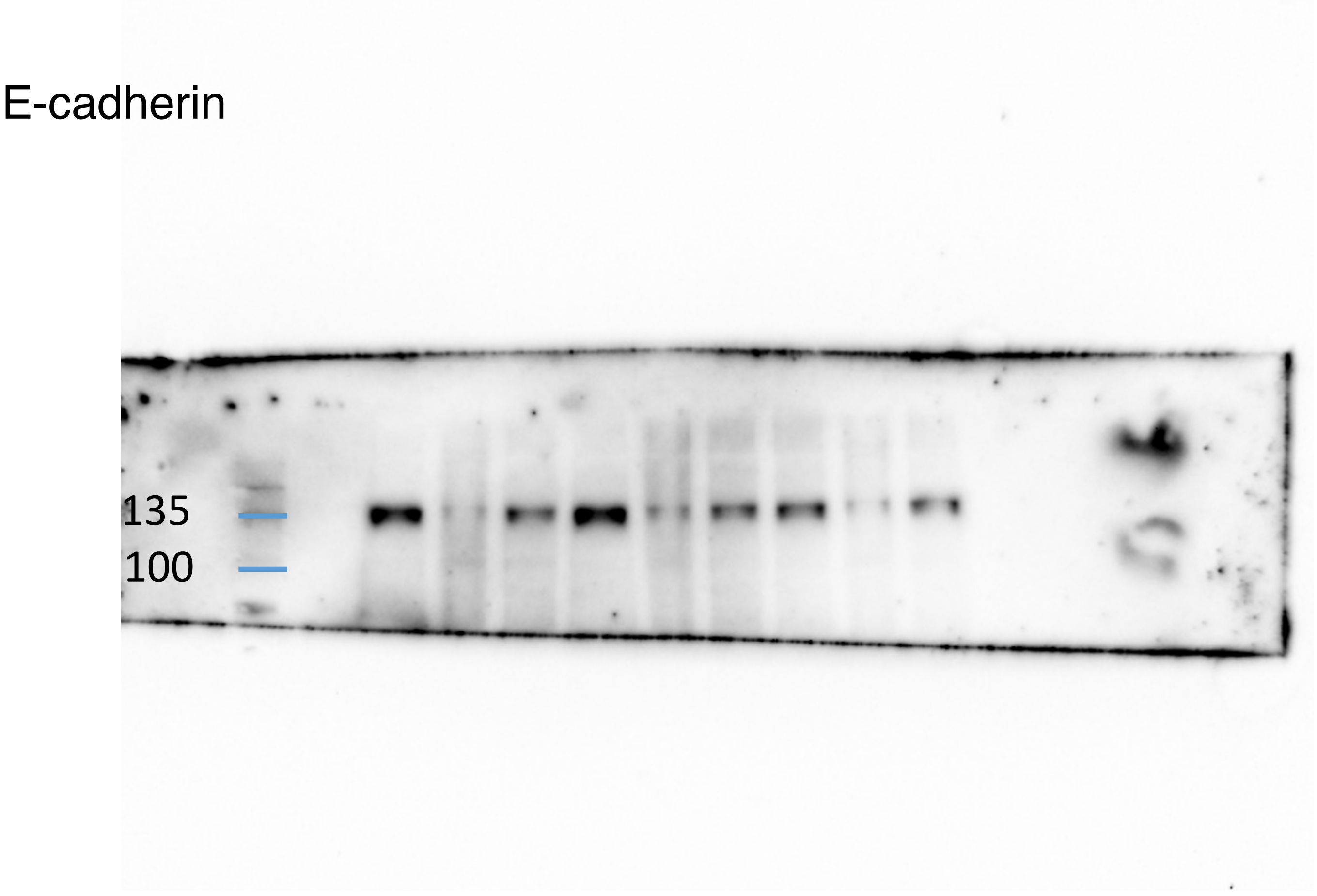

E-cadherin
135
100

N-cadherin

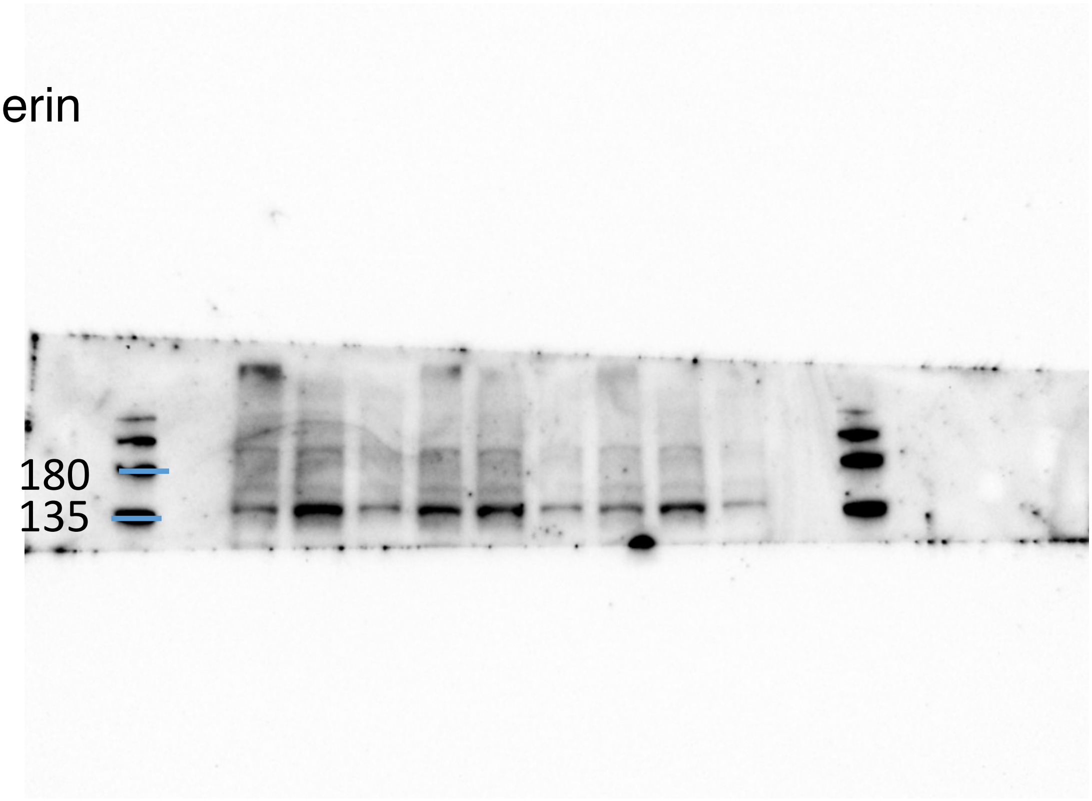

Vimentin

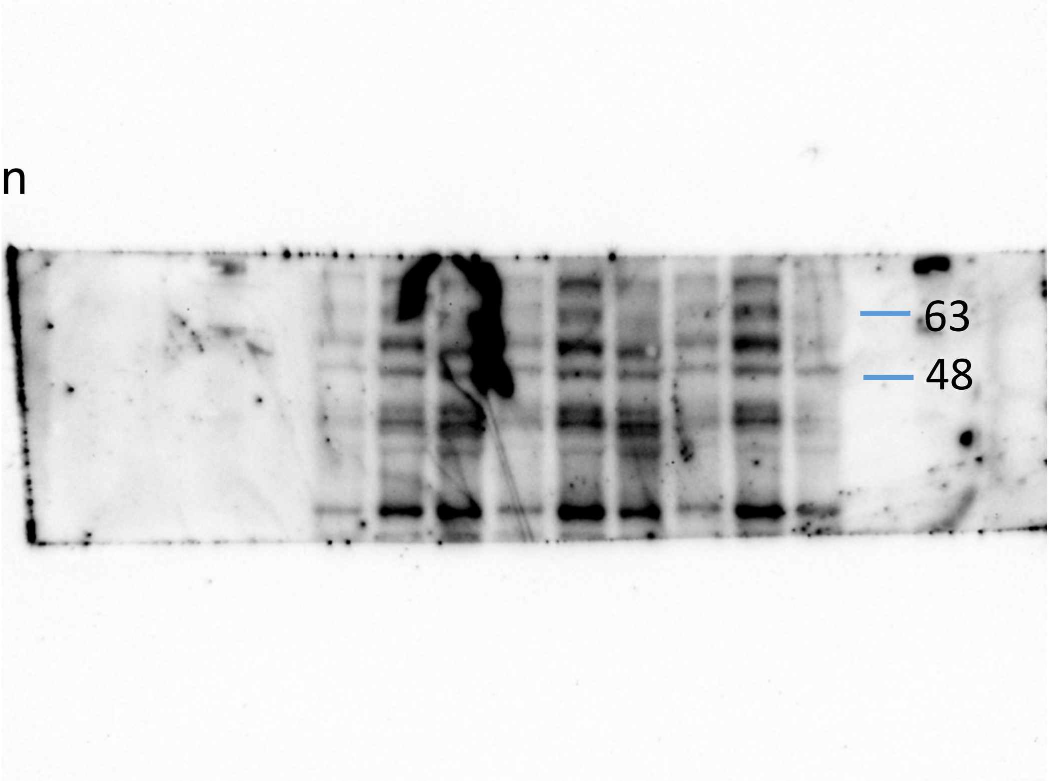

Snail

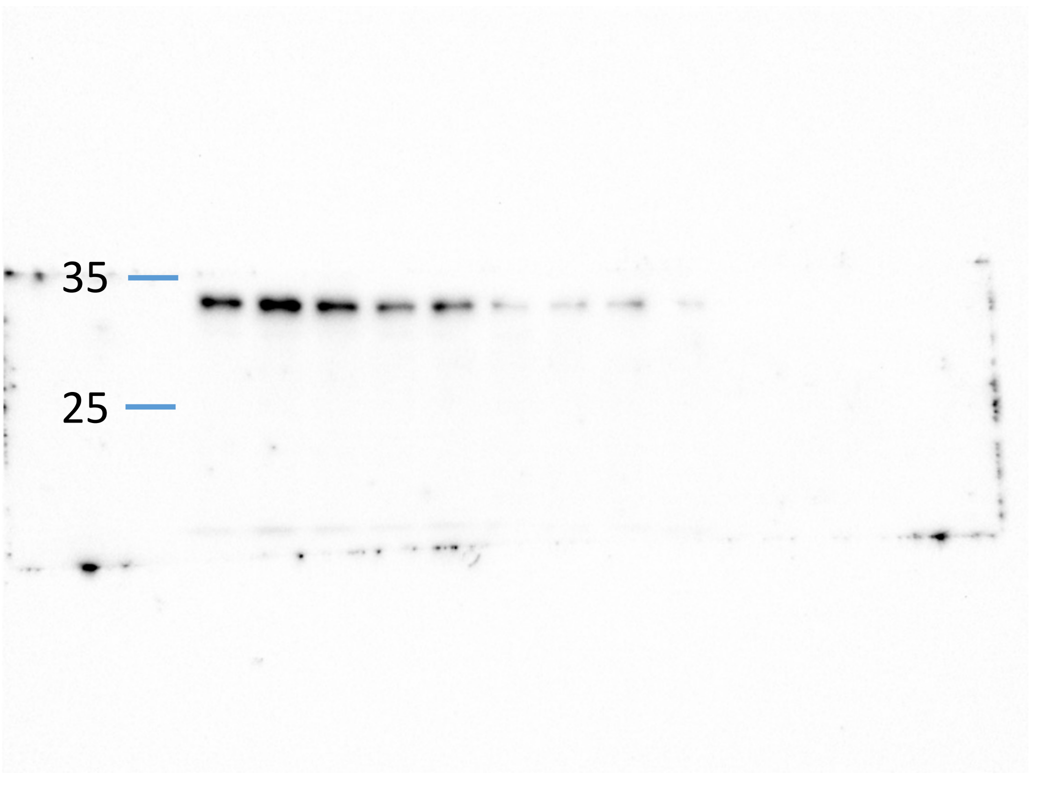

α-tubulin

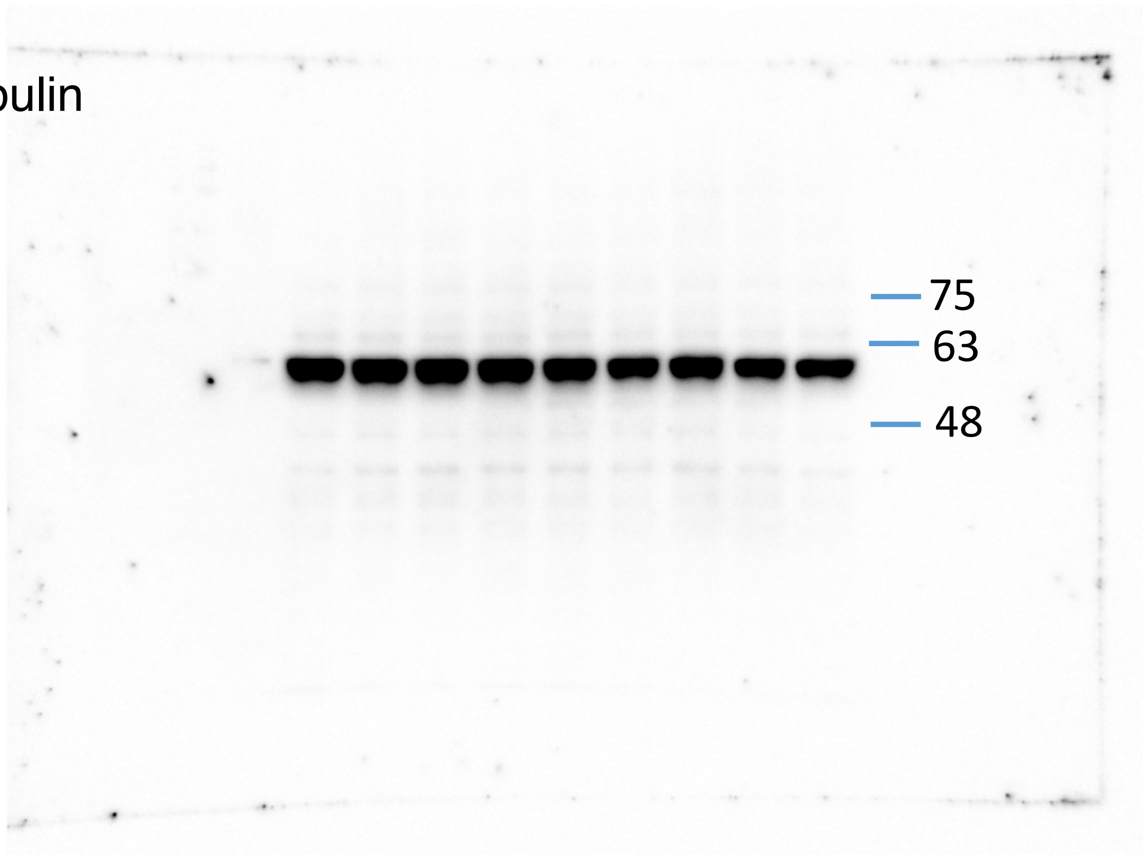

Supplement: Supplementary file 3 — Supplementary Material 3 [file 12944_2023_1930_MOESM3_ESM.pdf]
