# Supplementary material for: Ceramide synthase 4 overexpression exerts oncogenic properties in breast cancer
Source: Lipids Health Dis. 2023 Oct 26;22:183. doi: 10.1186/s12944-023-01930-z (PMC10605224; doi:10.1186/s12944-023-01930-z)
Supplement: Supplementary file 4 — Supplementary Material 4 [file 12944_2023_1930_MOESM4_ESM.docx]

**
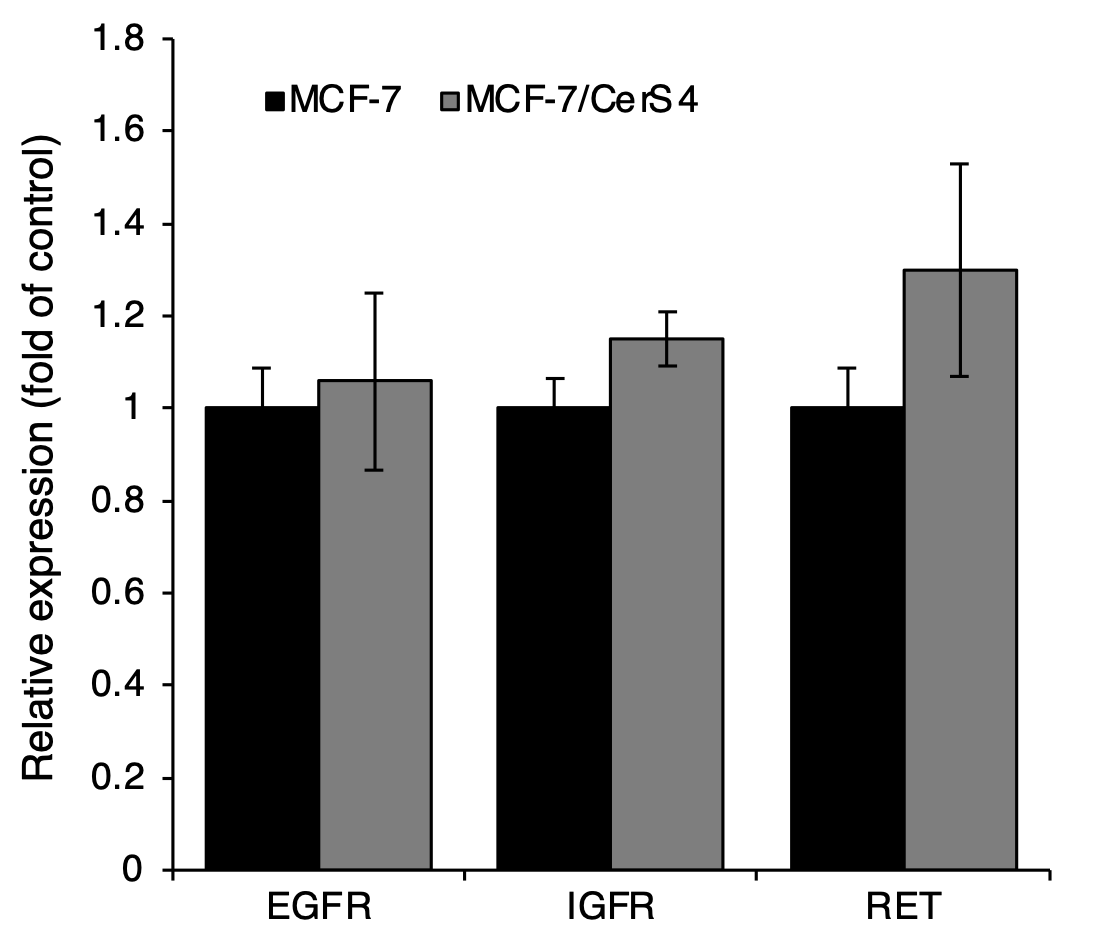
**

**Supplementary Figure S1. *CERS4* overexpression in MCF-7 cells did not affect the expression of EGFR, IGFR and RET.** Relative mRNA expression levels of EGFR, IGFR and RET in MCF-7 and CerS4 overexpressing MCF-7 cells (MCF-7/CerS4). EGFR, epidermal growth factor receptor, IGFR, insulin-like growth factor receptor, RET, ret proto-oncogene.


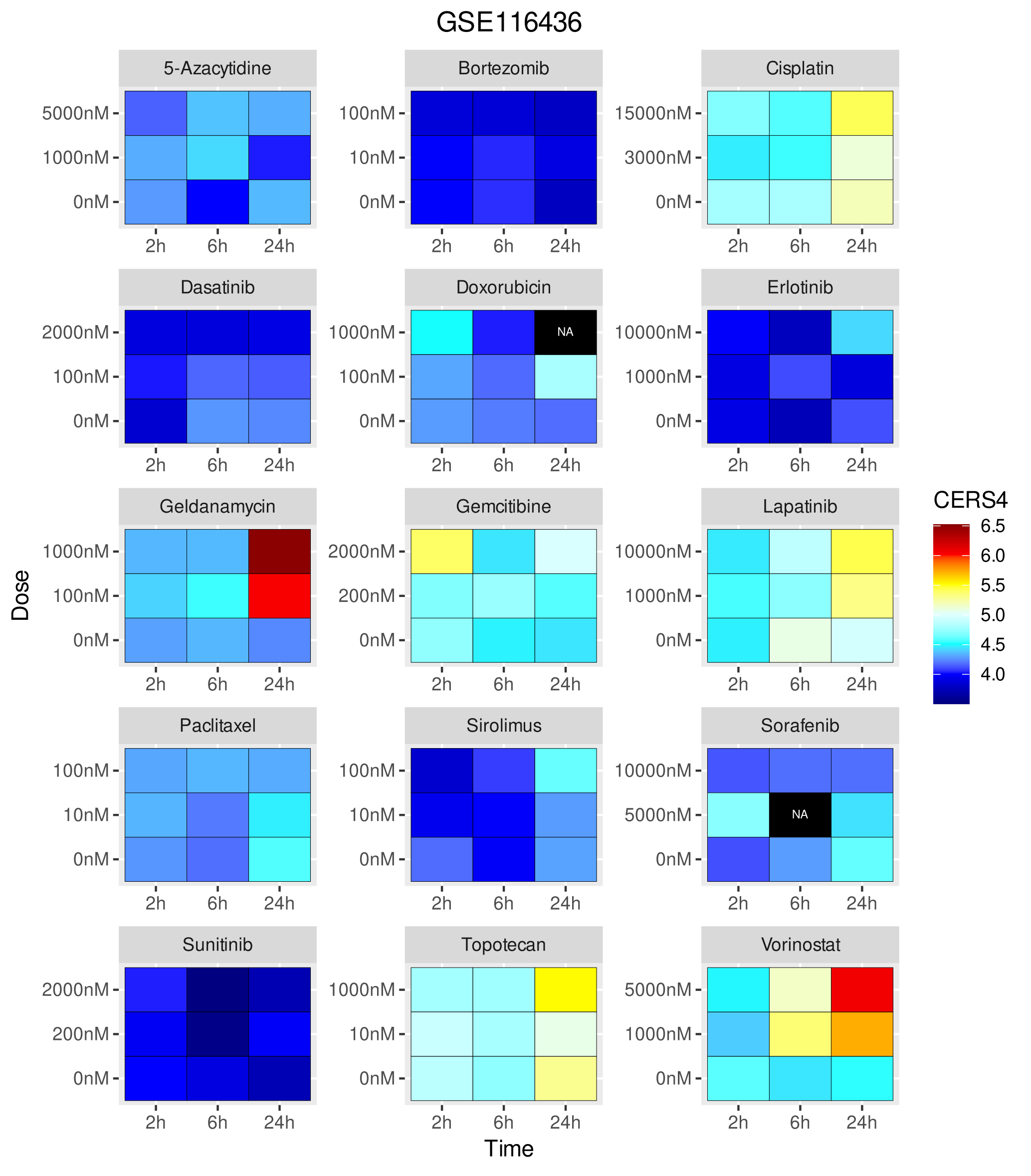
**Supplementary Figure S2. *CERS4* expression in MCF-7 cells after treatment with various chemotherapeutic agents.** Color shows relative expression of *CERS4* in MCF-7 cells. NA indicates missing value.

**Supplementary Table S1.** Primers used for real-time PCR.

| **Gene** | **Primer sequences** | **References** |
| --- | --- | --- |
| *CERS1* | F: 5′-CTTCTTCCATGACCCACCAT-3′  R: 5′-TAGAAGCTTCCCTGGAGCAG-3′ | [1] |
| *CERS2* | F: 5′-ATCGTCTTCGCCATTGTTTT-3′  R: 5′-GGCAGGATAGAGCTCCAGTG-3′ | [1] |
| *CERS3* | F: 5′-TCAGTAGCCAGCTTGTCCTC-3′  R: 5′-AGATGTGTCCCTCTGGTGAC-3′ | [1] |
| *CERS4* | F: 5′-GGAGGCCTGTAAGATGGTCA-3′  R: 5′-GAGGACCAGTCGGGTGTAGA-3′ | [1] |
| *CERS5* | F: 5′-GCTGCTCTTCGAGCGATTTATT-3′  R: 5′-GGGTTGGGCCTGATAAGGA-3′ | [1] |
| *CERS6* | F: 5′-TGCCATTCTGGAAAAGGTCT-3′  R: 5′-ATGCTTCGAACATCCCAGTC-3′ | [1] |
| *ESR1* | F: 5′-GTGCCTGGCTAGAGATCCTG-3′  R: 5′-GGTTCCTGTCCAAGAGCAAG-3′ |  |
| *ESR2* | F: 5′-TGCGGAACCTCAAAAGAGTC-3′  R: 5′-ACGGTTCCCACTAACCTTCC-3′ |  |
| *PGR* | F: 5′-CAGCCAGAGCCCACAATACA-3′  R: 5′-GTTGTGCTGCCCTTCCATTG-3′ | [2] |
| *ABCB1* | F: 5′-GCCAAAATATCAGCAGCCCA-3′  R: 5′-TTCCTTCCAATGTGTTCGGC-3′ |  |
| *ABCB4* | F: 5′-GAGCCCTCATCAGACAACCT-3′  R: 5′-CTTTGTCCAGGGCTTCTTGG-3′ |  |
| *ABCC1* | F: 5′-ACCTTCTGGTGGATCACAGG-3′  R: 5′-ACTTGTTCCGACGTGTCCTC-3′ |  |
| *ABCC2* | F: 5'-GAAGGCATTGACCCTATCCA-3′  R: 5′-TGGTCACATCCATGAGCTTC-3′ |  |
| *ABCC4* | F: 5′-TGAGATATCACAGCGCAACC-3′  R: 5′-TGGGGTCTCTGATGCCTTAT-3′ |  |
| *ABCC11* | F: 5′-AATGCTGGCCTGTTCTCCTA-3′  R: 5′-CAGTGGAGGGATGGTGTTCT-3′ |  |
| *ABCG2* | F: 5′-GGATGTCTAAGCAGGGACGA-3′  R: 5′-TTCCTGAGGCCAATAAGGTG-3′ |  |
| *SREBP1a* | F: 5′-TCAGCGAGGCGGCTTTGGAGCAG-3′  R: 5′-CATGTCTTCGATGTCGGTCAG-3′ | [3] |
| *SREBP1c* | F: 5′-GGAGGGGTAGGGCCAACGGCCT-3′  R: 5′-CATGTCTTCGAAAGTGCAATCC-3′ | [3] |
| *SREBP2* | F: 5′-CCAACATTCAGCACCACTCC-3′  R: 5′-TTGGACTTGAGGCTGAAGGA-3′ |  |
| *SCD1* | F: 5′-ATACCACCACCACCACCATT-3′  R: 5′-GCGAATGTCGTCTTCCAAGT-3′ |  |
| *LDLR* | F: 5′-TCATCCCCAACCTGAGGAAC-3′  R: 5′-CTGGGTGCTGCAGATCATTC-3′ |  |
| *HMGCR* | F: 5′-TTGCAGATGGGATGACTCGT-3′  R: 5′-CACTGCGAACCCTTCAGATG-3′ |  |
| *FASN* | F: 5′-ATCACAGGGACAACCTGGAG-3′  R: 5′-ACTCCACAGGTGGGAACAAG-3′ |  |
| *RET* | F: 5’-GGAAAAGTGGTCAAGGCAAC-3’  R: 5’-ATGTGGGTGGTTGACCTGCT-3’ | [4] |
| *EGFR* | F: 5’-GTTTGCCAAGGCACGAGTA-3’  F: 5’-CCAAGGACCACCTCACAGTT-3’ |  |
| *IGFR* | F: 5’-TGAGGATCAGCGAGAATGTG-3’  R: 5’-CTGAATCCGGGCTGTGTAGT-3’ |  |
| *GAPDH* | F: 5′-ACACCCACTCCTCCACCTTT-3′  R: 5′-TGCTGTAGCCAAATTCGTTG-3′ | [1] |

**Supplementary Table S2. Shared DEGs between MCF-7/CerS4 and MCF-7/ADR cells.**

Eighty-three upregulated and 13 downregulated DEGs were shared between MCF-7/CerS4 and MCF-7/ADR cells (GSE24460).

| **Category** | **No. of DEGs** | **Gene list** |
| --- | --- | --- |
| Shared upregulated DEGs between MCF-7/CerS4 and MCF-7/ADR cells | 83 | *ACTN1*, *ADM*, *ALDH1A3*, *ARHGDIB*, *ARL4D*, *ASPH*, *BAG2*, *C1R*, *CAD*, *CALD1*, *CCDC86*, *CDC20*, *CLGN*, *CLIP4*, *COTL1*, *CSRP2*, *CTPS1*, *CTSC*, *DBN1*, *DDX10*, *DUSP1*, *EIF5A*, *EIF6*, *EPB41L2*, *FAM216A*, *FHL1*, *FHL2*, *FN1*, *FOSL1*, *GADD45A*, *GALE*, *GBP1*, *GEM*, *HBEGF*, *HLA*-*DRB1*, *ICAM1*, *IGFBP3*, *IL32*, *ITGA5*, *JUN*, *KLF2*, *KLF6*, *LGALS3BP*, *LRRC15*, *MAFF*, *MAPKAPK3*, *MAPRE2*, *MARCKS*, *ME3*, *MSX1*, *NRGN*, *OGFRL1*, *PCLO*, *PEG10*, *PLAUR*, *PLEKHO2*, *POLR2L*, *PSAT1*, *PSME3*, *PYGL*, *RAC2*, *RAI14*, *RGS2*, *RHOB*, *RPP25*, *RRP7A*, *SAMD4A*, *SNRPB*, *SOWAHC*, *ST6GAL1*, *STEAP1*, *STEAP3*, *TFPI2*, *TGFBI*, *TGM2*, *TK1*, *TUBB2A*, *TUBB3*, *TUBB6*, *TUBG1*, *TXNRD1*, *WDR77*, *WLS* |
| Shared downregulated DEGs between MCF-7/CerS4 and MCF-7/ADR cells | 13 | *ABCG1*, *C10orf88*, *EFHD1*, *FAXDC2*, *LYPD3*, *METTL7A*, *NEBL*, *PRLR*, *RAB27B*, *S100A14*, *SCD*, *SELENBP1*, *SRSF1* |

Reference

1. Kim MH, Park JW, Lee EJ, Kim S, Shin SH, Ahn JH, Jung Y, Park I, Park WJ: **C16‑ceramide and sphingosine 1‑phosphate/S1PR2 have opposite effects on cell growth through mTOR signaling pathway regulation.** *Oncol Rep* 2018, **40:**2977-2987.

2. Wang Y, Sun J, Zhang K, Hu X, Sun Y, Sheng J, Fu X: **Black tea and D. candidum extracts play estrogenic activity via estrogen receptor α-dependent signaling pathway.** *Am J Transl Res* 2018, **10:**114-125.

3. Shimomura I, Shimano H, Horton JD, Goldstein JL, Brown MS: **Differential expression of exons 1a and 1c in mRNAs for sterol regulatory element binding protein-1 in human and mouse organs and cultured cells.** *J Clin Invest* 1997, **99:**838-845.

4. Liu T, Bohlken A, Kuljaca S, Lee M, Nguyen T, Smith S, Cheung B, Norris MD, Haber M, Holloway AJ, et al: **The retinoid anticancer signal: mechanisms of target gene regulation.** *Br J Cancer* 2005, **93:**310-318.
